# Mechanism of action and evaluation of ratiometric probes for uric acid using lanthanide complexes with tetraazatriphenylene sensitisers†

**DOI:** 10.1039/d4sc05743k

**Published:** 2024-11-07

**Authors:** Xinyi Wen, Huishan Li, Zhijie Ju, Renren Deng, David Parker

**Affiliations:** a Department of Chemistry, Hong Kong Baptist University Kowloon Tong 999077 Hong Kong China; b State Key Laboratory of Silicon and Advanced Semiconductor Materials, Institute for Composites Science Innovation, School of Materials Science and Engineering, Zhejiang University Hangzhou 310058 China davidparker@hkbu.edu.hk

## Abstract

A series of new ligands has been prepared that incorporate electron-poor aromatic moieties (dpqMe_2_ and dpqPh_2_ chromophores) into tetraazacyclododecane or triazacyclononane based complex structures, and the time-dependent photophysical properties of their Eu(iii) and Tb(iii) complexes evaluated for the selective and rapid ratiometric analysis of urate in diluted serum solution, together with mechanistic studies probing the nature of the intermediate exciplex and the excited state dynamics using transient absorption spectroscopy.

## Abstract 2

Six Eu(iii) and Tb(iii) complexes are evaluated for the rapid ratiometric analysis of urate in diluted serum, together with mechanistic studies probing the intermediate exciplex and the excited state dynamics by transient absorption spectroscopy.

## Graphical abstract

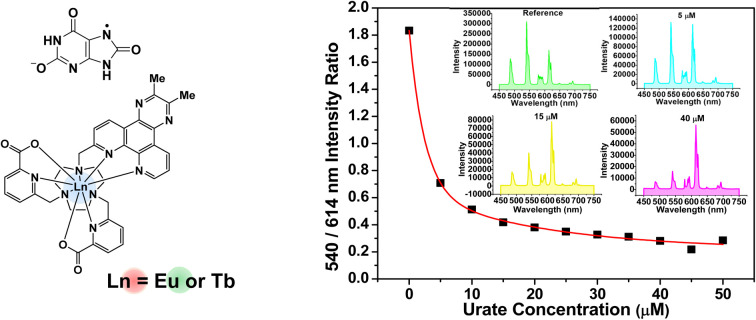

## Introduction

Uric acid is the end-product of purine metabolism and the concentration of its sodium salt in serum ranges between 22–77 μg mL^−1^ (0.13 to 0.46 mM) compared with 1.5 to 4.5 mM in urine.^1,2^ It is an important scavenger of reactive oxygen species and an essential antioxidant that operates in tandem with the more soluble and hydrophilic species, ascorbate. High levels of uric acid contribute to the development and progression of gout and certain metabolic syndromes, and it has been suggested to be a useful biomarker to signal the onset of cardiovascular disease and certain cancers.^1,2^ Hyperuricemia (>70 μg mL^−1^) is regarded as an independent health risk factor, as well as a biomarker for hypertension, atrial fibrillation, heart failure, atherosclerosis and chronic kidney disease.^3–6^ The prevalence of hyperuricaemia is increasing globally, although administration of the xanthine oxidase inhibitor, allopurinol, improves cardiovascular outcomes in patients with heart failure, coronary heart disease and type 2 diabetes.

Ascorbate is an anion commonly found in aqueous body compartments and serves as an anti-oxidant and pro-oxidant to maintain redox homeostasis.^7^ Its concentration varies from near zero in erythrocytes to 10 mM in neurons.^8^ Despite abundant evidence showing that urate and ascorbate are important regulators of metabolic and genetic processes, reliable and simple optical tools to directly monitor the concentration of such small bioactive anions in real time are lacking both *in vitro* and *in vivo*.^2^ Lanthanide probes for urate detection based on small molecules are rare,^2*a*^ and most recent examples are based on metal–organic frameworks (MOFs),^2*g*^ polymers^2*j*^ and related ‘nanomaterials’.^2*l*^ Most of these examples are based on europium intensity changes and are limited by calibration problems. In our work, the ratio of red (Eu, 614 nm) to green (Tb, 540 nm) luminescence intensity is used to determine the urate concentration, using mixtures of Tb and Eu complexes with a common ligand. The variable effects that may occur in the spectral analysis of biological samples, such as light scattering, autofluorescence and static quenching processes, are intrinsically corrected by taking the ratio of the long-lived red/green emission intensities.

Highly emissive complexes of the lanthanide(iii) ions have attracted considerable interest, owing to their unique advantages over other luminescent probes, notably their long luminescence lifetimes and large pseudo-Stokes' shifts.^9–12^ Based on earlier work by Poole *et al.*, it was established that electron rich species such as urate, ascorbate, and certain catecholates, can deactivate the lanthanide excited state efficiently, particularly using systems which incorporate an electron poor sensitising group, like a tetra-azatriphenylene (dipyridoquinoxaline, dpq/dpqC) or an azaxanthone moiety.^13,14^ It was hypothesised that this dynamic quenching process involves formation of an excited state complex (exciplex) between the ligand triplet excited state and the electron-rich quencher.^13^ Ascorbate (*E*_1/2_ = +0.30 V, for the one electron process, 298 K, pH 7; p*K*_a_ 4.2), iodide (*E*_1/2_ = +0.51 V), and urate (*E*_1/2_ = 0.59 V, p*K*_a_ 5.5) can each deactivate the triplet excited state of the sensitising moiety, leading to a reduction in both the lanthanide emission lifetime and intensity. Such a dynamic quenching process is often characterised by its Stern–Volmer quenching constant, that gives the concentration of reductant needed to lower the lifetime or emission intensity to 50% of its original value.

In the earlier work, terbium complexes were observed to be significantly more sensitive to excited state quenching than their Eu analogues, due to the higher energy of the Tb ^5^D_4_ excited state compared to Eu ^5^D_0_ (20 400 *vs.* 17 200 cm^−1^).^13–15^ The ratio of the red (Eu, 616 nm) to green (Tb, 545 nm) luminescence intensity was measured in diluted urine and serum for samples of varying urate concentration, using mixtures of Tb and Eu complexes with a common ligand structure.

**Fig. 1:**
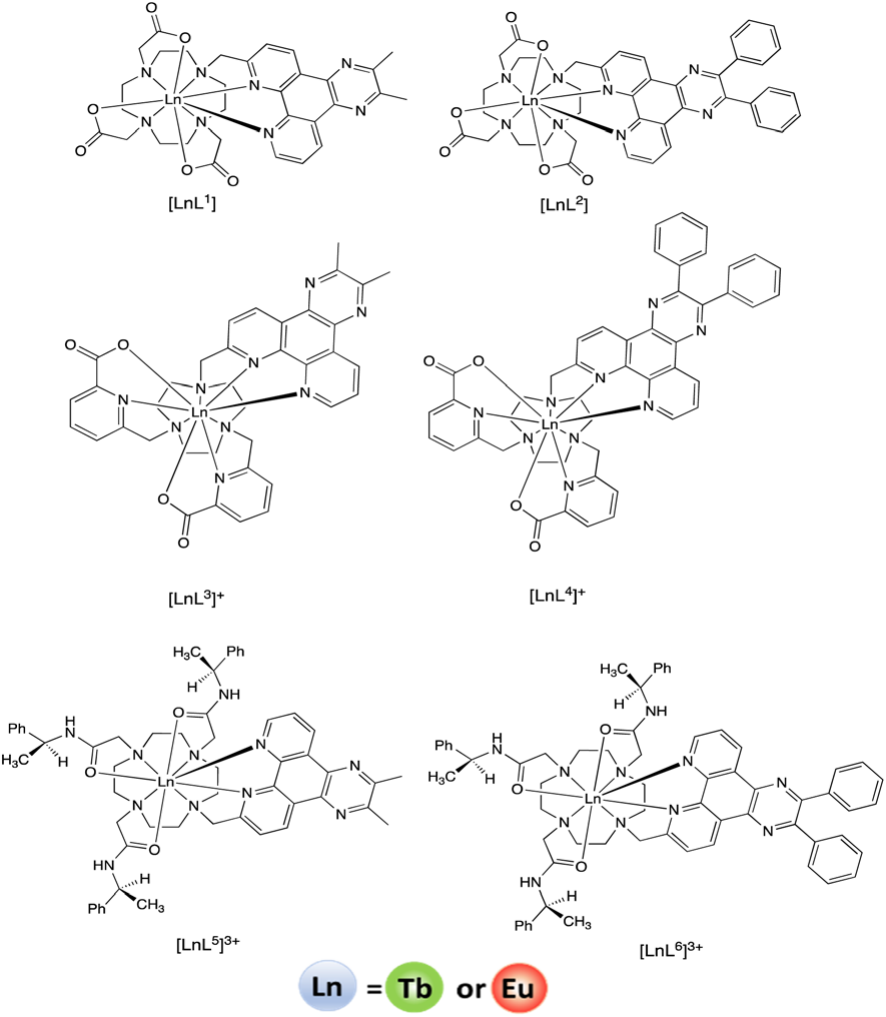
Lanthanide complexes with tetra-azatriphenylene sensitisers.

Here, we present six new nonadentate ligands (Fig. 1) that integrate dpqPh_2_ or dpqMe_2_ chromophores with 1,4,7,10-tetraazacyclododecane (12-N_4_) and 1,4,7-triazacyclononane (9-N_3_) structures. These nonadentate ligands provide a rigid and well-defined coordination environment and exclude water from the primary coordination sphere, thereby averting vibrational quenching of the metal excited state by coordinated OH oscillators, giving higher overall emission quantum yields. The Eu and Tb complexes of each ligand have been synthesised, with a view to examining their suitability as probes for the ratiometric analysis of urate *in vitro*. Such work could pave the way for new approaches to allow the determination of urate in living cells, *e.g.* using spectral imaging in microscopy, where its concentration is difficult to measure and the concentration in different organelles is unknown.

## Results and discussion

**Scheme 1:**
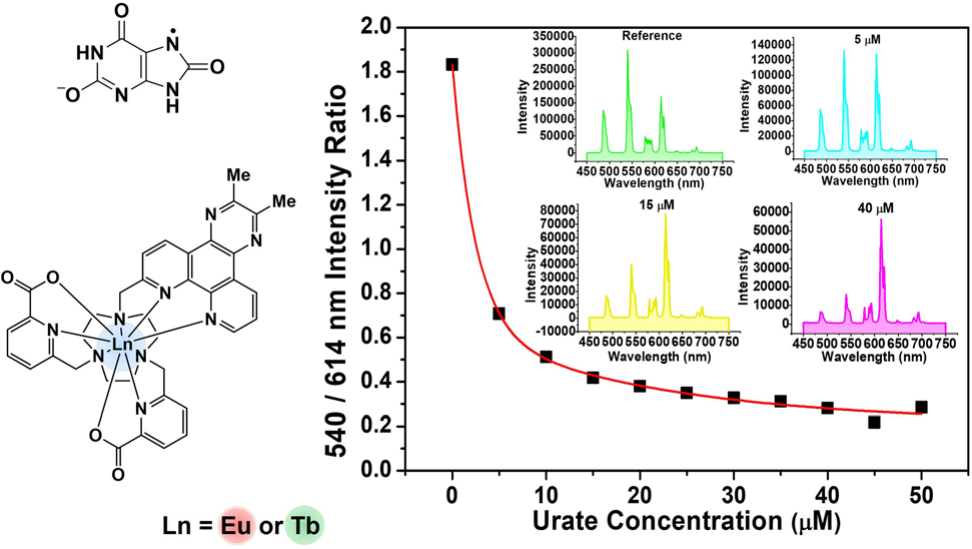
Synthetic procedures for L^3,4^.

**Fig. 2:**
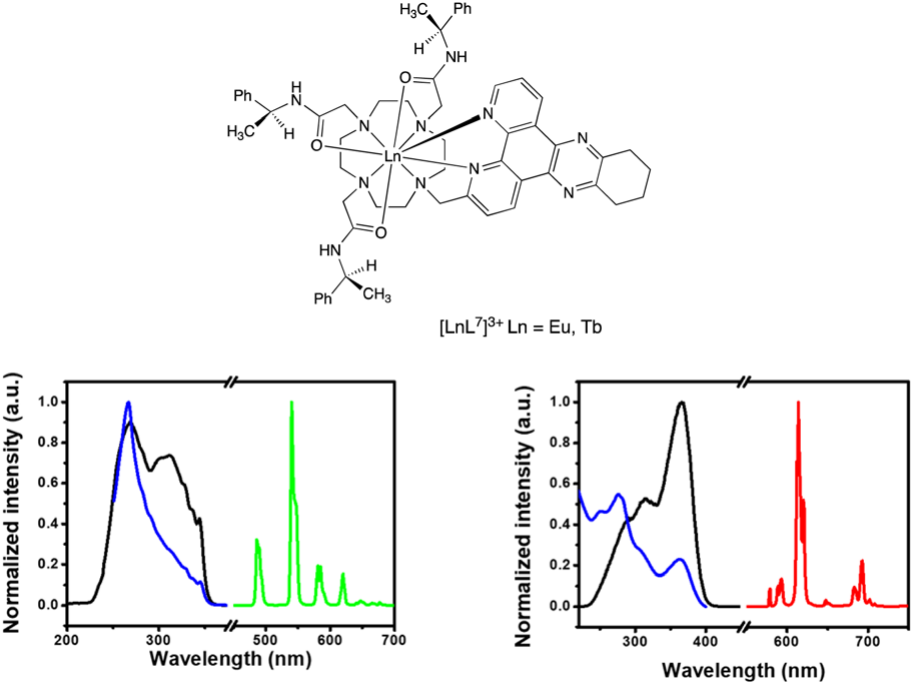
(left) Absorption (blue), excitation (black) and emission (green) spectra for [TbL^3^]^+^: 298 K, H_2_O, *λ*_abs_ = 345 nm; *λ*_ex_ = 345 nm; *λ*_em_ = 540 nm (excitation and emission slits 1 nm); *τ*_H_2_O_ = 1.60 ms. (right) Absorption (blue), excitation (black) and emission (red) spectra for [EuL^4^]^+^, (H_2_O, 298 K), *λ*_abs_ = 363 nm; *λ*_ex_ = 363 nm; *λ*_em_ = 614 nm (excitation and emission slits 1 nm); *τ*_H_2_O_ = 0.95 ms.

The chromophores and the lanthanide(iii) complexes based on 12-N_4_ (cyclen) (*i.e.* [LnL^1,2^] and [LnL^5,6^]^3+^) were synthesised by following previously reported methods, with only minor adaptations (Fig. 1).^13–15^ Thus, the intermediacy of a dpq-benzylic mesylate (Experimental) was preferred to the use of the corresponding bromide or chloride. In each case, the final complexes were purified by reverse phase HPLC, and were characterized by accurate mass ES or MALDI-TOFMS. Complexes based on 1,4,7-triazacyclononane (9-N_3_) with such a dpq chromophore have not been made before, although there is ample precedent for the synthesis of related nonadentate ligands, with up to three 2-picolinate donors, based on 1,4,7-triazacyclononane.^16–20^ The synthesis of the ligands L^3^ and L^4^ followed the same route involving the intermediacy of mono-Boc-9-N_3_ (Scheme 1). For Eu complexes with the parent tetraazatriphenylene (or ‘dipyridoquinoxaline’; ‘dpq’) ring, the introduction of two phenyl rings, replacing the Me groups (or a cyclohexyl ring: ‘dpqC’), extends the degree of conjugation, increases the molar absorptivity and permits more efficient longer wavelength excitation in the range 355 to 375 nm, with no significant change in the Eu emission lifetime. The Tb complexes (except with the dpqPh_2_ chromophore) exhibit longer metal-based lifetimes and higher quantum yields compared to Eu analogues, as found in the earlier work (Table 1 and Fig. 2).^13–15^ Quantum yield measurements were made based on established methods and the chloride salts of the analogous tri-amide complexes, [EuPh_3_dpqC]^3+^ (*λ*_max_ (H_2_O) 348 nm, *Φ* (H_2_O) = 16%) and [TbPh_3_dpqC]^3+^ (*λ*_max_ (H_2_O) 348 nm, *Φ* (H_2_O) = 40%) were chosen as references,^21^ (C = cyclohexyl), [LnL^7^]^3+^.

**Table 1:** Photophysical data^a^ for europium(iii) and terbium(iii)^b^ complexes (296 K)

Complex	*λ*_abs_ (H_2_O)/nm (log *ε*/M^−1^ cm^−1^)	*τ* (H_2_O)/ms	*Φ* (%)
[EuL^1^]	345 (3.65)	1.09	16
[TbL^1^]	345 (3.65)	1.86	50
[EuL^2^]	363 (4.11)^a^	1.06	18
[EuL^3^]^+^	345 (3.51)	0.97	14
[TbL^3^]^+^	345 (3.78)	1.60	30
[EuL^4^]^+^	363 (4.48)	0.95	30
[EuL^5^]^3+^	345 (3.69)	1.04	11
[TbL^5^]^3+^	345 (3.62)	2.20	30
[EuL^6^]^3+^	364 (4.23)	1.00	11

a Measurements were performed in the mixture of MeOH and H_2_O to allow comparison (5 : 95).

b [TbL^2^] shows no metal-based emission at ambient temperature in water/MeOH mixtures.

### Photoluminescence behaviour

**Fig. 3:**
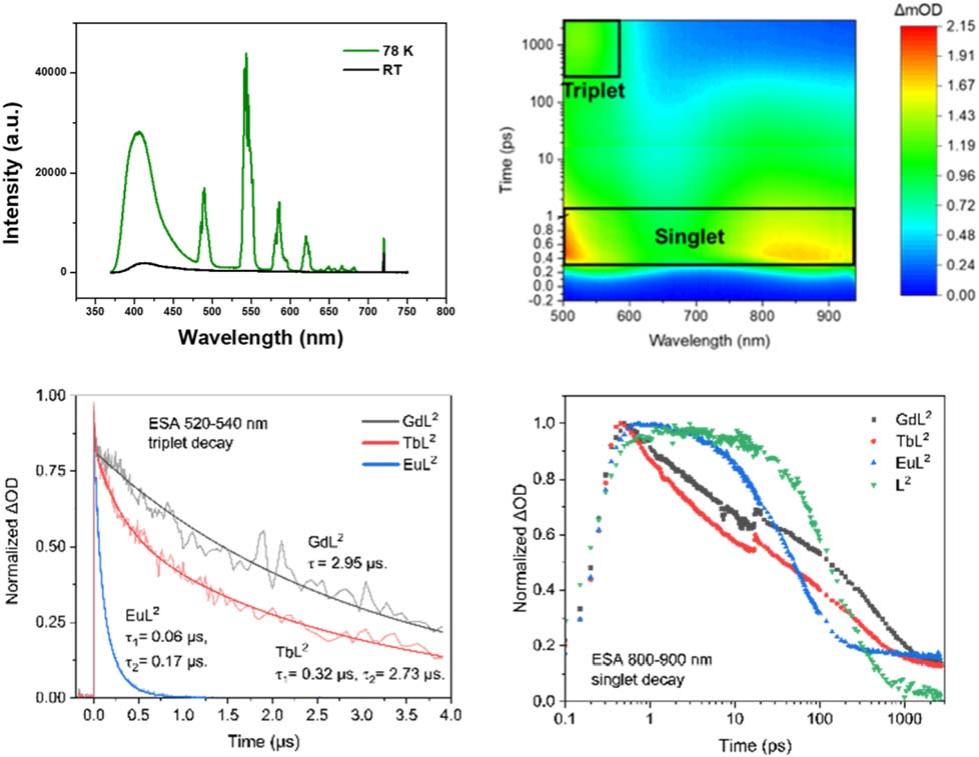
(upper) The emission spectrum of [TbL^2^] in ethanol/methanol (1/4, v/v) at room temperature (black) and 78 K (green); (right) 2D pseudo-colour transient absorption spectra for [TbL^2^]; [GdL^2^] showed very similar behaviour; (lower) kinetics of triplet decay of [LnL^2^] probed at 520–540 nm (average); (lowest) kinetics of singlet decay for L^2^ and [LnL^2^] (*λ*_exc_ 350 nm); lifetimes were as follows: L^2^ 191 ps; [EuL^2^] 52 ps; [GdL^2^] 225 ps; [TbL^2^] 406 ps.

The photoluminescence properties of L^2^ and its Eu, Gd and Tb complexes were studied to find out about the salient excited states and their dynamics (Table 1 and Fig. 3). Both L^2^ and its lanthanide complexes showed very fast photoluminescence (PL) decay, with lifetimes of less than 2 nanoseconds (Fig. S1 to S5†). The fast PL decay was beyond the time resolution of the equipment. At room temperature, [TbL^2^] did not sensitise emission from the terbium excited state under both oxygen and nitrogen atmospheres (Fig. S3†), owing to fast reverse energy transfer and the fast decay of the ligand triplet state.

At 78 K under a nitrogen atmosphere, when the triplet state is protected from vibrational and oxygen quenching, the emission from the ^5^D_4_ excited state of Tb^3+^ became observable (Fig. 3). However, the fluorescence of the ligand in [TbL^2^] remained prominent, consistent with poor sensitisation (*i.e.* low energy transfer) efficiency. The 78 K PL spectrum of [GdL^2^] only shows emission from the ligand, as expected because of the much higher energy of the ^6^I_J_ and ^6^P_J_ excited states of Gd^3+^, with strong ligand fluorescence at 420 nm again observed, but no measurable ligand phosphorescence.

### Picosecond and nanosecond transient absorption studies

Transient absorption (TA) spectral analyses confirmed the fast decay of the singlet states in L^2^ and its lanthanide complexes. The TA spectra were first studied in the picosecond time range (psTA), (Fig. S6–S9†). The excited state absorption (ESA) spectra of the excited singlet state revealed two bands centred around 500 nm and 850 nm. The dynamics of the excited singlet states (Fig. S10, S11† and Fig. 3) were assessed observing the ESA signal between 800 to 900 nm. As a result of the heavy metal effect, L^2^ has the slowest singlet decay rate compared with its lanthanide complexes. The ESA spectra of the singlet state at 0.5 ps were almost the same for [EuL^2^], [GdL^2^] and [TbL^2^], confirming their common origin from the ligand (Fig. S10†). At about 1500 ps, the singlet starts to transform into the triplet state, characterised by an ESA signal at 530 nm for each lanthanide complex (Fig. S11†). The decay of the singlet excited state was ‘tail-fitted’ to a dominant single exponential model to give lifetime values of 225 ps, 406 ps and 52 ps for [TbL^2^], [GdL^2^] and [EuL^2^] respectively. Faster rates of intersystem crossing for Eu compared to Gd have also been observed recently,^22,23^ in lanthanide complexes with an internal charge transfer excited state, where the values observed by monitoring the rise time of the triplet state were over 100 times faster than those observed here. Fast inter-system crossing has been identified as being important for high sensitisation efficiency.^24–29^

From the TA spectra in the nanosecond time range (nsTA), more information concerning the triplet states was acquired. The ESA from triplet states dominated the nsTA spectra (Fig. S12–S14†). The decay of the triplet state was fitted using the ESA signal from 520 to 540 nm (Fig. 3). The results indicated that [GdL^2^] had the longest triplet lifetime, with a value of 2.95 μs. The triplet lifetime of [TbL^2^] was fitted to two exponential decays, with values of 0.32 μs and 2.73 μs that can be reasonably assigned to non-radiative decay of the triplet excited state (lifetime 2.73 μs) and to energy transfer from the ligand to the ^5^D_4_ excited state of Tb^3+^ (*i.e. k*_ET_ = 3 × 10^6^ s^−1^). Similarly, the triplet lifetime of [EuL^2^] was fitted to two exponential decays, with lifetimes of 0.06 μs and 0.17 μs, indicating efficient energy transfer from the ligand to the ^5^D_1_ (*k*_ET_ = 1.7 × 10^7^ s^−1^) and ^5^D_0_ excited states (*k*_ET_ = 6 × 10^6^ s^−1^) of Eu^3+^, respectively.^25^

### Luminescence quenching studies with urate, iodide and ascorbate

**Fig. 4:**
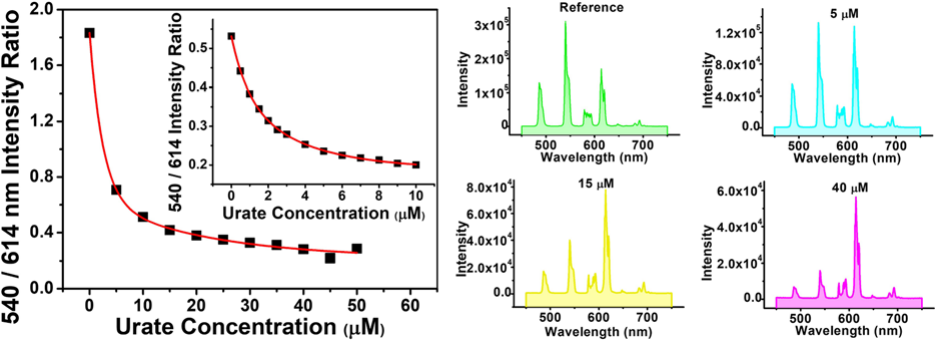
(Left) Variation of the 540 nm ([TbL^3^])/614 nm ([EuL^3^]) emission intensities as a function of sodium urate concentration (pH 7.4, 298 K, 0.1 M HEPES buffer). The inset shows the calibration curve measuring urate concentration in diluted human serum, (*λ*_exc_ 345 nm, pH 7.4, 296 K). (Right) Selected emission spectra showing the differing changes in intensity as urate is added.

The earlier work revealed that the sensitivity of excited state quenching in such dpq lanthanide complexes did not follow the ease of oxidation of the quenching species; quenching by urate was the most effective, notwithstanding its higher one electron oxidation potential.^13,14^ Such behaviour is in line with a dynamic quenching process, involving the intermediacy of an exciplex formed between the electron poor dpq triplet excited state and the planar urate anion.^15^ In this situation, there is the possibility for a photo-equilibrium to be established involving back energy transfer from the Ln* to the lower vibrational levels of the exciplex, reducing both overall emission intensity and the Ln* lifetimes. Measurements of the relative sensitivity of each complex to urate quenching were undertaken systematically (Table 2 and Fig. 4).

**Table 2:** Stern–Volmer quenching constants* (*K*_SV_^−1^/microM) for dynamic quenching of the Ln excited state (pH 7.4, 0.1 M HEPES, 298 K)

Complex	Urate (μM)
[EuL^1^]	96
[TbL^1^]	5.0
[EuL^2^]	86
[EuL^3^]^+^	20
[TbL^3^]^+^	3.0
[EuL^4^]^+^	23
[EuL^5^]^3+^	60
[TbL^5^]^3+^	22
[EuL^6^]^3+^	50

Data obtained by studying the reduction in the emission intensity of the ^5^D_4_ (Tb) and ^5^D_0_ (Eu) excited states for each complex by urate are collated in Table 2. The apparent Stern–Volmer quenching constant was used to estimate the quenching sensitivity. Changing the substituent from Me to Ph in the chromophore did not influence quenching sensitivity significantly. In the absence of a steric effect, an overall positive charge favoured quenching slightly, by encouraging exciplex formation *via* coulombic attraction with the urate mono-anion. Quenching by urate was suppressed by steric shielding in systems with more bulky ligand substituents, in agreement with earlier studies.^13,14^ Such behaviour is revealed, for example, by comparing the cationic 12-N_4_ based triamide complexes ([LnL^5,6^]^3+^ with the more open ‘DO3A’ triacetate systems, [LnL^1,2^]). Rather surprisingly, it was found that lanthanide complexes based on the 9-N_3_ structures ([LnL^3,4^]^+^) were more sensitive to urate quenching compared to those systems based on the 12-N_4_ ring, although this effect may be confounded by the impact of opposing coulombic and steric effects.

To assess the suitability of [EuL^3^/TbL^3^]^+^ as probes for ratiometric analysis of urate in serum, a 1 : 1 mixture of Eu and Tb complexes was used, measuring the variation of the ratio of their emission intensities (Tb at 540 nm; Eu at 614 nm) as a function of sodium urate concentration. Solutions containing 30 μM concentrations of [TbL^3^]^+^ and [EuL^3^]^+^ (absorbance 0.1 at 345 nm, pH 7.4 in 0.1 M HEPES) and a stock solution of urate (pH 7.4 in 0.1 M HEPES) were prepared. Samples of human serum were diluted 50-fold and triplicate samples were prepared. Equal volumes of the complex mixture and diluted urate solution were added to cover the urate concentration range of 0 to 50 μM. For each data point, three separate spectra were recorded (*λ*_exc_ 345 nm) and averaged, and the intensity ratio of the 540/614 bands was determined. A plot of the ratio of emission intensities *vs.* urate concentration was made and was fitted to a bi-exponential model to create a calibration curve (Fig. 4, inset). Less than 1% variance was found in the measured intensity ratio for a given urate concentration. The small differences in behaviour between buffer and diluted serum media can be rationalised in terms of changes in ionic strength (*vide infra*) and the impact of serum components. Such behaviour reaffirms the need to calibrate the method in the medium of interest.

In extending this work, quenching studies with iodide and ascorbate for [LnL^3^]^+^ (Ln = Eu/Tb) and [EuL^4^]^+^ were performed to establish their relative quenching sensitivities (Table 3). The terbium excited state was quenched by iodide ten times more effectively compared to Eu analogues, while the Eu and Tb complexes were over a 100 and a thousand-fold more sensitive to quenching by urate compared to ascorbate respectively. In addition, the Eu complex with a dpqPh_2_ chromophore, [EuL^4^]^+^, was more sensitive to quenching by iodide, compared to the analogue with a dpqMe_2_ chromophore, while each exhibited similar sensitivities to urate. It should be noted that the variation of emission intensity with added ascorbate for [EuL^4^]^+^ showed no obvious trend. Quenching was not very efficient overall, and an apparent Stern–Volmer quenching constant could not be derived from the experimental data, in this case.

**Table 3:** Stern–Volmer quenching constants (*K*_SV_^−1^) for dynamic quenching of the Ln* excited state (pH 7.4, 0.1 M HEPES, 298 K)

Complex	Ascorbate (mM)	Iodide (mM)	Urate (μM)
[EuL^3^]^+^	2.63	75.1	20
[TbL^3^]^+^	3.68	7.42	3.0
[EuL^4^]^+^	—	17.4	23

### Quenching mechanism: temperature and ionic strength dependence

Based on previous work, the dynamic quenching of Eu and Tb complexes by electron-rich substances (such as urate, ascorbate and catecholates) in systems with an electron-poor sensitising group, like ‘dpq’, can be hypothesised to involve the formation of an intermolecular exciplex. Accordingly, temperature and ionic strength dependence measurements were performed to seek support for the hypothesis of formation of such an excited state complex. In a classical dynamic (collisional) quenching model, higher temperatures should lead to a greater degree of quenching. In contrast, exciplex formation brings together two species, in an encounter that is entropically unfavourable (*i.e.* a negative Δ*S* change). Hence, higher temperatures should lead to less quenching and an increased emission intensity.

### Europium complex quenching behaviour

**Fig. 5:**
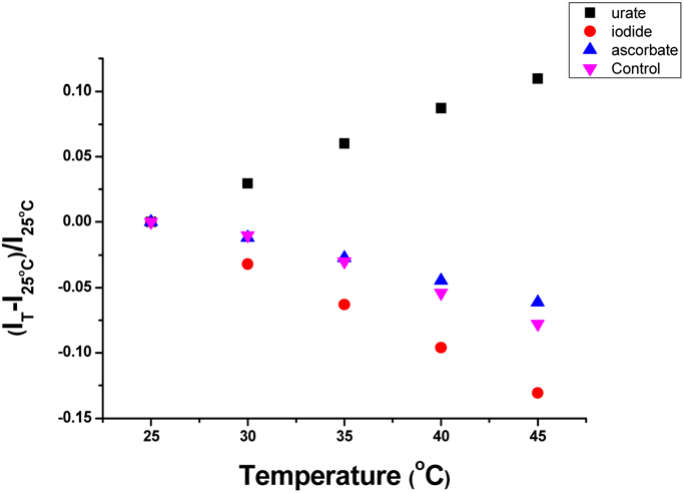
Temperature dependence of emission intensity change for [EuL^3^]^+^ in the presence of: (a) urate (50 μM, black squares); (b) iodide (10 mM, red); (c) ascorbate (250 μM, blue); (d) control with no added quencher (purple), [15 μM complex, pH 7.4, 0.1 M HEPES, 298 K].

The variation of the emission intensity of [EuL^3^]^+^ with temperature was measured under standard conditions (15 μM complex, pH 7.4, 0.1 M HEPES) in the presence of a fixed concentration of urate, iodide and ascorbate (Fig. 5). In the presence of urate, the emission intensity increased by 10% as the temperature was increased from 25 °C to 45 °C, consistent with the hypothesis of exciplex formation between the electron poor ‘dpq’ moiety and the urate monoanion, leading to quenching of the Eu excited state. Such a transient bimolecular association process is entropically unfavourable; higher temperatures disfavour exciplex formation leading to the observed emission intensity enhancement. In contrast, the emission intensity decreased by 6% and 13% at 45 °C with ascorbate and iodide, respectively, and by 7.5% in the control with no added quencher. With iodide at least, a thermally activated collisional quenching mechanism is operating.

**Fig. 6:**
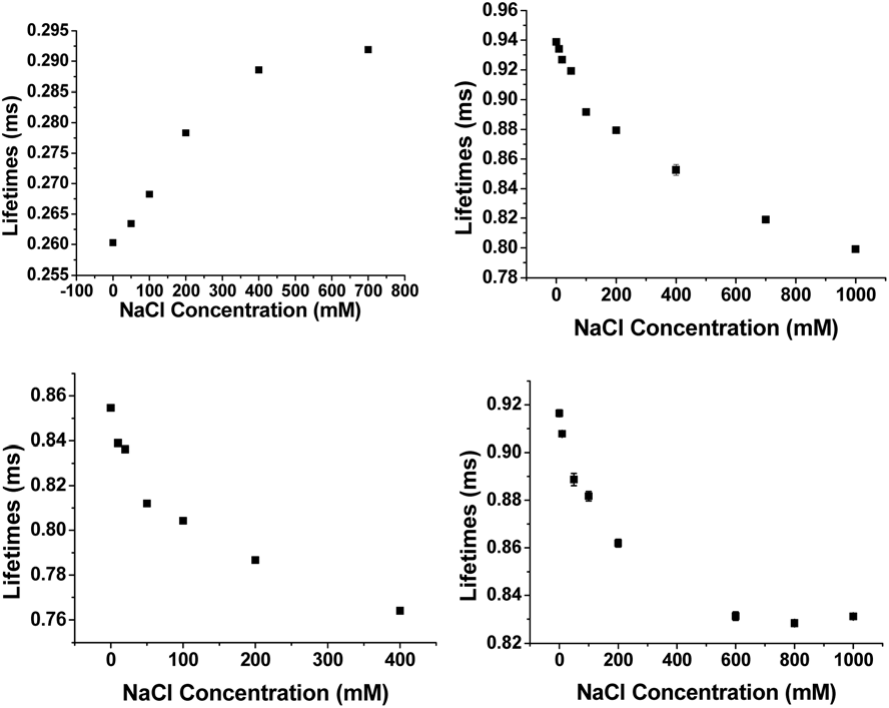
Ionic strength dependence of the Eu emission lifetime variation for [EuL^3^]^+^ (16 μM) in the presence of: (upper left) 50 μM urate; (upper right) 5 mM iodide; (lower left) 200 μM ascorbate; (lower right) control experiment, with no added quencher (pH 7.4, 0.1 M HEPES).

Experiments were also undertaken to explore the sensitivity of the emission lifetime of [EuL^3^]^+^ to variation of solution ionic strength, in the presence of a fixed concentration of different quenchers (Fig. 6). In the presence of 50 μM urate, the lifetime increased by 12% from 0.26 ms to 0.29 ms and reached a limit at 800 mM NaCl. In contrast, the lifetimes decreased by 15% and 11% in the presence of iodide (5 mM) and ascorbate (200 μM), respectively. Very similar behaviour was found in their emission intensity variations. In a control experiment, without added quencher, the Eu lifetime decreased from 0.92 ms to 0.83 ms over the studied salt range, reducing the significance of the data with added ascorbate and iodide, whilst emphasising the significance of the behaviour with urate.

The increase in ionic strength disfavours quenching owing to a classical kinetic salt effect. The urate monoanion and the lanthanide complex cation are increasingly surrounded by greater concentrations of ions of opposite charge; such conditions discourage exciplex formation. In the parallel studies with a fixed concentration of added iodide (5 mM) or ascorbate (200 μM), increasing the ionic strength led to an emission intensity decrease, contrasting with the behaviour with added urate, and mirroring the behaviour in the absence of any added quencher.

Parallel observations concerning the temperature and ionic strength were made in the Tb series of complexes, and are detailed in the ESI.†

### Preliminary cell microscopy experiments with 375 nm excitation

The availability of confocal microscopy instrumentation equipped with a 375 (or 370) nm laser excitation source has revitalised interest in the use of lanthanide complexes as luminescent stains and probes of the cellular environment. The complexes [EuL^2^] (*ϕ*_em_ 18%, *K*_SV_^−1^ (urate) 86 μM), [EuL^4^]^+^ (*ϕ*_em_ 30%, *K*_SV_^−1^ (urate) 23 μM) and [EuL^6^]^3+^ (*ϕ*_em_ 11%, *K*_SV_^−1^ (urate) 50 μM) each possess a dpqPh_2_ chromophore and were examined in preliminary comparative cell imaging experiments, using a Nikon AXR confocal microscope. Two cell lines were used: NIH-3T3 (mouse skin fibroblasts) and A549 (lung carcinoma) cells and the localisation profile of each complex was examined over 4 to 20 h incubation periods. At the outset, it was noted that these three complexes *in vitro* have different intrinsic brightnesses and a differing sensitivity to urate quenching. In the presence of excess protein *in vitro* using HSA as a model: log *K* values (298 K, pH 7.4, HEPES 0.1 M) were 4.98, 5.41 and 5.51 respectively, their emission was quenched by 43, 54 and 57% respectively. Furthermore, when urate (at the *K*_SV_^−1^ value) was added to 5 μM solutions of the complex in the presence of 100 μM HSA, the further reduction in overall Eu emission intensity was: [EuL^2^] (30%); [EuL^4^]^+^ (40%); [EuL^6^]^3+^ (12%).

**Fig. 7:**
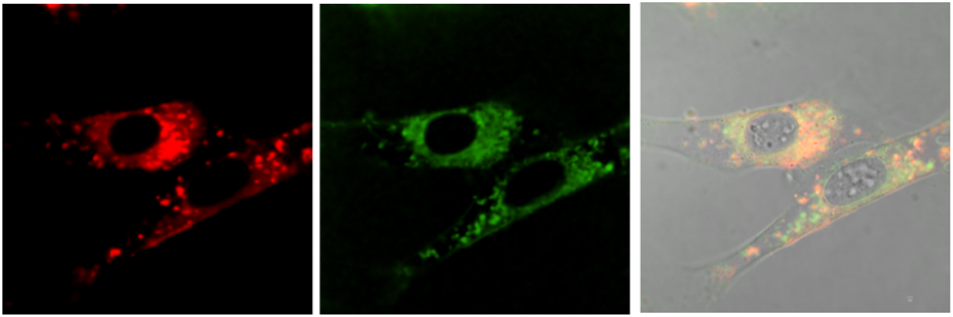
Confocal microscopy images of NIH-3T3 cells (295 K, *λ*_exc_ 375 nm) following incubation of [EuL^6^]^3+^ (20 μM, 6 h): (left) Eu (*λ*_em_ 580–630 nm); (centre) LysoTracker Green (*λ*_exc_ 375 nm, *λ*_em_ 510–540 nm); (right) merged with the brightfield image, *P* = 0.73.

No well-defined cell staining could be observed with [EuL^2^] over the given time periods with a 20 μM concentration of the complex in the incubation medium. Bright images were observed however, under these conditions with each of the cationic complexes. Brightest of all were images obtained with [EuL^6^]^3+^ and in NIH-3T3 cells, co-localisation experiments with MitoTracker-Green, LysoTracker-Green and ER-Tracker indicated that the complex was predominantly found in the perinuclear lysosomes at 6 h (*P* = 0.73 *vs.* 0.53 and 0.31 for MitoTracker and ER-Tracker respectively) and this profile did not change significantly with time (Fig. 7), although the images became even brighter. In contrast, in the 3T3 cell line, [EuL^4^]^+^ was only observable at 4 h, and showed a punctate distribution with no staining of the nucleus or nucleoli. The high brightness allowed spectral imaging experiments to be undertaken, and the fingerprint Eu emission signature could be observed with [EuL^6^]^3+^ in this cell line at 16 h, albeit at modest resolution, confirming the integrity of the coordination complex *in situ*.

**Fig. 8:**
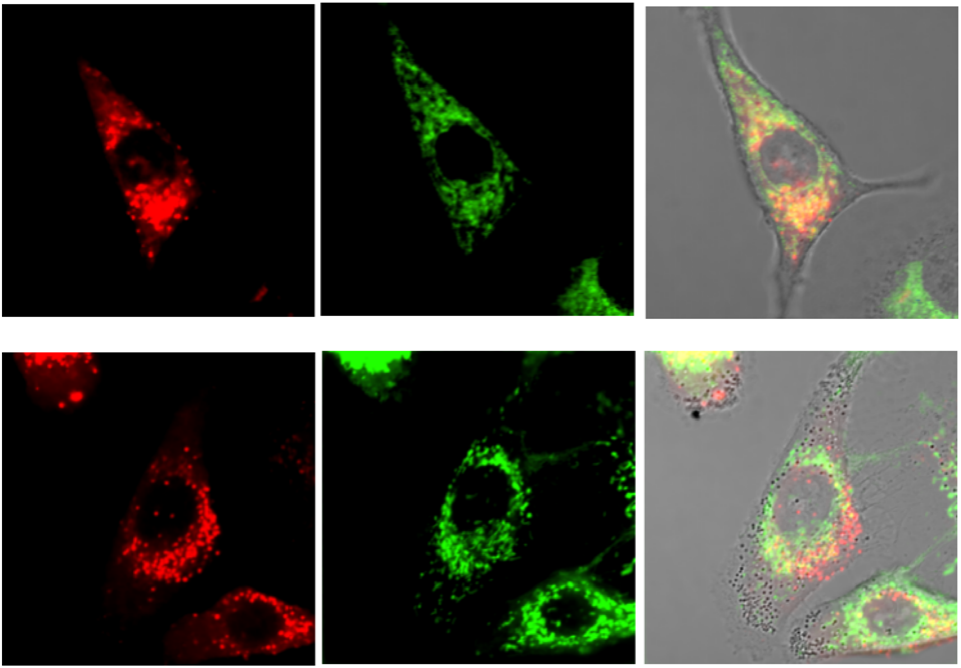
(upper) Confocal microscopy images of A549 cells (295 K, *λ*_exc_ 375 nm) following incubation of [EuL^4^]^+^ (20 μM, 16 h): (left) Eu (*λ*_em_ 580–630 nm); (centre) LysoTracker Green (510–540 nm); (right) merged with the brightfield image, *P* = 0.55. (lower): (left) Eu (580–630 nm); (centre) MitoTracker Green (*λ*_exc_ 488 nm, *λ*_em_ 510–540 nm); (right) merged with the brightfield image, *P* = 0.48.

In the A549 cancer cell line, both [EuL^4^]^+^ and [EuL^6^]^3+^ gave bright images at all time points (Fig. 8), and at 16 h a rather ‘spotty’ profile was observed and co-localisation experiments suggested a mixed distribution between the lysosomes and mitochondria, in each case (*P* = 0.55 and 0.48).

## Conclusions

A series of six new ligands has been prepared containing methyl or phenyl substituted tetra-azatriphenylene sensitising groups in macrocyclic ligands based on 1,4,7,10-tetra-azacyclododecane (cyclen) and 1,4,7-triazacyclononane (tacn). Selected rare earth coordination complexes have been prepared, in which the lanthanide ion is coordinated to each of the nine ligand donors with the exclusion of water, and overall emission quantum yields in solution were of the order of 10 to 50%. The S–T energy gap is small for the diphenyl series of complexes, and ligand fluorescence occurs with the Gd and Tb systems. The Eu analogue, simply showed strong metal-based emission at room temperature with no measurable ligand luminescence, as did each of the Eu and Tb complexes in the dimethyl series, consistent with fast rates of unidirectional inter-system crossing and efficient ligand to lanthanide ion energy transfer.

The excited state dynamics of the Eu, Gd and Tb complexes of [LnL^2^] were probed by transient absorption spectroscopy, characterising the decay kinetics of the intermediate ligand singlet and triplet excited states. The rates of decay of the singlet (in effect relating to the rate of intersystem S–T crossing) excited state were the same for the Gd and Tb complexes but were significantly faster for [EuL^2^]. Such behaviour aligns with recent observations for related series of complexes based on cyclen, in which there is an aryl-alkynyl sensitising moiety with internal charge transfer (ICT), rather than ligand localized, singlet and triplet excited states.^22,23^ In those cases, the rate of ISC was up to twenty times faster for the Eu analogue compared to Gd. Such unique behaviour for Eu complexes merits further scrutiny, examining a wider range of lanthanide complexes across the series, with electron poor and electron rich sensitising groups. It may relate to the facility of electron transfer to the Eu ion, owing to the relative ease of reduction to the Eu^2+^ state, facilitating fast resonant spin–orbit coupling.

The highest sensitivity to quenching by urate was observed with the mono-cationic complex [LnL^3^]^+^ (Ln = Eu/Tb) and it was used directly for the ratiometric analysis of diluted serum, aided by the seven-fold difference of Tb/Eu quenching by urate. This system has the advantage of being readily functionalised *via* the 4-position of the pyridine ring,^30,31^ permitting conjugation to different substrates or biomolecules for *in situ* urate analysis. The temperature and ionic strength dependence of the lifetime and intensity of Eu and Tb emission strongly support the hypothesis that urate quenching involves the intermediacy of an exciplex, formed between the urate anion and the dpq triplet excited state.

Confocal microscopy studies showed how the dpqPh_2_ sensitising moiety is well suited to imaging of Eu(iii) complexes, following 375 nm laser excitation. The pattern of behaviour amongst the complexes suggests that the extent of urate quenching of these probes *in cellulo* (either when the complex is ‘free’ or protein bound) is an important consideration in assessing probe suitability for imaging. Indeed, urate is ubiquitous in all cells. Thus, [EuL^6^]^3+^ a complex that showed least sensitivity *in vitro* to urate quenching when protein bound, gave rise to the brightest images *in cellulo*, as deduced by confocal microscopy. The absence of sensitisation at room temperature of the Tb analogues with the dpqPh_2_ chromophore, however, currently inhibits the use of Eu/Tb ratiometric analysis in microscopy for these systems.

## Data availability

The data supporting this article have been included as part of the ESI.†

## Author contributions

The manuscript was written by DP with contributions from each author; XW carried out the syntheses and characterisation, the measurements of binding and rate constants at steady state; H. Li performed the cell imaging experiments; ZJ undertook the transient absorption studies under direction from RD.

## Conflicts of interest

There are no conflicts to declare.

## Supplementary Material

SC-OLF-D4SC05743K-s001
