# Supplementary material for: Mechanism of action and evaluation of ratiometric probes for uric acid using lanthanide complexes with tetraazatriphenylene sensitisers
Source: Chem Sci. 2024 Nov 7;15(47):19944–51. doi: 10.1039/d4sc05743k (PMC11575574; doi:10.1039/d4sc05743k)
Supplement: SC-015-D4SC05743K-s001 [file SC-015-D4SC05743K-s001.pdf]

Supplementary Information (SI) for Chemical Science.
This journal is © The Royal Society of Chemistry 2024

Mechanism of Action and Evaluation of Ratiometric Probes for Uric Acid Using Lanthanide Complexes with Tetraazatriphenylene Sensitisers

Xinyi Wen [a] Zhijie Ju [b] Huishan Li [a] Renren Deng [b] and David Parker [a*]

a) Department of Chemistry, Hong Kong Baptist University, Kowloon Tong, 999077, Hong Kong, China

b) State Key Laboratory of Silicon and Advanced Semiconductor Materials, Institute for Composites Science Innovation, School of Materials Science and Engineering, Zhejiang University, Hangzhou 310058, China

1. General Procedures; HPLC, optical techniques, live cell imaging, co-localisation studies and analysis of urate in diluted human serum. pages 1-4

2. Synthetic Procedures pages 5-27

3. Photoluminescence and transient absorption data and $[TbL^3]^+$ quenching sensitivity to T and I pages 28-37

4. 13-C and 1-H NMR spectra pages 38-51

5. References pages 52-54

Experimental

General procedures

All reagents were used as received from their respective suppliers. Solvents were laboratory grade and were dried over appropriate drying agents when necessary. Air sensitive reactions were carried out under an atmosphere of nitrogen. Thin-layer chromatography was carried out on silica plates and visualised under UV irradiation (254/365 nm). Preparative column chromatography was performed using Biotage® Isolera™ One equipped with 200-800 nm UV-Vis detector and Biotage® Rening Cartridge – High Performance 40-63 µm. Crudes were loaded on silica gel (Bidepharm, 100-200 mesh). Melting points were recorded using a Cole-Parmer® MP-250D-F apparatus and are uncorrected.

HPLC analysis

HPLC analyses and purifications were performed at 295 K with three different set-ups. All chromatograms report monitoring the absorbance at 254 nm.

Agilent system. Agilent 1100 module HPLC system (Agilent Technologies, Stockport, UK), G1313A Autosampler (Micro-WPS), G1312A Binary Pump, G1315A Diode-Array Detector (DAD) and Agilent 5 HC-C18 (2) column (5 µm, 4.6 x 250 mm).

Waters system. Waters 2707 Autosampler, Water 1525 Binary HPLC Pump, Waters 2998 Photodiode Array Detector and Waters Fraction Collector III and Atlantis® T3 Prep OBD™ C18 column (5 µm, 19 × 250 mm).

Shimadzu system. Semi-preparative High Performance Liquid Chromatograph LC-20AR, LC-20AR Solvent Delivery Pump, DGU-40 Degassing unit, LH-40 Liquid Handler, SPD-M40 Photodiode Array Detector, FRC-40 Fraction Collector, CBM-40 System Controller and XBridge® Prep C18 OBD™ column (5 µm, 19 × 100 mm).

Various chromatographic systems were employed for analytical and preparative HPLC:

Method A: (Agilent system) flow rate 0.5 mL/min with H_2O (0.1% TFA) – 20% MeCN (0.1% TFA) as eluents (linear gradient to 80% MeCN (0.1% TFA) [40 min].
Method B: (Agilent system) flow rate 1.0 mL/min with H_2O (0.1% TFA) – 30% MeCN (0.1% TFA) as eluents (gradient to 100% MeCN (0.1% TFA) [20 min].
Method C: (Waters system) flow rate 5.0 mL/min with H_2O (0.1% TFA) – 20% MeCN (0.1% TFA) as eluents (gradient to 59% MeCN (0.1% TFA) [26 min].
Method D: (Waters system) flow rate 5.0 mL/min with H_2O (0.1% TFA) – 20%

MeCN (0.1% TFA) as eluents (gradient to 71% MeCN (0.1% TFA) [34 min].
Method E: (Waters system) flow rate 5.0 mL/min with H_2O (0.1% TFA) – 30% MeCN (0.1% TFA) as eluents (gradient to 50% MeCN (0.1% TFA) [20 min].
Method F: (Waters system) flow rate 5.0 mL/min with H_2O (0.1% TFA) – 30% MeCN (0.1% TFA) as eluents (gradient to 60% MeCN (0.1% TFA) [24 min].
Method G: (Waters system) flow rate 5.0 mL/min with H_2O (0.1% TFA) – 30% MeCN (0.1% TFA) as eluents (gradient to 66% MeCN (0.1% TFA) [34 min].
Method H: (Waters system) flow rate 5.0 mL/min with H_2O (0.1% TFA) – 30% MeCN (0.1% TFA) as eluents (gradient to 81% MeCN (0.1% TFA) [34 min].
Method I: (Shimadzu system) flow rate 17.0 mL/min with H_2O (0.1% TFA) – 10% MeCN (0.1% TFA) as eluents (gradient to 100% MeCN (0.1% TFA) [14 min].
Method J: (Shimadzu system) flow rate 5.0 mL/min with H_2O (0.1% TFA) – 20% MeCN (0.1% TFA) as eluents (gradient to 47% MeCN (0.1% TFA) [27 min].
Method K: (Shimadzu system) flow rate 5.0 mL/min with H_2O (0.1% TFA) – 30% MeCN (0.1% TFA) as eluents (gradient to 66% MeCN (0.1% TFA) [24 min].
Method L: (Shimadzu system) flow rate 5.0 mL/min with H_2O (0.1% TFA) – 20% MeCN (0.1% TFA) as eluents (gradient to 56% MeCN (0.1% TFA) [24 min].
Method M: (Shimadzu system) flow rate 5.0 mL/min with H_2O (0.1% TFA) – 30% MeCN (0.1% TFA) as eluents (linear gradient to 75% MeCN (0.1% TFA) [30 min].

Optical techniques

UV/Vis absorbance spectra were recorded using an Agilent Technologies Cary 8454 UV-Vis spectrometer. Emission spectra were recorded on a HORIBA Scientific Fluoromax-4 luminescence spectrofluorimeter. Lifetime measurements were carried out using a HORIBA Fluorolog-3 spectrometer under the stated conditions. Temperature dependent measurements were carried out using Peltier-controlled cuvette holder supplied with magnetic stirring using T-App software.

The picosecond transient absorption (psTA) experiments were conducted using pump-probe technology. The excitation wavelength was 343 nm. The pump light is generated from an optical parametric amplifier (OPA) excited by a 1030 nm femtosecond laser (Pharos, Light Conversion). The probe light was generated by focusing 1030 nm laser onto a YAG crystal after passing through an automatic delay stage with a >1 ns time range. The nanosecond transient absorption (nsTA) experiments used a commercial system (LP920, Edinburgh). The excitation wavelength was 400 nm. All TA measurements were obtained at room temperature and employed a reference probe light to eliminate the laser fluctuations.

Live cell imaging and colocalization studies

NIH3T3 cells and A 549 cells were cultured in Dulbecco's modified Eagle's medium (DMEM) (Thermo Fisher Scientific Inc, USA) supplemented with foetal bovine serum (10 %), and penicillin/streptomycin (1 %). The cells were incubated at 37 °C at 5% CO_2 and 10% humidity. The cells were allowed to

grow to 70-80% confluence, when the medium was removed and replaced with medium containing complex. Cell imaging was undertaken using a Nikon AXR confocal microscope equipped with a 375 nm laser excitation source.
For co-localisation studies, MitoTracker Green (200 nM) and ER-Tracker Green(1 μM) were added to th e medium 15 min before imaging, while LysoTracker Green (75 nM) was added 20 min before imaging. Before imaging, the coverslips were washed with PBS buffer three times. In each case, the following experimental parameters were used for imaging. For europium complexes: λ_{exc} = 375 nm, λ_{em} 580-720 nm; for MitoTracker Green, LysoTracker Green, and ER-Tracker Green: λ_{exc} = 488 nm, λ_{em} 500-530 nm.

Determination of the calibration curve in diluted human serum

In preparing for the assay, three solutions were made:

1) A stock solution of pH adjusted buffer (pH 7.4 0.1M HEPES) was used in preparing human serum stock solution and subsequent dilution.
2) A 1:1 mixture of [EuL^3] and [TbL^3] (each typically giving rise to a solution that was 30 μM concentration in each complex) with an absorbance in a buffered aqueous solution of 0.1.
3) A stock solution of sodium urate in pH adjusted buffer (solution was standardised measuring absorbance at 290 nm, ε = 1.22 x 10^4 M^{-1} cm^{-1}).

The complexes were dissolved separately in buffer solution and their concentrations adjusted to give absorbances of 0.1. The two solutions of equal absorbance were combined in equal volumes.The lyophilized human serum (from 1 mL) was dissolved in 2 mL pH 7.4 0.1 M HEPES buffer and diluted 50-fold.

In order to determine the calibration curve, 500 μL of diluted human serum solution was added to a 1 mL cuvette followed by 500 μL of the complex solution (250 μL [EuL^3] + 250 μL [TbL^3]) and incremental additions of sodium urate were made. The emission intensity ration was recorded, each measurement being performed in triplicate. The calibration was undertaken by plotting the 540/614 nm(green/red) luminescence intensity ratio *vs* urate concentration, fitting the experimental curve to a bi-exponential model.

Synthetic Procedures

2-Methyl-2',3'-dimethylpyrazino-1,10-phenanthroline (Scheme 1)

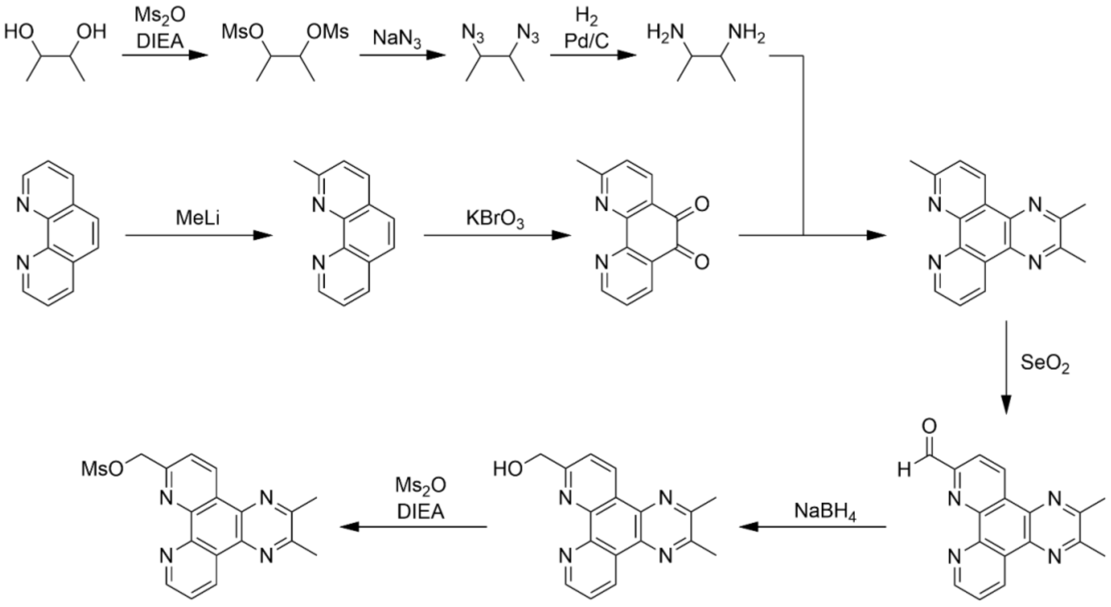

2-Methyl-1,10-phenanthroline, was prepared as described previously. [1] **2-Methyl-1,10-phenanthroline-5,6-dione** was prepared using a modified procedure and has been previously reported; one batch was prepared by the company AtlanChim in France.

Meso-2,3-butanediol dimesylate, 1 [2]

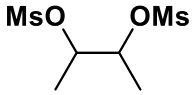

To a solution of *meso*-2,3-butanediol (1.2 g, 13.3 mmol) in anhydrous THF (40 mL) was added DIEA (9.5 mL, 54.5 mmol) and methanesulfonic anhydride (7 g, 40.2 mmol) at 0 ºC. The solution was stirred for 4 h and allowed to reach room temperature. After this time, the reaction mixture was poured portion-wise onto crushed ice with vigorous stirring for 30 min. The mixture was left to stand for 1 h, before filtering to isolate a cream solid. The solid was washed liberally with cold water and dried under high vacuum to afford a white solid (1.9 g, 59 %). **^{1}H NMR** ($CDCl_3$, 400 MHz, 295 K) 4.94–4.85 (2H, m, -C<u>H</u>CH_3), 3.08 (6H, s, -SO_3C<u>H</u>$_3$), 1.44 (6H, d, $^3J_{H-H}$ 6.4, -CHC<u>H</u>$_3$); **^{13}C NMR** ($CDCl_3$, 101 MHz, 295 K)

δ 79.1 (-$\underline{C}HCH_3$), 38.9 (-$SO_3\underline{C}H_3$), 16.1 (-$CH\underline{C}H_3$).

2,3-Diazidobutane, 2 [3]

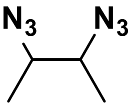

The dimesylate **1** (1 g, 4.06 mmol) and NaN_3 (2.1 g, 32.3 mmol) were combined in anhydrous DMF (15 mL) and the solution was heated to 80 °C under argon for 21 h. After this time, the reaction was allowed to cool, 10% NaCl solution (40 mL) was added, and the aqueous solution was extracted with diethyl ether (6 × 50 mL). The combined organic layers were dried over Na_2SO_4 and the solvent removed under reduced pressure to afford a pale yellow oil containing the product and residual DMF. This oil was used in the next step *without further purification*.

2,3-Butanediamine, 3 [4]

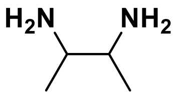

The oil containing the diazide **2** was dissolved in absolute ethanol (50 mL) to which palladium on carbon was added (Pd content 10 %, 20 mg). The vessel was then loaded onto a Parr hydrogenator (pressure 40 mbar H_2) and the reaction mixture was agitated for 24 h. After this time, the catalyst was removed by filtration and the volume of the solution was reduced under reduced pressure for direct use in the next step.

2-Methyl-5,6 -dimethyl-dipyridoquinoxaline, 4

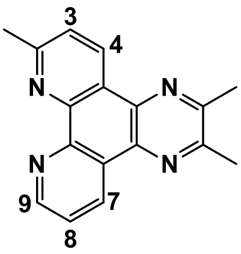

To the *crude solution* of the diamine **3** in ethanol (20 mL) was added 2-methyl-1,10-phenanthroline-5,6-dione (500 mg, 2.23 mmol) and the subsequent yellow solution was heated at 75 °C for 17 h. After this time, the solvent was removed

under reduced pressure. The resulting residue was recrystallised from hot ethanol to afford fine yellow crystals (344 mg, 56 %); **^{1}H NMR** ($CDCl_3$, 400 MHz, 295 K) 9.44 (1H, dd, $^3J_{H-H}$ 8.0, $^4J_{H-H}$ 1.5, H^9), 9.31 (1H, d, $^3J_{H-H}$ 8.5, H^4), 9.20 (1H, dd, $^3J_{H-H}$ 8.0, $^4J_{H-H}$ 1.5, H^7), 7.72 (1H, dd, $^3J_{H-H}$ 8.0, H^8), 7.60 (1H, d, $^3J_{H-H}$ 8.5, H^3), 2.93 (3H, s, C$\underline{H}_3$), 2.79 (6H, s, C$\underline{H}_3$); **^{13}C NMR** ($CDCl_3$, 101 MHz, 295 K) 161.0 (q Ar), 153.3 (q Ar), 152.9 (q Ar), 151.4 (C^7), 146.7 (q Ar), 146.3 (q Ar), 137.6 (q Ar), 137.0 (q Ar), 133.1 (C^9), 133.0 (C^4), 127.2 (q Ar), 124.9 (q Ar), 124.4 (C^8), 123.6 (C^3), 25.5 ($\underline{C}H_3$), 22.9 ($\underline{C}H_3$), 22.9 ($\underline{C}H_3$); **Melting point**: 243-246˚C; **ESI-LRMS** (+) *m/z* 275 [M+H]$^+$; **ESI-HRMS** (+) calcd $[C_{17}H_{15}N_4]^+$ 275.1297, found 275.1293.

5,6 -Dimethyl-dipyridoquinoxaline-2-carbaldehyde, 5

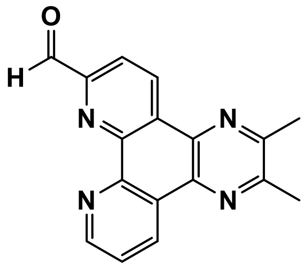

A solution of compound **4** (220 mg, 0.80 mmol) in dioxane (50 mL) was prepared. To this solution was added SeO_2 (190 mg, 1.71 mmol) and the solution was heated to reflux with reaction monitoring by TLC every 30 min (SiO_2, DCM). After 4 h, no further change was observed by TLC and the reaction was cooled, filtered, and the solvent removed under reduced pressure to afford a crude residue. This material was used in the next step without further purification; **ESI-LRMS** (+) *m/z* 289 [M+H]$^+$; **ESI-HRMS** (+) calcd $[C_{17}H_{13}N_4O]^+$ 289.1089, found 289.1074.

2-Hydroxylmethyl -5,6 -dimethyl-dipyridoquinoxaline, 6

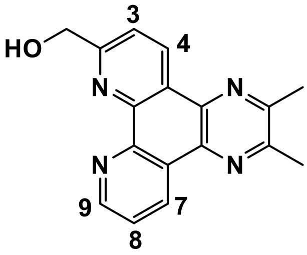

The crude aldehyde **5** was dissolved in DCM/ethanol (1:1, 100 mL) and $NaBH_4$ (31 mg, 0.819 mmol, ~ 1 eq.) was added. The mixture was heated to reflux for 2 h, before removal of the solvents under reduced pressure. The subsequent residue was dissolved in water (40 mL), extracted with DCM (5 × 40 mL), and dried over K_2CO_3. Removal of the solvent under reduced pressure afforded a dark yellow solid (180 mg, 77% over two steps); **^{1}H NMR** ($CDCl_3$, 600 MHz, 295 K) 9.41 (1H, dd, $^3J_{H-H}$ 8.0, $^4J_{H-H}$ 2.0, H^9), 9.29 (1H, d, $^3J_{H-H}$ 8.0, H^4), 9.11 (1H, dd, $^3J_{H-H}$ 8.0, $^4J_{H-H}$ 2.0, H^7), 7.74 (1H, dd, $^3J_{H-H}$ 8.0, H^8), 7.63 (1H, d, $^3J_{H-H}$ 8.0, H^3), 6.01 (1H, br. s, O<u>H</u>), 5.06 (2H, s, C<u>H</u>$_2$OH), 2.77 (3H, s, C<u>H</u>$_3$), 2.77 (3H, s, C<u>H</u>$_3$); **^{13}C NMR** ($CDCl_3$ & MeOH, 151 MHz, 295 K) 162.1 (q Ar), 153.6 (q Ar), 153.2 (q Ar), 150.8 (C^7), 146.3 (q Ar), 145.5 (q Ar), 137.3 (q Ar), 136.8 (q Ar), 133.4 (C^9), 133.4 (C^4), 127.4 (q Ar), 125.9 (q Ar), 123.9 (C^8), 121.3 (C^3), 65.6 (<u>C</u>H$_2$OH), 22.9 (<u>C</u>H$_3$), 22.9 (<u>C</u>H$_3$); **Melting point**: greater than 400 ˚C; **ESI-LRMS** (+) *m/z* 291 [M+H]$^+$; **ESI-HRMS** (+) calcd $[C_{17}H_{15}N_4O]^+$ 291.1246, found 291.1219.

2-Methanesulfonatomethyl -5,6 -dimethyl-dipyridoquinoxaline, 7

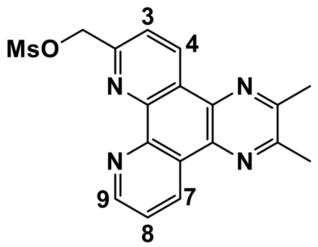

The alcohol **6** (50 mg, 0.172 mmol), methanesulfonic anhydride (240 mg, 1.38 mmol) and DIEA (3.2 mL, 18.4 mmol) were combined under argon in anhydrous THF (5 mL). The reaction was stirred at room temperature for 6 h, before removal of the solvent under reduced pressure. The resultant residue was dissolved in DCM and washed with water (3 × 20 mL). The aqueous layers with extracted with DCM (1 × 20 mL) before the combined organic layers were dried over Na_2SO_4 and the solvent removed under reduced pressure to afford a pale yellow solid (63 mg, quant.); **^{1}H NMR** ($CDCl_3$, 400 MHz, 295 K) 9.53 (1H, d, $^3J_{H-H}$ 8.5, H^4), 9.48 (1H, dd, $^3J_{H-H}$ 8.0, $^4J_{H-H}$ 2.0, H^9), 9.24 (1H, dd, $^3J_{H-H}$ 8.0, $^4J_{H-H}$ 2.0, H^7), 7.97 (1H, d, $^3J_{H-H}$ 8.5, H^3), 7.76 (1H, dd, $^3J_{H-H}$ 8.0, H^8), 5.78 (2H, s, C<u>H</u>$_2$OMs), 3.21 (3H, s, -OSO$_2$C<u>H</u>$_3$), 2.82 (6H, s, C<u>H</u>$_3$); **ESI-LRMS** (+) *m/z* 369 [M+H]$^+$; **ESI-HRMS** (+) calcd $[C_{18}H_{17}N_4O_3S]^+$ 369.1021, found 369.1026.

L^1 (DO3A-dpQMe$_2$) (Scheme 2)

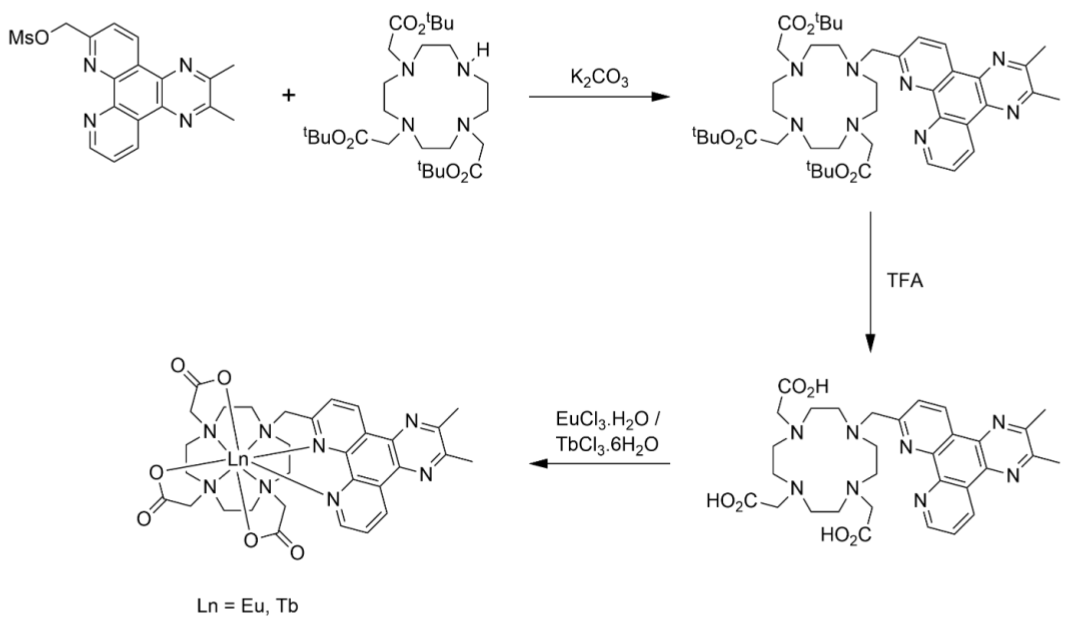

Tris(t-butyl) ester of L[1], 8

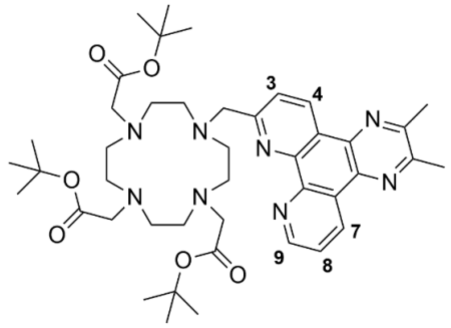

To a mixture of the tri-t-butyl ester (86 mg, 0.17 mmol) and K_2CO_3 (48 mg, 0.35 mmol) in anhydrous acetonitrile (5 mL) under argon was added the mesylate **7** (63 mg, 0.17 mmol). The mixture was heated at 60 °C for 18 h before being allowed to cool. The reaction solution was separated from the inorganic salts by filtration and purified by RP-HPLC (*Method I*, t_R = 13.2 min) to afford a brown residue (41 mg, 30 %); **^{1}H NMR** ($CDCl_3$, 600 MHz, 295 K) 9.45 (1H, dd, $^3J_{H-H}$ 8.0, $^4J_{H-H}$ 2.0, H^9), 9.42 (1H, d, $^3J_{H-H}$ 8.5, H^4), 9.24 (1H, dd, $^3J_{H-H}$ 8.0, $^4J_{H-H}$ 2.0, H^7), 8.39 (1H, d, $^3J_{H-H}$ 8.5, H^3), 7.70 (1H, dd, $^3J_{H-H}$ 8.0, H^8), 4.17 (2H, s, C$\underline{H}_2$Ar), 3.38 (2H, s, C$\underline{H}_2CO_2{}^tBu$), 3.21 (4H, s, C$\underline{H}_2CO_2{}^tBu$), 2.97 – 2.84 (12H, m, 12-N_4), 2.80 (3H, s, C$\underline{H}_3$), 2.80 (3H, s, C$\underline{H}_3$), 2.78 – 2.75 (4H, m, 12-N_4), 1.47 (9H, s, $CO_2C(C\underline{H}_3)_3$, 1.37 (18H, s, $CO_2C(C\underline{H}_3)_3$; **^{13}C NMR** ($CDCl_3$, 151 MHz, 295 K) 171.4 ($\underline{C}O_2{}^tBu$), 171.2 ($\underline{C}O_2{}^tBu$), 163.9 (q Ar), 153.2 (q Ar), 152.7 (q Ar), 151.6 (C^7), 147.1 (q Ar), 146.2 (q Ar), 137.8 (q Ar), 137.1 (q Ar), 133.3 (C^4), 132.9 (C^9), 127.2 (q Ar), 125.9 (q Ar), 123.8 (C^3), 123.4 (C^8), 80.8 ($CO_2\underline{C}(CH_3)_3$), 80.8 ($CO_2\underline{C}(CH_3)_3$), 62.6 ($\underline{C}H_2$Ar), 56.8 ($\underline{C}H_2CO_2{}^tBu$), 56.7 ($\underline{C}H_2CO_2{}^tBu$), 53.4 (12-$N_4$), 52.8 (12-$N_4$), 52.4 (12-$N_4$), 52.3 (12-$N_4$), 28.4 ($CO_2C(\underline{C}H_3)_3$), 28.3 ($CO_2C(\underline{C}H_3)_3$), 22.9 ($\underline{C}H_3$); **ESI-LRMS** (+) *m/z* 787 $[M+H]^+$; **ESI-HRMS** (+)

calcd $[C_{43}H_{63}N_8O_6]^+$ 787.4871, found 787.4884.

Ligand $[H_6L^1]^{3+}$, 9 (as its triprotonated TFA salt)

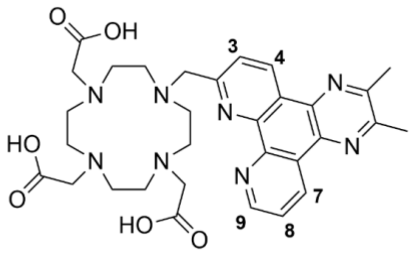

A solution of the *t*-butyl ester (40 mg, 0.051 mmol) in TFA/DCM (1:1) was prepared and stirred at room temperature for 2 h. After this time the solvent was removed under reduced pressure. The resulting residue was then re-dissolved in DCM before removal of this solvent under reduced pressure. This process was repeated 4 times. The residue was then dried under high vacuum to afford the protonated ligand as a pale orange residue (30 mg, quant.); **^{1}H NMR** ($CDCl_3$, 600 MHz, 295 K) 9.83 (1H, d, $^3J_{H-H}$ 8.0, H^9), 9.43 – 9.28 (2H, m, H^4 & H^7), 8.41 – 8.31 (1H, m, H^8), 8.12 (1H, d, $^3J_{H-H}$ 8.2, H^3), 3.62 (2H, s, C$\underline{H}_2$Ar), 3.53 – 2.99 (22H, m, 12-N_4, C$\underline{H}_2CO_2{}^tBu$), 2.83 (6H, s, C$\underline{H}_3$); **ESI-LRMS** (+) *m/z* 619 $[M+H]^+$; **ESI-HRMS** (+) calcd $[C_{31}H_{39}N_8O_6]^+$ 619.2993, found 619.3004.

$[LnL^1]$

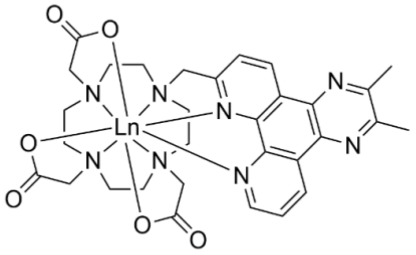

The ligand L^1 in its protonated form (1 eq.) was dissolved in a methanolic solution (3:1 methanol/water) and the pH was adjusted to 6.5. To this solution was added $LnCl_3.6H_2O$ (1.1 eq.) followed by heating at 60 °C for 15 h. After this

time the solution was separated from the inorganic salts by filtration and the solvent removed under reduced pressure to afford the complex.

[EuL[1]]

The europium complex was isolated as a yellow residue, and was purified via reverse-phase HPLC (*Method I*, t_R = 1.3 min) to yield a white solid (23 mg, 90 %); **ESI-LRMS** (+) *m/z* 767 [M+H]$^+$; **ESI-HRMS** (+) calc. $[C_{31}H_{35}{}^{151}EuN_8O_6]^+$ 767.1956, found 767.1942; emission lifetime τ_{em} = (1.09 ± 0.01) ms; λ_{exc} = 345 nm.

[TbL[1]]

The terbium complex was isolated as a white residue, and was purified via reverse-phase HPLC (*Method I*, t_R = 1.4 min) to yield a white solid (8 mg, 90 %); **ESI-LRMS** (+) *m/z* 775 [M+H]$^+$; **ESI-HRMS** (+) calc. $[C_{31}H_{35}TbN_8O_6]^+$ 775.2011, found 775.2006; emission lifetime τ_{em} = (1.86 ± 0.01) ms; λ_{exc} = 345 nm.

L[2] (DO3A-dpQPh$_2$) (Scheme 3)

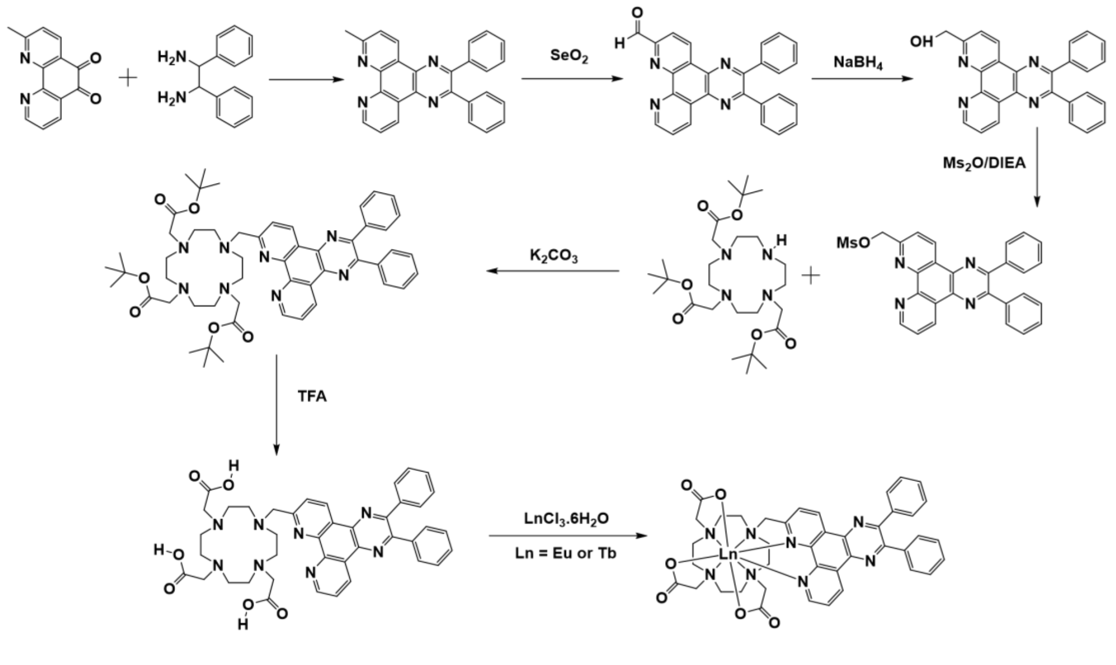

2-Methyl-5,6-diphenyl-dipyrridoquinoxaline, 10

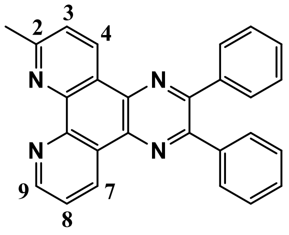

To a solution of *meso*-1,2-diphenylethane-1,2-diamine (0.127g, 0.6 mmol) in ethanol (5 mL) was added 2-methyl-1,10-phenanthroline-5,6-dione (0.067g, 0.3 mmol) and the yellow solution was heated at 75 ˚C for 17 h. After this time, the solvent was removed under reduced pressure, and the residue was purified by column chromatography (silica gel, DCM-MeOH 0-5% gradient elution). The title product was obtained as a pale-yellow solid (56.1 mg, 47 %), R_f (silica, DCM-MeOH, 90:10): 0.42; **^{1}H NMR** ($CDCl_3$, 400 MHz, 298 K) 9.58 (1H, dd, $^3J_{H-H}$ 8.0, $^4J_{H-H}$ 2.0, H^9), 9.46 (1H, d, $^3J_{H-H}$ 8.5, H^4), 9.31 (1H, dd, $^3J_{H-H}$ 4.5, $^4J_{H-H}$ 2.0, H^7), 7.77 (1H, dd, $^3J_{H-H}$ 8.0, $^4J_{H-H}$ 4.5, H^8), 7.72-7.64 (5H, m, Ar-H & H^3), 7.46-7.37 (6H, m, Ar-H), 3.01 (3H, s, CH_3); **^{13}C NMR** ($CDCl_3$, 101 MHz, 298 K) δ 161.8 (1C, q Ar, C^2), 152.7 (1C, q Ar), 152.3 (1C, q Ar), 152.3 (1C, C^7), 147.7 (1C, q Ar), 147.3 (1C, q Ar), 139.0 (1C, q Ar), 138.2 (1C, q Ar), 137.6 (1C, q Ar), 133.6 (1C, C^4), 133.5 (1C, C^9), 130.2 (2C, Ar), 130.2 (2C, Ar), 129.2 (2C, Ar), 129.2 (2C, Ar), 128.5 (2C, Ar), 127.4 (1C, q Ar), 127.2 (1C, q Ar), 125.0 (1C, q Ar), 124.6 (1C, C^3), 123.8 (1C, C^8), 26.0 (1C, $\underline{C}H_3$). **Melting point**: 283-285 ˚C. **ESI-HRMS** (+) calc. $[C_{27}H_{19}N_4]^+$ 399.1531, found 399.1540.**2-Methanoyl-5,6-diphenyl-dipyridoquinoxaline, 11**

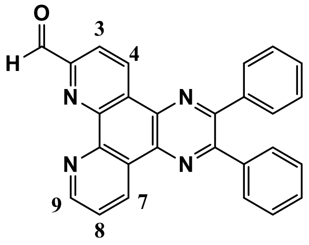

To a solution of 2-methyl-2',3'-diphenylpyrazino-1,10-phenanthroline (72 mg, 0.18 mmol) in dioxane (10 mL) was added SeO_2 powder (44 mg, 0.40 mmol) and the solution was heated at 105 ˚C. After 2 h, TLC analysis indicated complete consumption of the starting material and the reaction mixture was cooled, filtered, and solvent removed under reduced pressure to afford the aldehyde as a crude oily residue that was used in the next step without further purification. **^{1}H NMR** ($CDCl_3$, 400 MHz, 298 K) 10.62 (1H, s, $C\underline{H}O$), 9.77 (1H, d, $^3J_{H-H}$ 8.0, H^9), 9.63 (1H, dd, $^3J_{H-H}$ 8.0, $^4J_{H-H}$ 2.0, H^4), 9.37 (1H, dd, $^3J_{H-H}$ 4.5, $^4J_{H-H}$ 2.0, H^7), 8.44 (1H, d, $^3J_{H-H}$ 8.0, H^8), 7.87 (1H, dd, $^3J_{H-H}$ 8.0, $^4J_{H-H}$ 4.5, H^3), 7.73-7.68 (4H, m, Ar-H), 7.46-7.40 (6H, m, Ar-H); **^{13}C NMR** ($CDCl_3$, 101 MHz, 298 K) δ 194.0 (1C, $\underline{C}HO$), 153.8 (1C, q Ar), 153.4 (2C, q Ar), 152.6 (1C, C^7), 147.7 (1C, q Ar), 147.2 (1C, q Ar), 138.9 (1C, q Ar), 138.6 (2C, q Ar), 138.6 (1C,

q Ar), 137.2 (1C, qAr), 135.0 (1C, C^9), 133.8 (1C, C^4), 130.2 (2C, Ar), 130.2 (2C, Ar), 129.6 (2C, Ar), 129.6 (2C, Ar)128.6 (2C, Ar), 127.8 (1C, q Ar), 124.6 (1C, C^3), 120.8 (1C, C^8). **ESI-HRMS** (+) calc. $[C_{27}H_{17}N_4O]^+$ 413.1324, found 413.1340

2-Hydroxymethyl -7,8 -diphenyl-dipyridoquinoxaline, 12

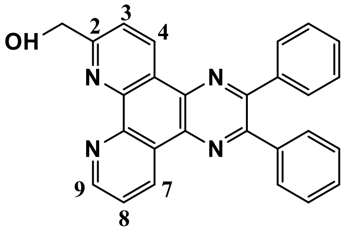

The crude aldehyde compound was dissolved in DCM/ethanol (1:1, 20 mL) and $NaBH_4$ (7 mg, 0.185 mmol) was added. The mixture was heated to reflux for 2 h, before the removal of the solvent under reduced pressure. The subsequent residue was dissolved in water (10 mL), extracted with DCM (5 × 10 mL), and the combined organic extracts were dried over K_2CO_3. After filtering, removal of the solvent under reduced pressure afforded a pale-yellow solid, R_f (silica, DCM-MeOH, 90:10): 0.31. **^{1}H NMR** ($CDCl_3$, 400 MHz, 298 K) 9.54 (1H, dd, $^3J_{H-H}$ 8.0, $^4J_{H-H}$ 2.0, H^9), 9.47 (1H, d, $^3J_{H-H}$ 8.5, H^4), 9.24 (1H, dd, $^3J_{H-H}$ 8.5, $^4J_{H-H}$ 2.0, H^7), 7.85 (1H, d, $^3J_{H-H}$ 8.5, H^8), 7.79 (1H, dd, $^3J_{H-H}$ 8.0, $^4J_{H-H}$ 2.0, H^3), 7.72-7.67 (4H, m, Ar-H), 7.45-7.38 (6H, m, Ar-H), 5.21(2H, d, $^3J_{H-H}$ 5.2, CH_2). **^{13}C NMR** (298 K, 101 MHz, $CDCl_3$) δ 163.0 (C^2), 152.9 (q Ar), 152.6 (q Ar), 151.6 (C^7), 147.2 (q Ar), 146.5 (q Ar), 138.8 (q Ar), 137.9 (q Ar), 137.5 (q Ar), 134.0 (C^4), 133.8 (C^9), 130.2 (Ar), 129.3 (Ar), 128.5 (Ar), 127.4 (q Ar), 126.0 (q Ar), 124.0 (C^8), 121.5 (C^3), 65.8 ($\underline{C}H_2$). **ESI-MS** *m/z* 415.601 $[M+H]^+$. **ESI-HRMS** (+) calc. $[C_{27}H_{19}N_4O]^+$ 415.1553, found 415.1544. **Melting point** (br. range): 334-361 ˚C.

2-Methanesulfanatomethyl -5,6 -diphenyl-dipyridoquinoxaline, 13

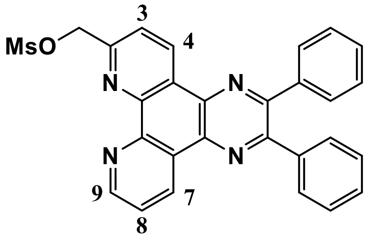

The alcohol **12** (50 mg, 0.12 mmol), methanesulfonic anhydride (189 mg, 0.96 mmol) and DIEA (2.53 mL, 12.8 mmol) were combined under N_2 in anhydrous THF (5 mL). The reaction mixture was stirred at room temperature for 6 h, before removal of the solvent under reduced pressure. The resultant residue was dissolved in DCM and washed with water (3 x 20 mL). The aqueous layers were extracted with DCM (1 x 20 mL) before the combined organic layers were

dried over Na_2SO_4 and the solvent was removed under reduced pressure to afford a dark yellow solid. **^{1}H NMR** ($CDCl_3$, 400 MHz, 298 K) 9.67 (1H, d, $^3J_{H-H}$ 8.5, H^4), 9.61 (1H,dd, $^3J_{H-H}$ 8.0, $^4J_{H-H}$ 2.0, H^9), 9.31 (1H, dd, $^3J_{H-H}$ 8.0, $^4J_{H-H}$ 2.0, H^7), 8.02 (1H, d, $^3J_{H-H}$ 8.5, H^3), 7.82 (1H,dd, $^3J_{H-H}$ 8.0, H^8), 7.71-7.68 (4H,m, Ar-H), 7.44-7.40 (6H, m, Ar-H), 5.81 (2H, s, CH_2), 3.22 (3H, s, CH_3). **ESI-HRMS** (+) calc. $[C_{28}H_{21}N_4O_3S]^+$ 493.1256, found 493.1234.

Tris(t-butyl) ester of L^2, 14

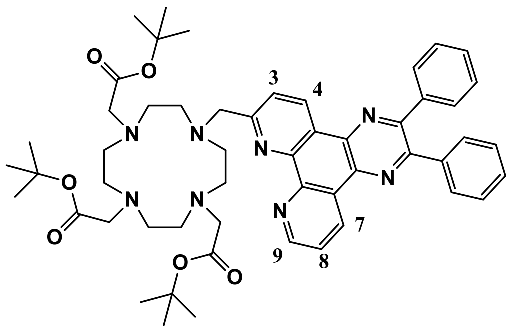

To a mixture of 1,4,7-tris(t-butoxycarbonylmethyl)-1,4,7,10-tetraazacyclododecane (35 mg, 0.07 mmol) and K_2CO_3 (18.8 mg, 0.14 mmol) in anhydrous acetonitrile (2 mL) under argon was added the mesylate **13** (33 mg, 0.07 mmol), and the mixture was heated at 60 ˚C for 18 h before being allowed to cool. The reaction solution was separated from the inorganic salts by filtration, solvent evaporated under reduced pressure and the residue was purified by column chromatography on silica gel (DCM-MeOH 0-5% gradient elution), to yield a pale yellow oil (37.6 mg, 59 %), **^{1}H NMR** ($CDCl_3$, 400 MHz, 298 K) 9.95 (1H, d, $^3J_{H-H}$ 8.0, H^9), 9.67 (1H, d, $^3J_{H-H}$ 7.0, H^4), 9.54 (1H, s, H^7), 8.28 (1H, s, H^3), 8.14 (1H, dd, H^8), 7.7-7.69 (4H, m, Ar-H), 7.47-7.40 (6H, m, Ar-H), 4.91 (2H, s, $C\underline{H}_2Ar$), 3.91-3.12 (22H, m, cyclen *NCH* and $C\underline{H}_2CO_2{}^tBu$), 1.50-1.25 (27H, m, $(CH_3)_3$); **^{13}C NMR** ($CDCl_3$, 101 MHz, 298 K,) δ 161.2 (3C, C=O), 154.7 (q Ar), 154.3 (q Ar), 154.8 (C^7), 138.8 (C^9), 138.2 (q Ar), 138.0 (q Ar), 137.5 (q Ar), 136.4 (q Ar), 135.7 (C^4), 130.3 (Ar), 130.2 (q Ar), 129.9 (Ar), 128.7 (Ar), 127.9 (C^3), 125.3 (C^8), 83.2 ($\underline{C}(CH_3)_3$), 58.1 ($\underline{C}H_2Ar$), 54.7 (12-N_4), 51.1 (12-N_4), 50.0 (12-N_4), 49.2 (12-N_4), 28.0 ($\underline{C}H_3$); **ESI-MS**: *m/z* 911.583 $[M+H]^+$. **ESI-HRMS** (+) calc. $[C_{53}H_{67}N_8O_6]^+$ 911.5105, found 911.5104.

Ligand $[H_6L^2]^{3+}$, 15 (as its triprotonated TFA salt)

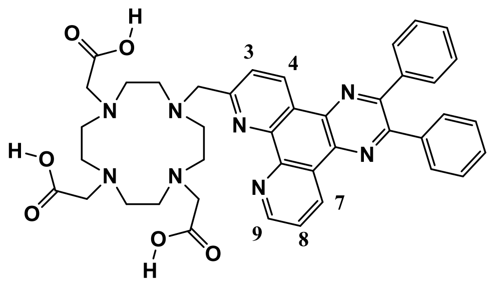

A solution of the *t*-butyl ester (62 mg, 0.068 mmol) in TFA/DCM (1:1) was prepared and stirred at room temperature for 2 h. After this time the solvent was removed under reduced pressure. The resulting residue was then re-dissolved in DCM (3 mL) before removal of this solvent under reduced pressure. This process was repeated 4 times. The residue was then dried under a high vacuum to afford the triprotonated ligand, as a pale orange residue. **ESI-HRMS** (+) calc. $[C_{41}H_{43}N_8O_6]^+$ 743.3227, found 743.3226.

[EuL²]

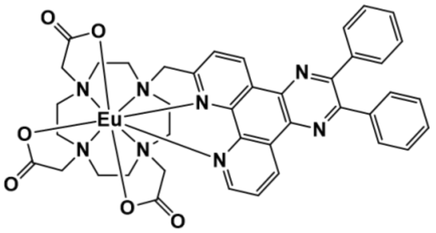

The protonated ligand L^2 (22 mg, 0.03 mmol,1 eq) was dissolved in an aqueous methanolic solution (3:1 methanol/water, 4 mL) and the pH was adjusted to 6.5. To this solution was added $EuCl_3.6H_2O$ (12mg, 0.033 mmol, 1.1 eq) followed by heating at 60 ˚C for 15 h. After this time the solution was separated from any inorganic salts by filtration and the solvent removed under reduced pressure. The residue was purified by preparative HPLC (*Method C*, t_R: 23.7 min) to afford the title complex; **ESI-HRMS** (+) calc. $[C_{41}H_{40}EuN_8O_6]^+$ 893.2278, found 893.2276. τ_{H_2O} (Eu) 1.05 ± 0.01 ms, λ_{max} 363 nm (13,000 M^{-1} cm^{-1}).

2.2 Ligands L^{3-4} (9-N-3-dpq) (Scheme 2)

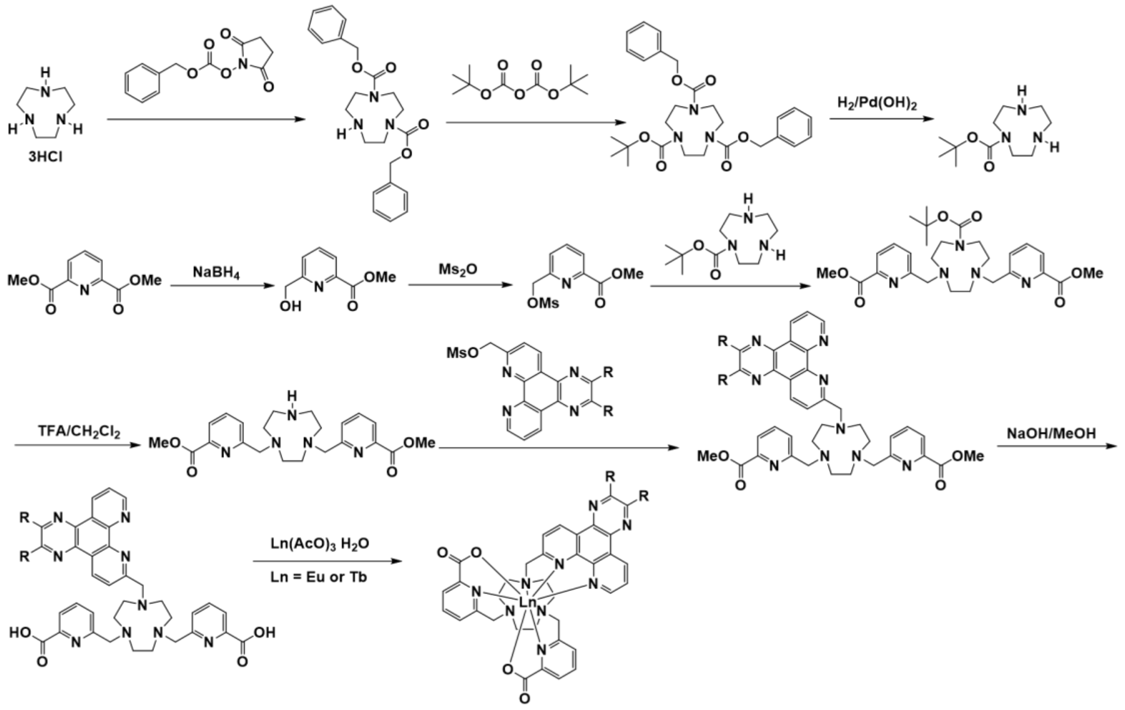

R = Me, L^3
R = Ph, L^4

Dibenzyl 1,4,7-triazacyclononane-1,4-dicarboxylate, 16 [5]

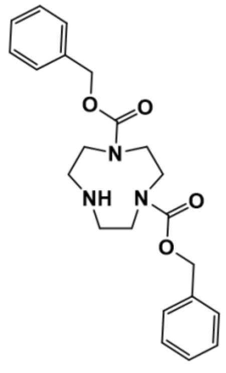

To a solution of the 1,4,7-triazacyclononane HCl salt (0.69 g, 2.89 mmol) in a dioxane/water (4:1) mixture was added N-(benzyloxycarbonyloxy) succinimide (1.44 g, 5.78 mmol) and triethylamine (0.8 ml, 5.78 mmol). The mixture was stirred at room temperature for 24 h before removing the solvent under reduced pressure. The resultant residue was dissolved in DCM and washed with brine (3 x 20 mL). The aqueous layers were re-extracted with DCM (3 x 20 mL) before the combined organic layers were dried over K_2CO_3 and the solvent was removed under reduced pressure. The resulting brown oil was purified by column chromatography (on silica gel, DCM-MeOH 0-5% gradient elution). The title product was obtained as a yellow viscous oil (0.4064 g, 35 %); R_f (silica, DCM-MeOH, 90:10): 0.47. **^{1}H NMR** ($CDCl_3$, 400 MHz, 298 K) 7.41-7.26 (10H,

m, CH), 5.18-5.08 (4H, m, CH_2), 3.57-3.53 (4H, m, ring CH_2), 3.34-3.21 (4H, m, ring CH_2), 2.94-2.78 (4H, m, ring CH_2); **^{13}C NMR** (298 K, 101 MHz, $CDCl_3$) δ 156.4 (1C,C=O), 156.3 (1C, C=O), 136.7 (2C, q Ar), 128.5-128.0 (10C, Ar), 67.2 (2C, CH_2), 52.8-47.6 (6C, cyclen *NCH_2*); **ESI-MS** (+) m/z: 398.792 $[M + H]^+$, 795.785 $[2M + H]^+$.

1,4-Dibenzyl 7-(tert-butyl) 1,4,7-triazacyclononane-1,4,7-tricarboxylate, 17

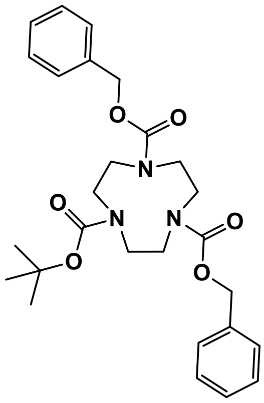

Dibenzyl 1,4,7-triazacyclononane-1,4-dicarboxylate, **16**, (0.2 g, 0.5 mmol) and di-tert-butyl dicarbonate (0.22 g, 1 mmol) were dissolved in chloroform (10 ml). The reaction mixture was stirred under an inert atmosphere, at room temperature, for 3 days. The reaction mixture was washed with brine (4 x 20 mL), and the aqueous layer was re-extracted with DCM (3 x 20 mL). The organic layers were combined, dried over $MgSO_4$ and the solvent removed under reduced pressure. The resulting yellow oil was purified by column chromatography (silica, DCM-MeOH 0-3% gradient elution) to yield the desired product as a colourless viscous oil (0.1864 g, 75 %). R_f (silica, DCM-MeOH, 90:10): 0.68. **^{1}H NMR** ($CDCl_3$, 400 MHz, 298 K) 7.36-7.28 (10H, m, Ar-H), 5.15-4.97 (4H, m, CH_2), 3.54-3.31 (12H, m, ring *NCH*), 1.46-1.40 (9H, m, ^{t}BOC CH_3); **^{13}C NMR** (298 K, 101 MHz, $CDCl_3$) δ 156.3 (2C, C=O), 155.6 (1C, C=O), 136.4 (2C, q Ar), 128.5-127.9 (10C, Ar), 80.0 (1C, $\underline{C}(CH_3)_3$), 67.2 (2C, CH_2), 50.6-48.6 (6C, cyclen *NCH_2*), 28.2 (3C, CH_3); **ESI-MS** (+) m/z: 498.904 $[M + H]^+$, 1017.220 $[2M + Na]^+$.

Tert-butyl 1,4,7-triazacyclononane-1-carboxylate, 18

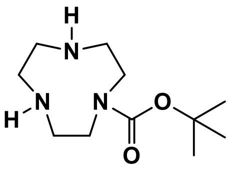

To a solution of 1,4-dibenzyl-7-(tert-butyl)-1,4,7-triazacyclononane-1,4,7-

tricarboxylate, **17**, (0.18 g, 0.36 mmol) in MeOH (15 ml) was added $Pd(OH)_2$ on carbon, (10 mg). The reaction mixture was placed under pressure (40 mbar, 295 K) on the compact vessel Parr hydrogenator for 12 h. The reaction mixture was filtered, and the solvent was removed under reduced pressure to give a pale-yellow oil [quantitative]. **^{1}H NMR** ($CDCl_3$, 400 MHz, 298 K) 3.43-3.32 (3H, m, ring CH), 3.27 (1H, s), 3.02-2.90 (4H, m, ring CH), 2.83 (3H, s), 1.33 (9H, s, CH_3); **^{13}C NMR** (298 K, 101 MHz, $CDCl_3$) δ 155.0 (1C, C=O), 80.8 (1C, $C(CH_3)_3$), 51.7, 51.6, 47.4, 46.7, 46.5, 46.2 (6C, ring CH), 28.4 (3C, CH_3); **ESI-MS** (+) m/z: 230.636 $[M + H]^+$, 459.896 $[2M + H]^+$.

Compounds 19 and 20 were prepared according to established literature procedures. [6]

Methyl 6-(hydroxymethyl)picolinate, 19 [6]

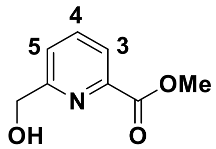

Dimethyl pyridine-2,6-dicarboxylate (0.3 g, 1.54 mmol) was dissolved in DCM/EtOH (1:1, 20 ml) and cooled in an ice bath. $NaBH_4$ (0.064 g, 1.69 mmol) was added, and the reaction was stirred at 0 ˚C and progress was monitored by TLC. After 30 min, 1 M HCl (1 mL) was added. The volatile solvents were removed under reduced pressure and the aqueous solution was extracted three times with ethyl acetate (3 x 20 mL). The organic layers were combined, dried over $MgSO_4$, filtered and concentrated. The crude product was purified by silica gel column chromatography (DCM-MeOH, 0-5% gradient elution) to yield the title product as a white solid [yield 84%, m.p. 88.7-90.8 ˚C /Lit. [7] 89.5-91.5 ˚C]. R_f (silica, DCM-MeOH, 90:10): 0.49. **^{1}H NMR** ($CDCl_3$, 400 MHz, 298 K) 8.03 (1H, d, $^3J_{H-H}$ 8.0 Hz, H^3), 7.85 (1H, dd, $^3J_{H-H}$ 8.0 Hz, H^4), 7.54 (1H, d, $^3J_{H-H}$ 8.0 Hz, H^5), 4.86 (2H, s, CH_2), 3.99 (3H, s, CH_3). **^{13}C NMR** (298 K, 101 MHz, $CDCl_3$) δ 165.7 (1C, C=O), 160.3 (1C, py-C^6), 147.2 (1C, py-C^2), 137.8 (1C, C^4), 124.2 (1C, C^5), 124.0 (1C, C^3), 64.7 (1C, CH_2), 53.0 (1C, CH_3).

Methyl-6-[(methylsulfonato) methyl]pyridine-2-carboxylate, 20 [6]

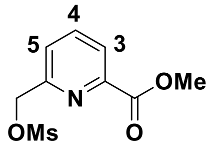

Methyl 6-(hydroxymethyl)picolinate, **19**, (0.166 g, 1 mmol) was dissolved in anhydrous THF (5 ml) and cooled to 0 ˚C under argon. DIPEA (1.4 ml, 8 mmol)

was added, followed by the dropwise addition of methanesulfonic anhydride (0.696 g, 4 mmol). The mixture was stirred under argon for 6 h. The solvent was removed under reduced pressure and residue redissolved in DCM (15 mL), washed with water (2 x 10 mL) and the aqueous layer was extracted with DCM (2 x 15 mL). The organic layers were combined, dried over $MgSO_4$, filtered, and concentrated to give a crude product which was used immediately without further purification, R_f (silica, DCM-MeOH, 90:10): 0.61. **^{1}H NMR** ($CDCl_3$, 400 MHz, 298 K) 7.87 (1H, d, $^3J_{H-H}$ 8.0, H^3), 7.74 (1H, t, $^3J_{H-H}$ 8.0, H^4), 7.55 (1H, d, $^3J_{H-H}$ 8 .0, H^5), 4.57 (2H, s, CH_2), 3.79 (3H, s, OCH_3), 2.54 (3H, s, CH_3).

Dimethyl 6,6'-[(7-(tert-butoxycarbonyl)-1,4,7-triazacyclononane-1,4-diyl) bis(methylene)]dipicolinate, 21

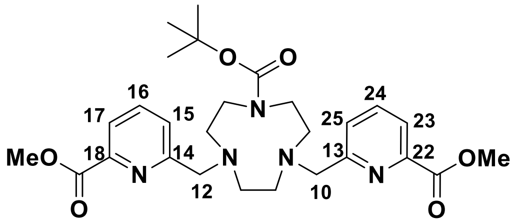

To a mixture of *tert*-butyl 1,4,7-triazacyclononane-1-carboxylate, **18**, (0.12 g, 0.53 mmol) and K_2CO_3 (0.4 g, 2.88 mmol) in anhydrous acetonitrile (10 ml) under argon was added the methyl-6-[(methylsulfonato)methyl]pyridine-2-carboxylate, **20**, (0.35 g, 1.44 mmol). The mixture was heated at 60 ˚C for 18 h before being allowed to cool. The reaction solution was filtered and purified by column chromatography (silica, DCM-MeOH 5-8% gradient elution) to yield the desired product as a viscous oil (0.1899 g, 68 %). TLC analysis R_f = 0.35 (silica, 10% CH_3OH in CH_2Cl_2). **^{1}H NMR** (298 K, 400 MHz, $CDCl_3$) 7.96 (2H, d, $^3J_{H-H}$ 7.0 Hz, H^3), 7.82-7.72 (4H, m, $H^{4,5}$), 4.04 (4H, s, NCH_2), 3.94 (6H, s, OCH_3), 3.43-2.66 (12H, m, ring *CHN*), 1.40 (9H, s, $(C\underline{H}_3)_3$). **^{13}C NMR** (298 K, 101 MHz, $CDCl_3$) δ 165.8 (2C, $\underline{C}OOCH_3$), 160.1 (2C, py-^{13,14}C), 155.6 (1C, $\underline{C}OO(CH_3)_3$), 147.3 (2C, py-^{16,23}C), 137.6 (2C, py-^{18,21}C), 126.8 (2C, py-^{17,22}C), 123.8 (2C, py-^{19,20}C), 79.7 (1C, $\underline{C}(CH_3)_3$), 63.2 (2C, py-$\underline{C}H_2$), 56.5-49.7 (6C, ring *CHN*), 52.9 (2C, $O\underline{C}H_3$), 28.6 (3C, $(\underline{C}H_3)_3$); **ESI-MS** (+) *m/z* 528.737 $[M+H]^+$; **ESI-HRMS (+) calc.** $[C_{27}H_{38}N_5O_6]^+$ 528.2816, found 528.2794.

Dimethyl-6,6'-((1,4,7-triazacyclononane-1,4-diyl) bis(methylene))dipicolinate, 22

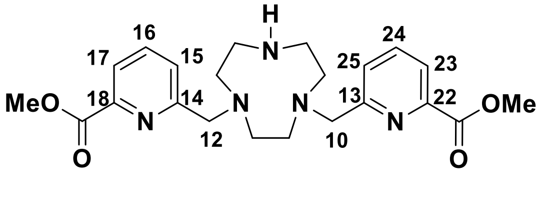

A solution of **compound 21** (30 mg, 0.057 mmol) in TFA/DCM (1:1) was prepared and stirred at room temperature for 6 h. After this time the solvent was removed under reduced pressure. The resulting residue was then re-dissolved in DCM (20 mL) before the removal of this solvent under reduced pressure. This process was repeated 4 times. The residue was then dried under a high vacuum to afford the desired product as its di-protonated TFA salt. (quantitative) **^{1}H NMR** (298 K, 400 MHz, $CDCl_3$) 7.91 (2H, d, $^3J_{H-H}$ 8.0 Hz, $H^{17,23}$), 7.83 (2H, dd, $^3J_{H-H}$ 8.0 Hz $H^{16,24}$), 7.48 (2H, d, $^3J_{H-H}$ 8.0 Hz, $H^{15,25}$), 4.5 (4H, s, NCH_2), 3.77 (10H, s, ring *CH* and CH_3), 3.68(4H, br, ring *CH*), 3.49 (4H, br, ring *CH*). **^{13}C NMR** (298 K, 101 MHz, $CDCl_3$) δ 165.0 (2C, <u>C</u>OOCH$_3$), 155.8 (2C, py-^{13,14}C), 146.8 (2C, py-^{18,22}C), 138.6 (2C, py-^{16,24}C), 126.5 (2C, py-^{15,25}C), 124.6 (2C, py-^{17,23}C), 60.6 (2C, py-<u>C</u>H$_2$), 53.9-47.7 (6C, ring *CHN)*, 53.0 (2C, O<u>C</u>H$_3$); **ESI-MS** (+) *m/z* 428.696 [M+H]$^+$; **ESI-HRMS (+) calc.** $[C_{22}H_{30}N_5O_4]^+$ 428.2292, found 428.2278.

L^3 and L^4 (dimethyl esters)

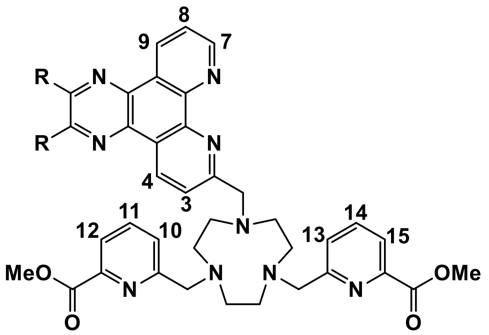

To a mixture of **compound 22** (18 mg, 0.043 mmol) and K_2CO_3 (12 mg, 0.086 mmol) in anhydrous acetonitrile (2 ml) under argon was added the mesylate chromophore (dpqPh$_2$-OMs or dpqMe$_2$-OMs) (21 mg or 17 mg). The mixture was heated at 60 °C for 18 h before being allowed to cool. The reaction solution was filtered and removed under the reduced pressure. The crude residue was purified by RP-HPLC (*Method J*, ***t***$_R$: 14.7 min for L^3; *Method K*, ***t***$_R$: 16.4 min for L^4) to afford the title complex as a dark yellow viscous oil. (**L^3**: 5 mg, 16 %; **L^4**: 7 mg, 20 %)

L^3: ^{1}H NMR (298 K, 400 MHz, $CDCl_3$) 10.02 (1H, d, $^3J_{H-H}$ 8.0 Hz, H^7), 9.58 (1H, d, $^3J_{H-H}$ 8.5 Hz, H^4), 9.48 (1H, d, $^3J_{H-H}$ 4.5 Hz, H^9), 8.23 (1H, m, H^8), 8.13 (1H, d, $^3J_{H-H}$ 8.5 Hz, H^3), 7.87 – 7.78 (4H, m, $H^{11,12,14,15}$), 7.52 (2H, d, $^3J_{H-H}$ 8.0 Hz, $H^{10,13}$), 4.85 (2H, s, CH_2Ar), 4.54 (4H, s, CH_2-py), 3.96 – 3.73 (8H, m, ring CH_2), 3.66 (6H, s, OCH_3), 3.58 (4H, s, ring CH_2), 2.89 (6H, s, CH_3); **^{13}C NMR** (298 K, 101 MHz, $CDCl_3$) δ 165.0 (2C, C=O), 156.4 (2C, q Ar), 155.5 (1C, q Ar), 155.5 (2C, q Ar), 146.8 (2C, q Ar), 146.1 (1C, C^9), 140.3 (1C, C^7), 138.3 (2C, $C^{11,14}$), 137.3 (1C, q Ar), 135.5 (1C, q Ar), 135.3 (1C, C^4), 129.3 (1C, q Ar), 128.4 (1C, q Ar), 127.3 (1C, C^3), 126.6 (2C, $C^{10,13}$), 125.3 (1C, C^8), 124.4 (2C, $C^{12,15}$), 61.7 (1C, CH_2Ar), 61.3 (1C, CH_2-py), 54.7 (2C, ring NCH), 54.2 (2C, ring NCH), 53.0

(2C, ring NCH), 52.8 (2C, OCH_3), 23.2 (2C, CH_3); **ESI-MS** (+) *m/z* 699.989 $[M+H]^+$; **ESI-HRMS (+)** calc. $[C_{39}H_{42}N_9O_4]^+$ 700.3354, found 700.3375.

L^4: ^{1}H NMR (298 K, 400 MHz, $CDCl_3$) 10.26 (1H, d, $^3J_{H\text{-}H}$ = 8.0 Hz, H^7), 9.78-9.69 (1H, m, H^4), 9.51 (1H, br, H^9), 8.42 (1H, d, $^3J_{H\text{-}H}$ = 8.5 Hz, H^8), 8.21 (1H, br, H^3), 7.81 (4H, m, H^{11,12,14,15}), 7.72 (4H, m, H^{10,13} and Ar-H), 7.51-7.41 (8H, m, Ar-H), 5.08 (2H, s, CH_2Ar), 4.63 (4H, s, CH_2-py), 4.11-3.72 (12H, m, ring CH_2), 3.55 (6H, s, OCH_3); **^{13}C NMR** (298 K, 101 MHz, $CDCl_3$) δ 164.9 (2C, C=O), 155.8 (2C, q Ar), 155.0 (1C, q Ar), 153.5 (2C, q Ar), 146.3 (2C, q Ar), 145.3 (1C, C^9), 142.9 (1C, C^7), 139.8 (1C, q Ar), 138.8 (1C, q Ar), 138.5 (2C, C^{11,14}), 137.8 (1C, q Ar), 137.6 (1C, q Ar), 136.5 (1C, C^4), 135.2 (1C, q Ar), 130.3 (2C, Ar C), 130.2 (2C, Ar C), 130.1 (2C, Ar C), 129.8 (1C, q Ar), 129.1 (1C, q Ar), 128.8 (2C, Ar C), 128.7 (2C, Ar C), 128.2 (1C, C^3), 126.1 (2C, C^{10,13}), 126.1 (1C, C^8), 124.4 (2C, C^{12,15}), 61.6 (3C, $\underline{C}H_2$), 55.1 (2C, ring NCH), 53.6 (2C, ring NCH), 53.5 (2C, ring NCH), 52.8 (2C, OCH_3); **ESI-MS** (+) *m/z* 824.496 $[M+H]^+$; **ESI-HRMS (+) calc.** $[C_{49}H_{46}N_9O_4]^+$ 824.3667, found 824.3617.

[LnL$^{3-4}$], (R = Me, L^3; R = Ph, L^4) (Ln = Eu or Tb), 23-25

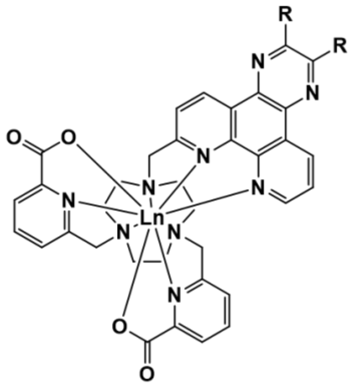

R = Ph or Me,
Ln = Eu or Tb

The dimethyl ester of L^3 or L^4 (0.012 mmol) was suspended in aqueous sodium hydroxide solution (5 mL, 0.1 M) and acetonitrile (ca. 2 mL) was added dropwise until the solid dissolved. The solution was stirred at room temperature for 16 h. The solution was neutralised using dilute hydrochloric acid and the solvent was removed under reduced pressure. The resulting solid was re-dissolved in acetonitrile (3 ml) and $Eu(OAc)_3$ or $Tb(OAc)_3$ (0.0144 mmol) was added. The reaction solution was stirred at 65 °C for 18 h. After this time, the solvent was removed under reduced pressure. The residue was purified by preparative HPLC (**LnL^3**: *Method F*, t_R: 15.4 min; **LnL^4**: *Method G*, t_R: 29.4 min) **[EuL^3]: ESI-HRMS** (+) calc. $[C_{37}H_{35}EuN_9O_4]^+$ 822.2024, found 822.2025. τ_{H_2O} (Eu) 0.96 ± 0.01 ms, λ_{max} 345 nm (3300 M^{-1} cm^{-1}). **[TbL^3]: ESI-HRMS** (+) calc. $[C_{37}H_{35}TbN_9O_4]^+$ 828.2065, found 828.2062. τ_{H_2O} (Tb) 1.60 ± 0.01 ms, λ_{max} 345 nm (6000 M^{-1} cm^{-1}). **[EuL^4]: ESI-HRMS** (+) calc. $[C_{47}H_{39}EuN_9O_4]^+$ 946.2337, found 946.2323. τ_{H_2O} (Eu) 0.95 ± 0.01 ms, λ_{max} 363 nm (30,000 M^{-1} cm^{-1}).

2.3 Synthesis of L^{5-6} (Scheme 3)

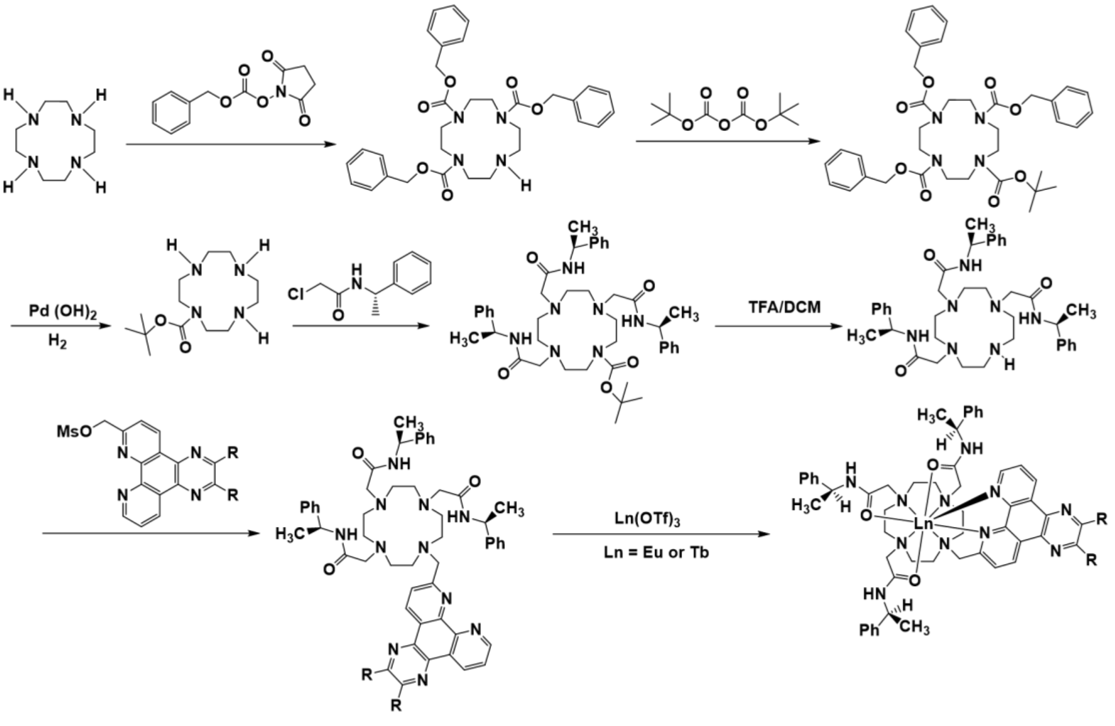

R = Me, L^5
R = Ph, L^6

Compounds 26 to 31 were prepared according to established literature procedures" [7,9-12].

2-Chloro-N-[(*S*)-1-phenylethyl]ethanamide, 26

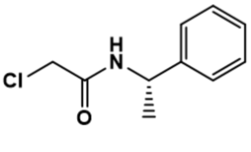

Chloroacetyl chloride (1.24 ml, 15.66 mmol) was added dropwise to a vigorously stirring solution of (*S*)-(-)-α-methylbenzylamine (1.06 ml, 8.25 mmol) and NEt_3 (1.25 ml, 9.05 mmol) in dry Et_2O (20 ml) at -20 ˚C. Following complete addition, the solution was allowed to warm to room temperature over the course of 2 h yielding a yellow tinged solution containing a white precipitate. The suspension was washed with HCl (aq) (1 M, 2 x 4 ml) followed by H_2O (2 x 10 ml) to yield a clear yellow tinged organic phase that was cooled at 4 ˚C to precipitate the crude product as off-white needle-like crystals. The desired product was purified by recrystallisation from Et_2O and was isolated as white needle-like crystals, m.p. 101.0-101.5 ˚C (Lit. 101-102 ˚C [8]). **^{1}H NMR** ($CDCl_3$, 400 MHz, 298 K) 7.40-7.24 (5H, m, Ar), 6.83 (1H, s, NH), 5.14 (1H, m, CH), 4.11-3.98 (2H, m, CH_2), 1.54 (3H, d, $^3J_{H-H}$ 7.0 Hz, CH_3).

Tribenzyl 1,4,7,10-tetraazacyclododecane-1,4,7-tricarboxylate, 27

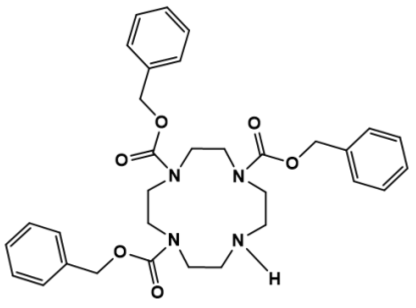

N-(Benzyloxycarbonyloxy) succinimide (9.07 g, 36.4 mmol) and NEt_3 (5.4 ml, 39 mmol) were added to a solution of cyclen (2.24 g, 13 mmol) in water/dioxane (4:1). The mixture was stirred at room temperature for 8 h before removing the solvent under reduced pressure. The resultant residue was dissolved in DCM and washed with brine (3 x 20 mL). The aqueous layers were re-extracted with DCM (3 x 20 mL) before the combined organic layers were dried over K_2CO_3 and the solvent was removed under reduced pressure. The resulting brown oil was purified by column chromatography (on silica gel, DCM-MeOH 0-5% gradient elution). The title product was obtained as a transparent oil. R_f =0.59 (silica, 10% CH_3OH in CH_2Cl_2); **^{1}H NMR** ($CDCl_3$, 400 MHz, 298 K) 7.34-7.27 (15H, m, Ar), 5.13 (1H, s, C<u>H</u>H'), 5.04 (4H, s, CH_2), 4.86 (1H, s, CH<u>H'</u>), 3.73 - 3.61 (4H, m, cyclen *NCH_2*), 3.47-3.30 (8H, m, cyclen *NCH_2*), 2.87-2.79 (4H, m, cyclen *NCH_2*). **^{13}C NMR** (298 K, 101 MHz, $CDCl_3$) δ 156.5 (C=O), 156.1 (C'=O), 137.1 (1C, q Ar), 136.8 (2C, q Ar), 128.5 (Ar), 128.5 (Ar), 128.2 (Ar), 128.0 (Ar), 127.9 (Ar), 127.8 (Ar), 67.2 (CH_2), 66.9 (CH_2), 51.2-49.1 (br s, cyclen C), 45.7 (cyclen C); **ESI-MS** (+) m/z: 575.893 $[M + H]^+$, 1151.011 $[2M + H]^+$.

1,4,7-Tribenzyl 10-(tert-butyl) 1,4,7,10-tetraazacyclododecane-1,4,7,10-tetracarboxylate, 28

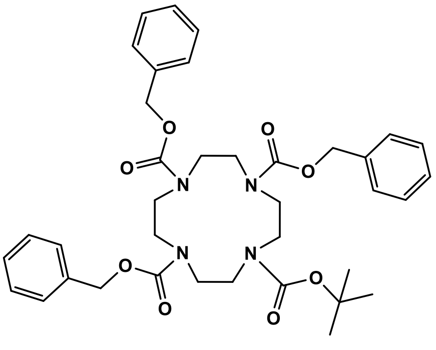

Compound **27** (2.1g, 3.6 mmol), NEt_3 (1 ml, 7.2 mmol), and di-tert-butyl dicarbonate (1.7 ml, 7.2 mmol) were dissolved in chloroform (10 ml). The reaction mixture was stirred under an inert atmosphere, at room temperature,

for 16 h. The reaction was stopped and washed with sat. NaCl solution (4 x 10 mL). The aqueous layer was re-extracted with DCM (3 x 10 mL). The organic layer was combined, dried over $MgSO_4$ and the solvent was removed under reduced pressure. The resulting oil was purified by column chromatography (silica, DCM-MeOH 0-4% gradient elution) to yield the desired product as a colourless thick oil. R_f = 0.71 (silica, 10% CH_3OH in CH_2Cl_2). **^{1}H NMR** ($CDCl_3$, 400 MHz, 298 K) 7.31-7.25 (15H, m, Ar), 5.07 (4H, s, CH_2), 5.04 (2H, s, CH_2), 3.43-3.30 (16H, m, cyclen *NCH_2*), 1.36 (9H, s, $(CH_3)_3$); **^{13}C NMR** ($CDCl_3$, 101 MHz, 298 K) δ 156.6 (4C, C=O), 136.4 (3C, q Ar), 128.5 (Ar), 128.4 (Ar), 128.2 (Ar), 128.2 (Ar), 128.2 (Ar), 80.1 (1C, <u>C</u>$(CH_3)_3$), 67.2 (3C, CH_2), 50.2-50.1 (8C, br, cyclen C), 28.4 (3C, CH_3); **ESI-MS** (+) m/z: 675.717 $[M + H]^+$.

Tert-butyl 1,4,7,10-tetraazacyclododecane-1-carboxylate, 29

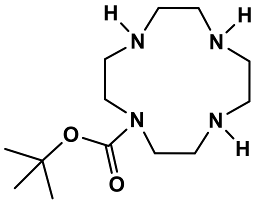

1,4,7-Tribenzyl-10-(tert-butoxycarbonyl)-1,4,7,10-tetraazacyclododecane- (1.44 g, 2.13 mmol) in MeOH (15 ml) was stirred in a Parr hydrogenation flask at 40 mbar H_2 over Pd $(OH)_2$ on carbon (0.13 g) for 8 h. The resulting mixture was filtered to leave a clear solution that was dried under reduced pressure to yield the desired product as a glassy white oil [quantitative]. **^{1}H NMR** ($CDCl_3$, 400 MHz, 298 K) 3.47 (1H, br, NH), 3.42-3.34 (5H, m, cyclen CH_2), 3.28 (2H, s), 2.85 (2H, br, NH), 2.82-2.75 (6H, m, cyclen CH_2), 2.64-2.56 (3H, m, cyclen CH_2), 1.35 (9H, s, CH_3); **^{13}C NMR** ($CDCl_3$, 101 MHz, 298 K) δ 156.0 (C=O), 155.4 (C=O), 80.4 (<u>C</u>$(CH_3)_3$), 80.1 (<u>C</u>$(CH_3)_3$), 50.7- 45.8 (8C, cyclen C), 28.4 (CH_3); **ESI-MS** (+) m/z: 273.209 $[M + H]^+$, 545.459 $[2M + H]^+$.

Tert-butyl-4,7,10-tris(2-oxo-2-(((S)-1-phenylethyl)amino)ethyl)-1,4,7,10-tetraazacyclododecane-1-carboxylate, 30

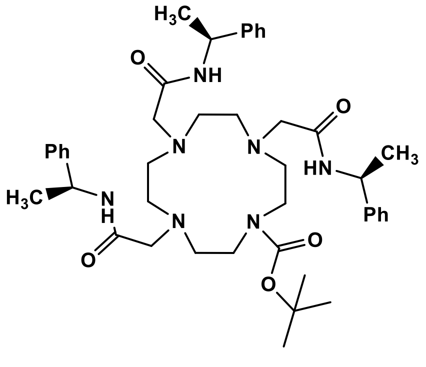

A mixture of compound **29** (270 mg, 1 mmol), 2-chloro-N-[(*S*)-1-phenylethyl]ethanamide (630 mg, 3.2 mmol), Cs_2CO_3 (0.98 g, 3 mmol) and KI (5 mg) was stirred and boiled under reflux in dry MeCN (15 mL) for 24 h. The resultant solution was allowed to cool to room temperature and then dried under reduced pressure. The residue was taken up in DCM (15 ml), washed with sodium thiosulfate solution (0.01 M, 10 ml) then H_2O (2 x 15 ml). The organic layers were combined and dried over $MgSO_4$, and the solvent was removed under reduced pressure. The resulting oil was purified by column chromatography (silica, DCM-MeOH 0-4% gradient elution) to yield the desired product [yield 65 %, R_f = 0.40 (silica, 10% CH_3OH in CH_2Cl_2)]. **^{1}H NMR** ($CDCl_3$, 400 MHz, 298 K) 7.30-7.20 (16H, m, Ar and amide NH), 7.05-6.92 (2H, m, amide NH), 5.15-5.08 (3H, m, CH), 3.25-2.46 (22H, m, cyclen *NCH_2* and CH_2), 1.48-1.40 (18H, m, $(CH_3)_3$ and arm CH_3). **^{13}C NMR** (298 K, 101 MHz, $CDCl_3$) δ 170.2 (1C, amide C=O), 169.7 (1C, amide C=O), 155.5 (1C, ^{t}BOC C=O), 143.7 (1C, q Ar), 143.1 (2C, q Ar), 129.1-126.3 (15C, Ar), 80.5 (1C, <u>C</u>$(CH_3)_3$), 53.7 (3C, CH_2CO), 59.5-47.4 (11C, cyclen CH_2 and arm CH_2), 48.4 (3C, CH), 28.6 (3C, ^{t}BOC CH_3), 21.9 (1C, CH_3), 21.3 (2C, CH_3). **ESI-MS** (+) m/z: 756.951 [M + H]$^+$.

2,2',2''-(1,4,7,10-Tetraazacyclododecane-1,4,7-triyl)tris(N-((*S*)-1-phenylethyl)acetamide), 31

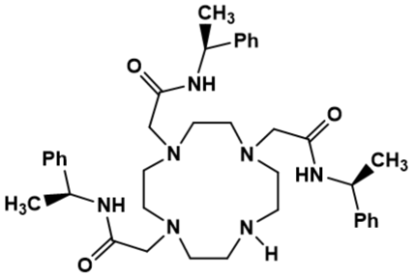

Compound **30** (72 mg, 0.095 mmol) in TFA: DCM (1:1, 4 ml) was stirred at room temperature for 14 h to yield a yellow solution. Solvent was then removed under reduced pressure to yield the desired product as its deprotonated TFA salt in quantitative yield. **^{1}H NMR** ($CDCl_3$, 400 MHz, 298 K) 8.76 (1H, s, amide NH), 8.13 (1H, s, amide NH), 7.62 (1H, s, amide NH), 7.31-7.19 (15H, m, Ar H), 4.97-4.79 (3H, m, CH), 3.56-2.87 (22H, m, cyclen *NCH_2* and CH_2), 1.50-1.33 (9H, m, CH_3); **^{13}C NMR** (298 K, 101 MHz, $CDCl_3$) δ 170.7 (3C, C=O), 143.4 (2C, q Ar), 143.3 (1C, q Ar), 128.8-126.1 (15C, Ar), 55.5-43.2 (11C, cyclen CH_2 and arm CH_2), 50.1 (3C, CH), 22.0 (3C, CH_3); **ESI-MS** (+) m/z: 656.026 [M + H]$^+$.

Ligands $L^{5\text{-}6}$, (R = Me, L^5; R = Ph, L^6), 32-33

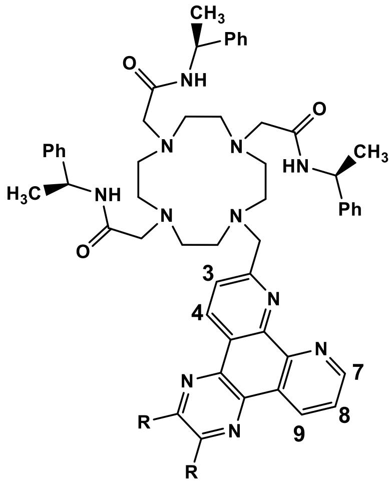

To a mixture of compound **31** (46 mg, 0.07 mmol) and K_2CO_3 (199 mg, 0.14 mmol) in anhydrous acetonitrile (2 ml) under argon was added the appropriate mesylate ($dpqPh_2$-OMs or $dpqMe_2$-OMs) (34 mg or 26 mg, 0.07 mmol). The mixture was heated at 60 ˚C for 18 h before being allowed to cool. The reaction solution was filtered, and the solvent was removed under reduced pressure. The residue was purified by preparative HPLC to afford the title complex as viscous oil. (**LnL^5**: *Method L*, t_R: 21.3 min, 9 mg, 14 %; **LnL^6**: *Method M*, t_R: 22.6 min, 4 mg, 5 %). **L^5: ^{1}H NMR** (298 K, 400 MHz, $CDCl_3$) 10.10 (1H, d, $^3J_{H\text{-}H}$ 8.0 Hz, H^7), 9.48 (1H, d, $^3J_{H\text{-}H}$ 8.0 Hz, H^4), 8.27-8.20 (2H, m, $H^{8,9}$), 8.01 (1H, br, H^3), 7.35-7.07 (15H, m, Ar-H), 5.01-4.72 (3H, m, CH), 3.76-2.98 (24H, m, cyclen NCH_2 and CH_2), 2.97-2.08 (9H, m, amide CH_3), 1.46-1.37 (6H, m, py-CH_3); **^{13}C NMR** (298 K, 151 MHz, $CDCl_3$) δ 157.2 (2C, C=O), 156.1 (1C, C=O), 144.2 (q Ar), 143.3 (q Ar), 142.4 (C^7), 138.7 (q Ar), 138.0 (q Ar), 137.4 (q Ar), 135.4 (C^4), 134.7(q Ar), 129.5 (Ar), 128.8 (Ar), 128.8 (C^3), 128.7 (Ar), 128.7 (Ar), 128.5 (Ar), 127.6 (Ar), 127.6 (Ar), 127.5 (Ar), 126.4 (Ar), 126.2 (Ar), 126.1 (Ar), 125.9 ($C^{8,9}$), 54.5-51.1 (cyclen NCH_2 and arm CH_2), 50.3 (1C, CH), 49.5 (2C, CH), 23.3 (1C, amide CH_3), 23.2 (2C, amide CH_3), 21.8 (2C, CH_3); **ESI-MS** (+) *m/z* 928.495 $[M+H]^+$; **ESI-HRMS (+) calc.** $[C_{55}H_{66}N_{11}O_3Na]^+$ 950.5164, found 950.5201.

L^6: **^{1}H NMR** (298 K, 400 MHz, $CDCl_3$) 9.97 (1H, d, $^3J_{H\text{-}H}$ 8.0 Hz, H^7), 9.50 (1H, d, $^3J_{H\text{-}H}$ 8.0 Hz, H^4), 9.37-9.27 (1H, m, H^7), 8.73 (2H, m, $H^{8,9}$), 8.20-8.10 (2H, br, NH), 7.73-7.65 (6H, m, Ar-H), 7.50-7.39 (9H, m, Ar-H), 7.23-6.96 (11H, m, Ar-H and NH), 5.12-4.74 (4H, m, CH), 4.55-4.32 (2H, m, arm CH_2), 3.59-2.82 (22H, m, cyclen NCH_2 and CH_2), 1.47-1.19 (9H, CH_3); **^{13}C NMR** (298 K, 151 MHz, $CDCl_3$) δ 168.2 (2C, C=O), 166.3 (1C, C=O), 164.5 (1C, q Ar), 143.7 (q Ar), 143.0 (C^7), 138.0 (q Ar), 136.7 (q Ar), 135.6 (q Ar), 135.4 (q Ar), 134.0 (C^4), 132.0 (Ar), 131.3 (Ar), 130.2 (Ar), 130.1 (Ar), 129.0 (Ar), 128.7 (Ar), 128.6 (Ar), 127.2 (C^3), 126.4 (Ar), 126.0 ($C^{8,9}$), 52.8 (3C, CH), 50.2-48.6 (cyclen NCH_2 and arm CH_2), 22.0 (CH_3), 21.6 (CH_3); **ESI-MS** (+) *m/z* 1052.150 $[M+H]^+$; **ESI-HRMS (+) calc.** $[C_{65}H_{70}N_{11}O_3]$+ 1052.5657, found 1052.5696.

[LnL$^{5-6}$], (R = Me, L^5; R = Ph, L^6) (Ln = Eu or Tb), 34-36

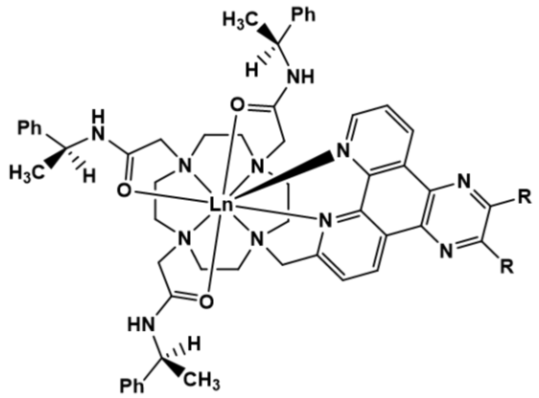

A solution of ligand L^5 or L^6 as their TFA salts (10 mg) and Eu $(OTf)_3$ or Tb $(OTf)_3$ in dry MeCN (1 ml) was heated at reflux under argon for 24 h. Solvent was removed under reduced pressure to leave an orange solid. DCM (2 ml) was added to the solid, which was sonicated for 2 min, solvent was then decanted to leave an orange residue. The sonication process was repeated followed by drying of the remaining residue, under reduced pressure, to yield the triflate salt of the desired product. The crude product was purified by preparative HPLC (LnL^5: *Method D*, t_R= 31.0 min; LnL^6: *Method H*, t_R= 31.0). The desired products were made water soluble by exchanging the triflate anion for a chloride anion using methanol-washed anion exchange resin. ('DOWEX 1x4 50-100 mesh Cl') **[EuL^5]: ESI-HRMS** (+) calc. $[C_{55}H_{65}Eu_{11}O_3]^+$ 1080.4484, found 1078.4316. τ_{H_2O} (Eu) 1.04 ± 0.01 ms, λ_{max} 345 nm (4900 M^{-1} cm^{-1}). **[TbL^5]: ESI-HRMS** (+) calc. $[C_{55}H_{65}Tb_{11}O_3]^+$ 1086.4525, found 1084.4361. τ_{H_2O} (Tb) 2.2 ± 0.01 ms, λ_{max} 345 nm (4200 M^{-1} cm^{-1}). **[EuL^6]: ESI-HRMS** (+) calc. $[C_{65}H_{69}EuN_{11}O_3]^+$ 1204.4797, found 1200.4618. τ_{H_2O} (Eu) 1.0 ± 0.01 ms, λ_{max} 364 nm (17,000 M^{-1} cm^{-1}).

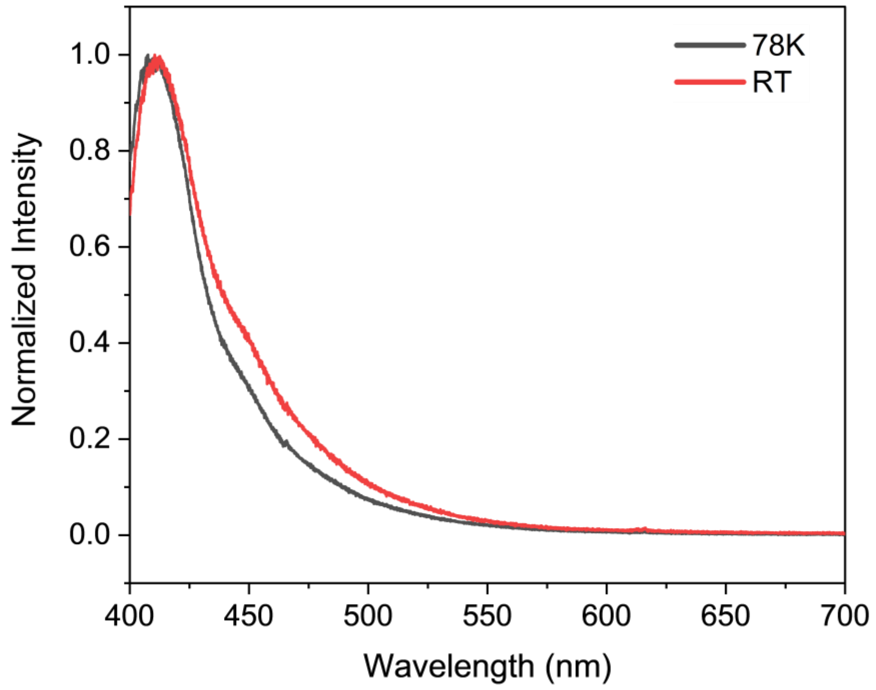

Fig. S1 Photoluminescence spectra of L^2 excited at 375 nm. The spectrum is nearly the same at room temperature and 78K. No phosphorescence signal is detected.

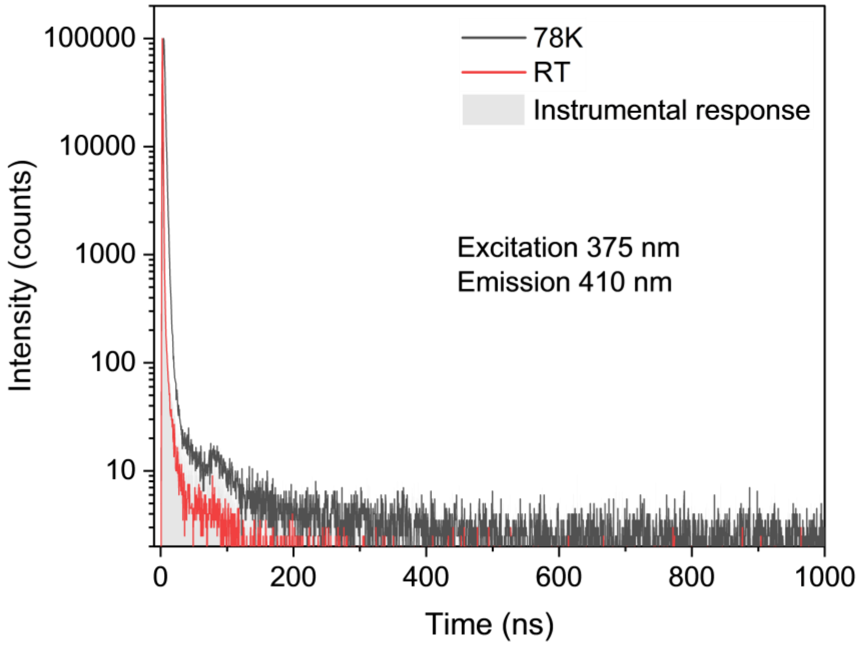

Fig. S2 The PL decay curves of L^2 excited at 375 nm at room temperature and 78K. The PL decay is beyond the time resolution of our equipment.

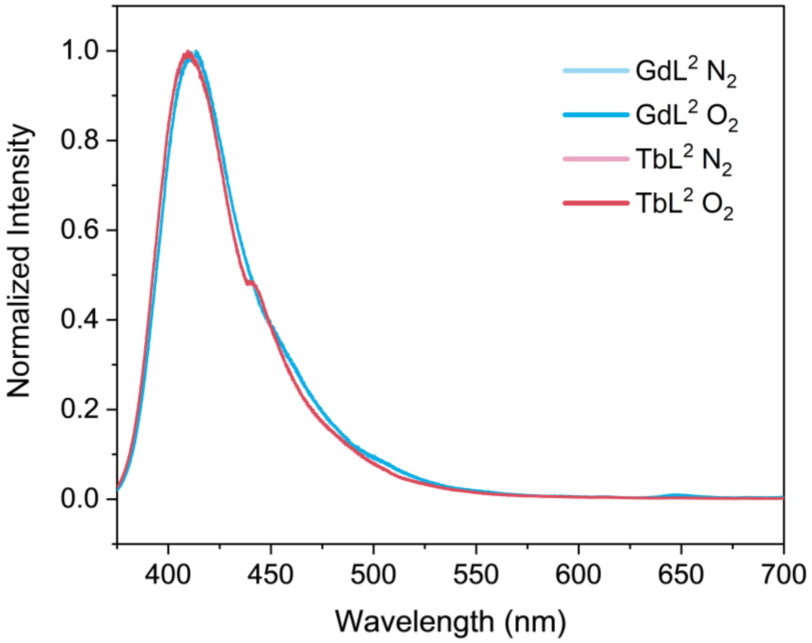

Fig. S3 The photoluminescence spectra of [GdL^2] and [TbL^2] excited at 360 nm at room temperature under different atmospheres. The spectra are nearly identical.

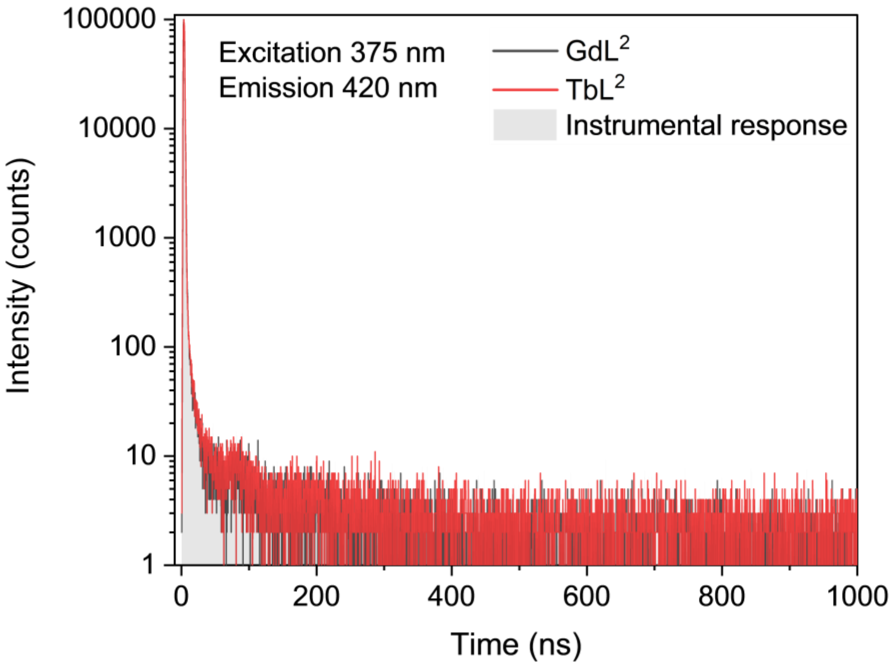

Fig. S4 The PL decay curves of [GdL^2] and [TbL^2] excited at 375 nm at room temperature. The PL decay is beyond the time resolution of our equipment.

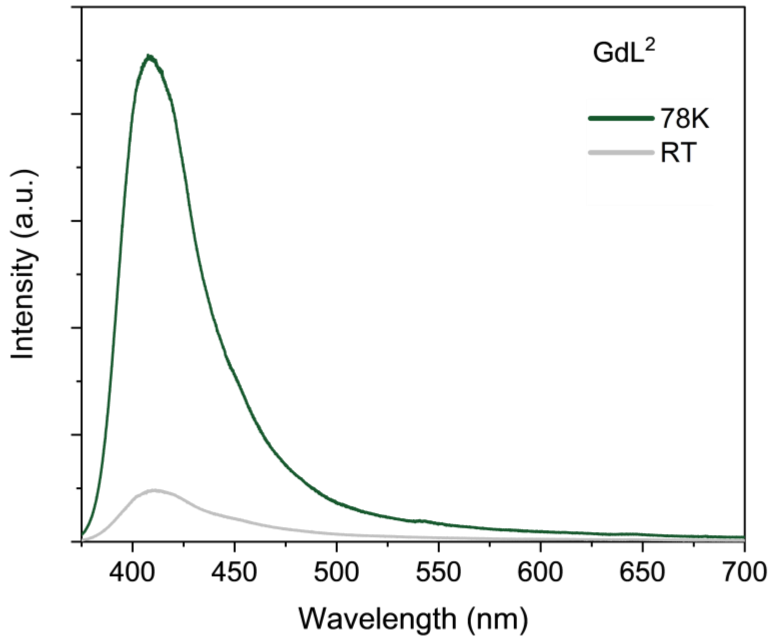

Fig. S5 The photoluminescence spectra of [GdL^2] excited at 360 nm at room temperature and 78K.

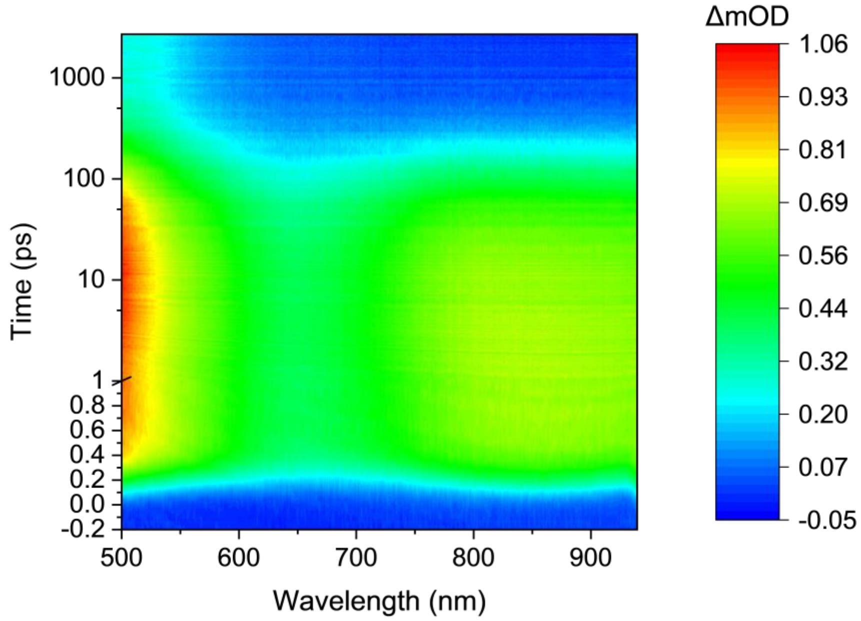

Fig. S6 The psTA spectrum of L^2 (MeOH, 295K)

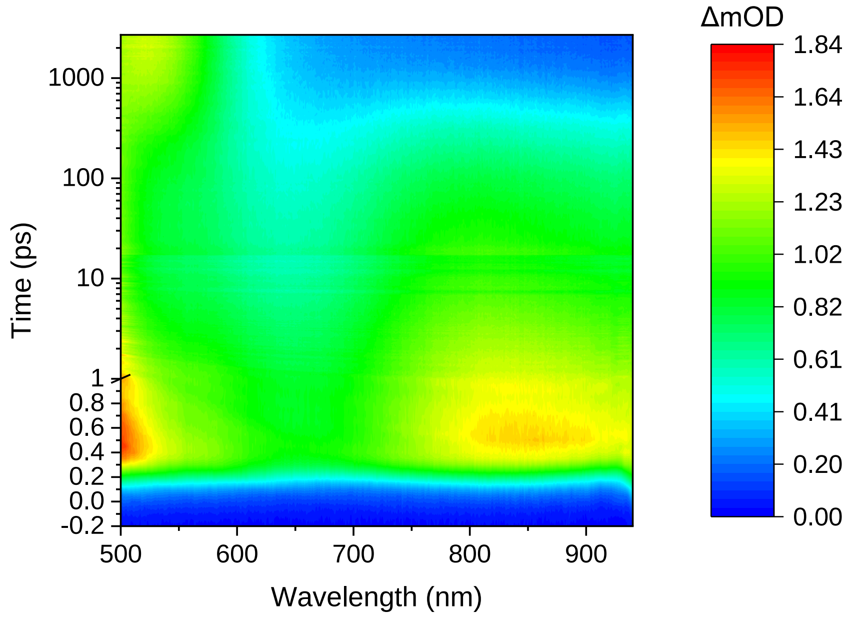

Fig. S7 The psTA spectra of [GdL^2] (MeOH/EtOH 1:4; 295K)

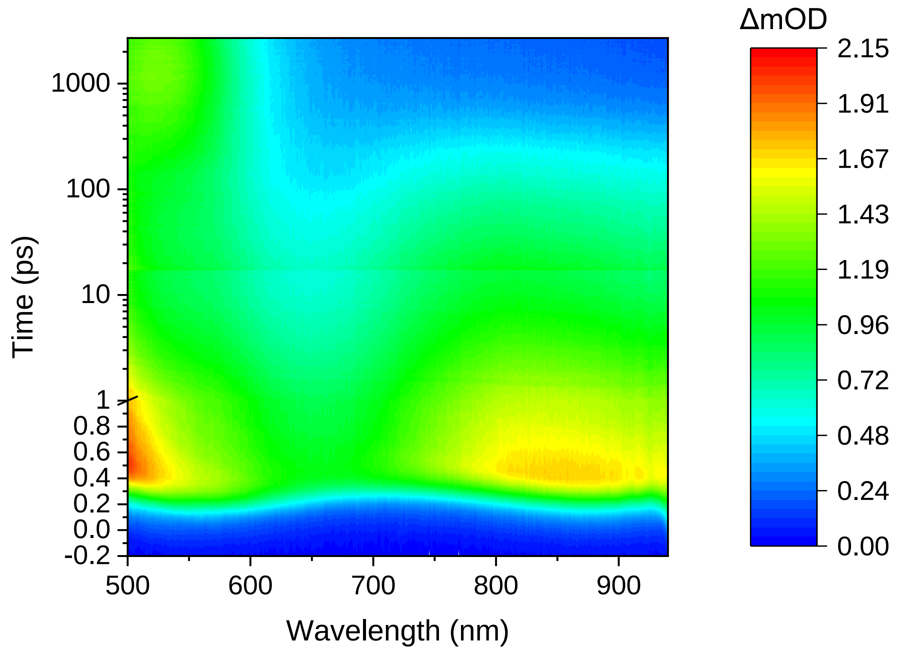

Fig. S8 The psTA spectra of [TbL^2], (MeOH/EtOH 1:4; 295K).

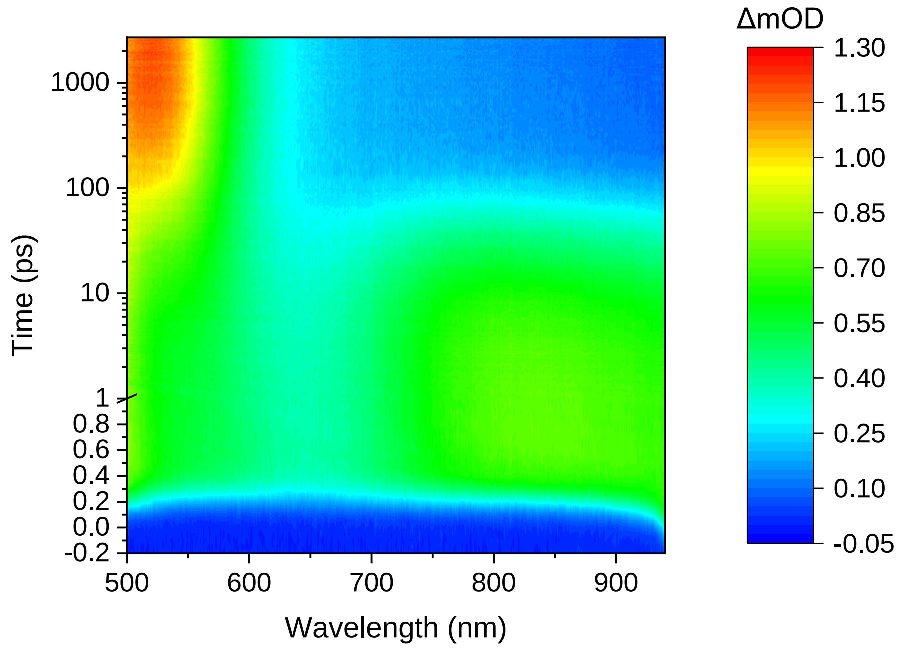

Fig. S9 The psTA spectra of [EuL^2], (MeOH/EtOH 1:4; 295K).

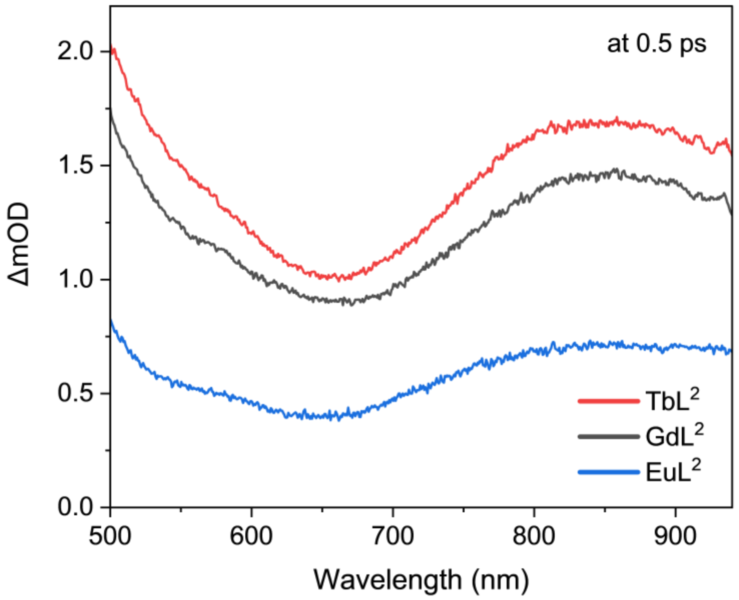

Fig. S10 The ESA signal from excited singlet state at 0.5 ps of [LnL^2]: Ln = Eu, Gd, Tb.

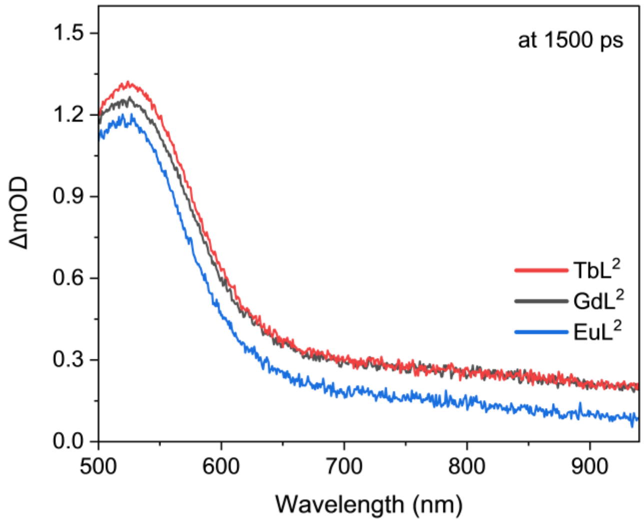

Fig. S11 The ESA signal from excited triplet state at 1500 ps of [LnL^2]: Ln = Eu, Gd, Tb.

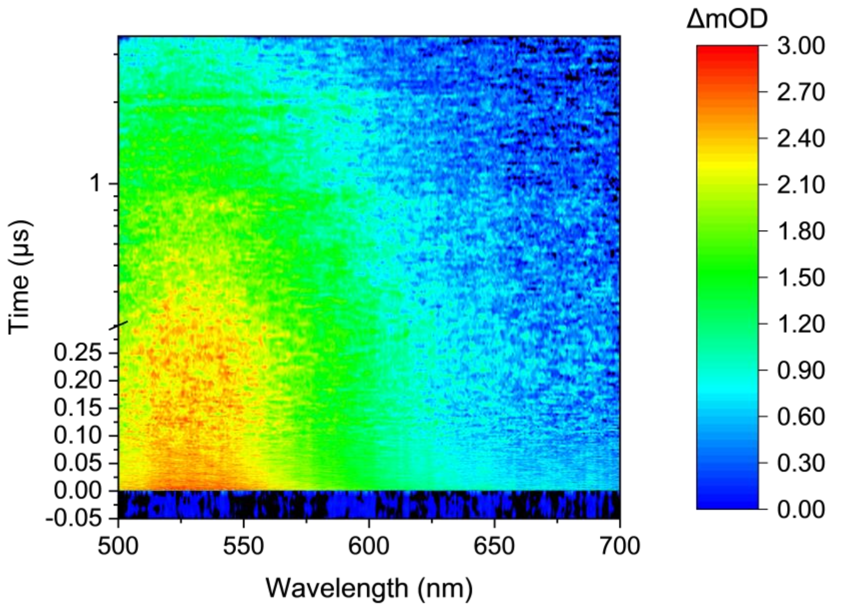

Fig. S12 The nsTA spectra of [GdL^2] (MeOH, 295K)

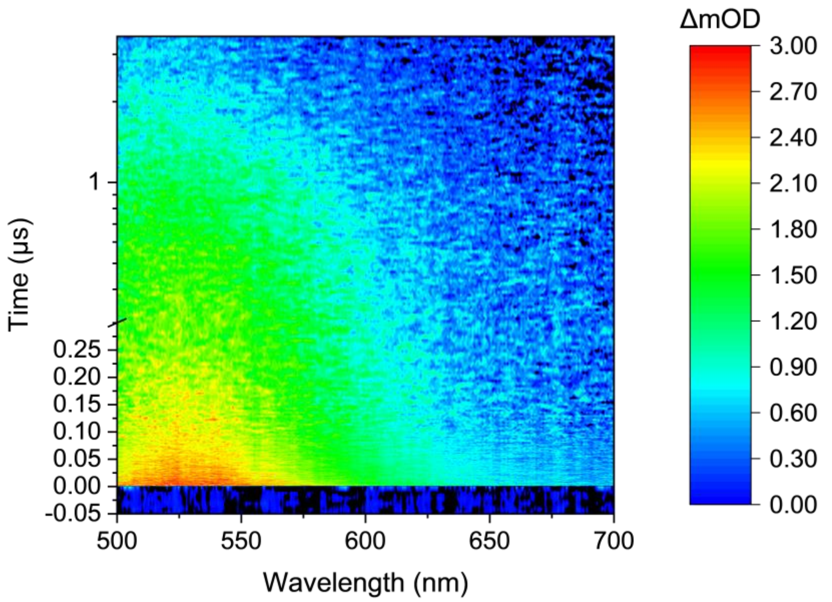

Fig. S13 The nsTA spectra of [TbL^2] (MeOH, 295K).

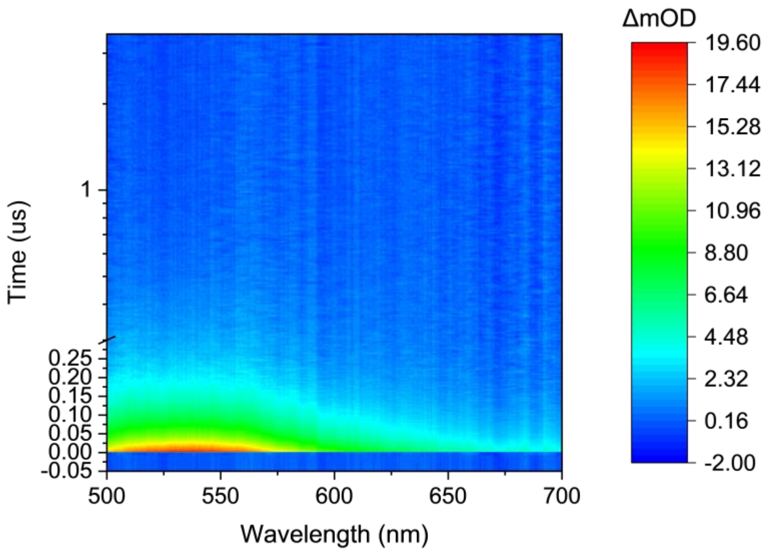

Fig. S14 The nsTA spectra of [EuL^2] (MeOH, 295K).

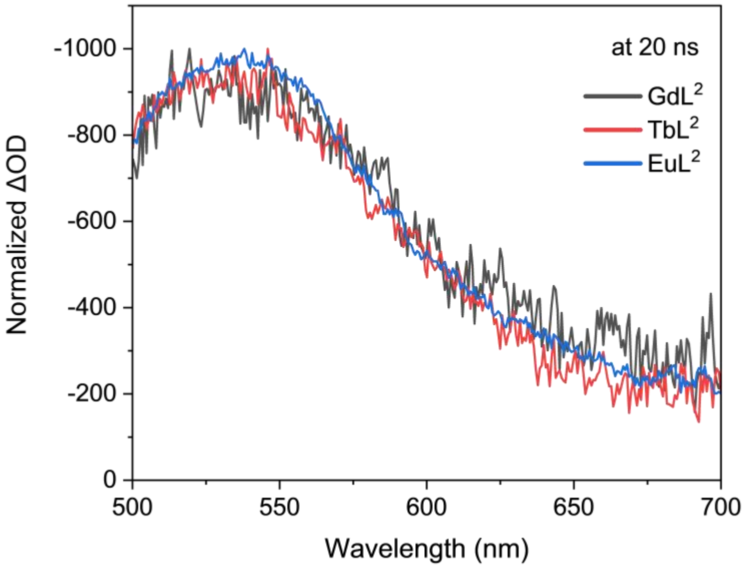

Fig. S15 ESA signals from the excited triplet state at 20 ns for [TbL^2], [GdL^2] and [EuL^2].

Terbium complex quenching behaviour : *T* and *I* dependence

The ionic strength dependence of the emission intensity and lifetime changes (Fig. 7) for [TbL^3]$^+$ were measured under standard conditions (11 μM complex, pH 7.4, 0.1 M HEPES) in the presence of a fixed concentration of added quencher corresponding to half of the apparent K_{SV}^{-1} value. In the presence of 5 mM urate, the emission intensity increased by 20%. An increase in NaCl concentration disfavours quenching, owing to the classical kinetic salt effect. In the presence of (3 mM) iodide, the emission intensity increased by 30 %. With

added ascorbate (2 mM), changes were much more pronounced, and the emission intensity increased more steeply up to 45 %, following the addition of 0.8 M NaCl. In a control experiment in the absence of added quencher, the emission intensity increased by 10%. Intriguingly, quenching of the Tb excited state by ascorbate is significantly more sensitive to ionic strength variation.

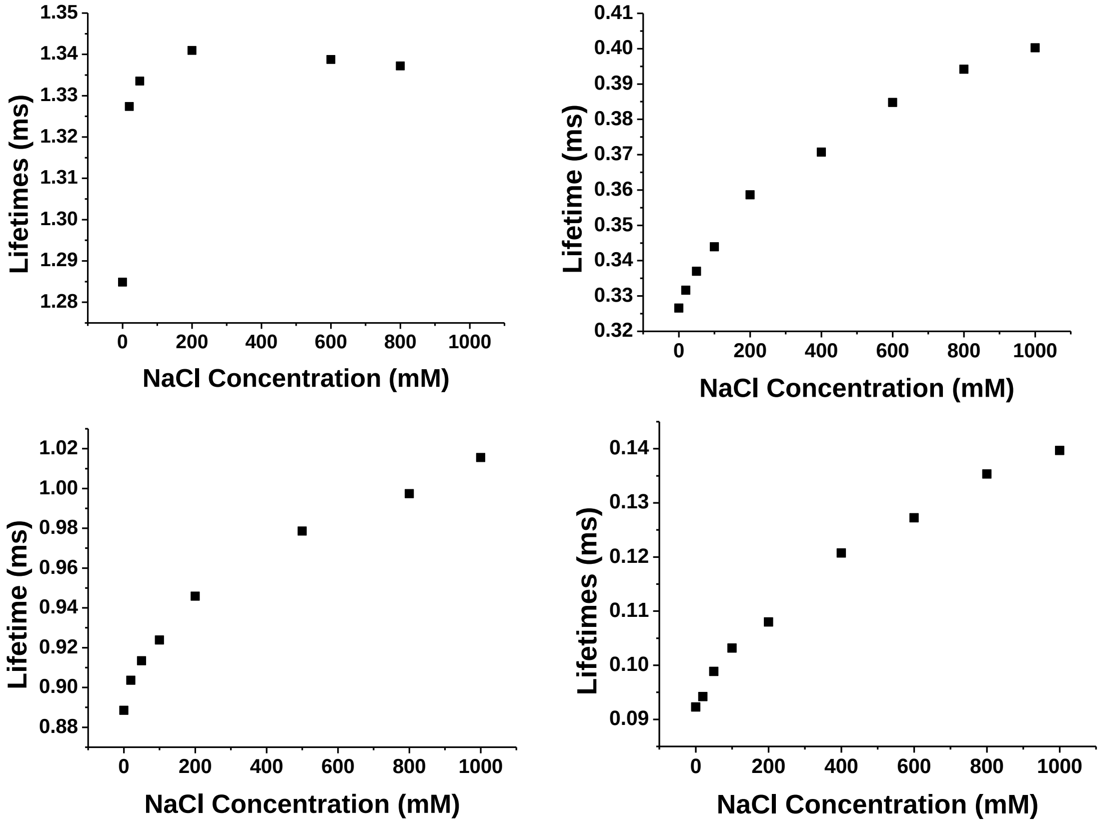

Fig. S16 Ionic strength dependence of the lifetime change for $[TbL^3]^+$ (11 μM, pH 7.4, 0.1 M HEPES): (upper left)) in the absence of quenchers ; (upper right) in the presence of 5 μM urate; (lower left)) 3 mM added iodide ; (lower right) 2 mM added ascorbate.

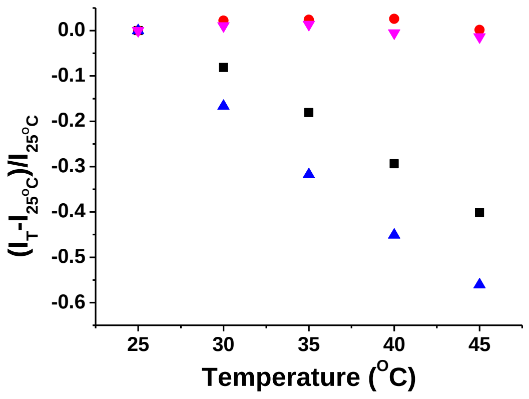

Fig. S17 Temperature dependence of the emission intensity change for $[TbL^3]^+$ (11 μM complex, pH 7.4, 0.1 M HEPES) in the presence of : a) urate (5 μM, *red*); b) iodide (3 mM, *blue*); c) ascorbate (2 mM, *pink*) ; d) in the absence of added quencher, (*black*).

Experiments were also undertaken to explore the sensitivity of $[TbL^3]^+$ emission to temperature variation in the presence of different quenchers. In the presence of urate and ascorbate, no significant changes in emission intensity were observed as the temperature was varied between 25 and 45 °C. At higher temperatures, exciplex formation with urate and ascorbate is intrinsically disfavoured, so that quenching should become less efficient, leading to an intensity (and lifetime) increase. Presumably, this effect is compensated by the decrease in intensity that was observed in the absence of any added quencher (Fig. 8: black squares). In the presence of iodide, the increase in temperature also led to an emission intensity decrease, contrasting with the behaviour with urate and ascorbate, and consistent with a dominant collision-controlled quenching mechanism, involving diffusional encounter of the complex with iodide.

Selected NMR Spectra

^{1}H NMR spectrum ($CDCl_3$, 400 MHz, 295 K) of 2-methyl-5,6 -dimethyl-dipyridoquinoxaline

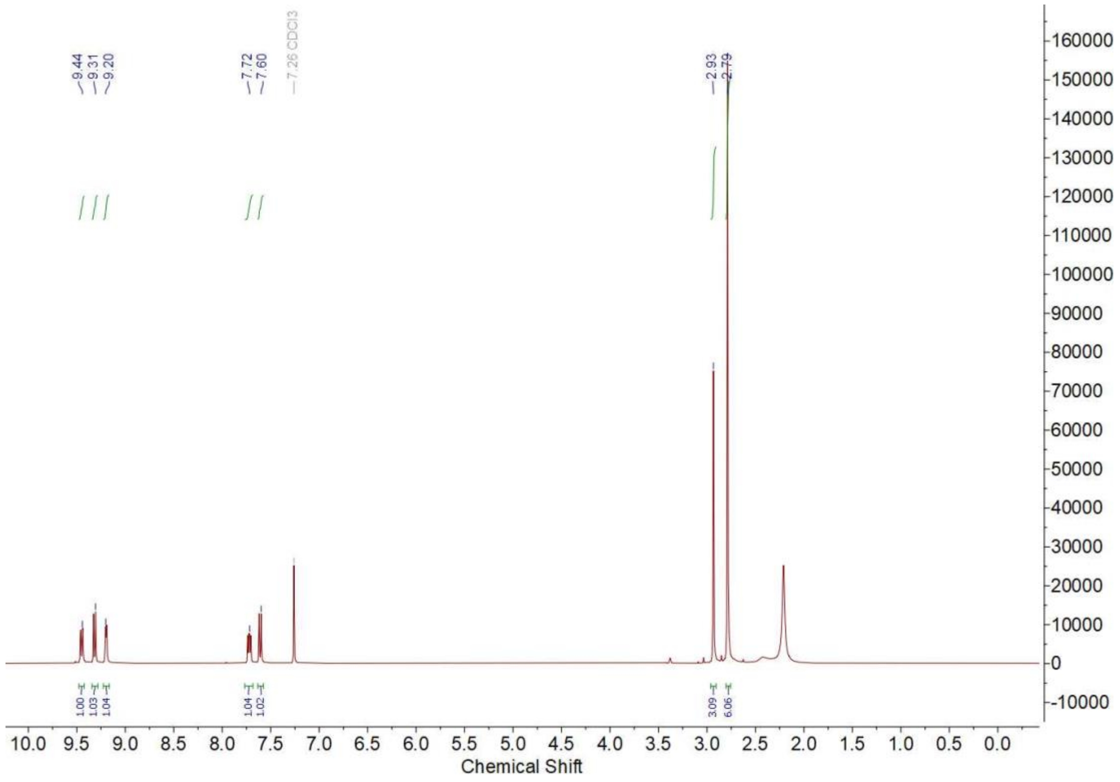

^{13}C NMR spectrum ($CDCl_3$, 101 MHz, 295 K) of 2-methyl-5,6 -dimethyl-dipyridoquinoxaline

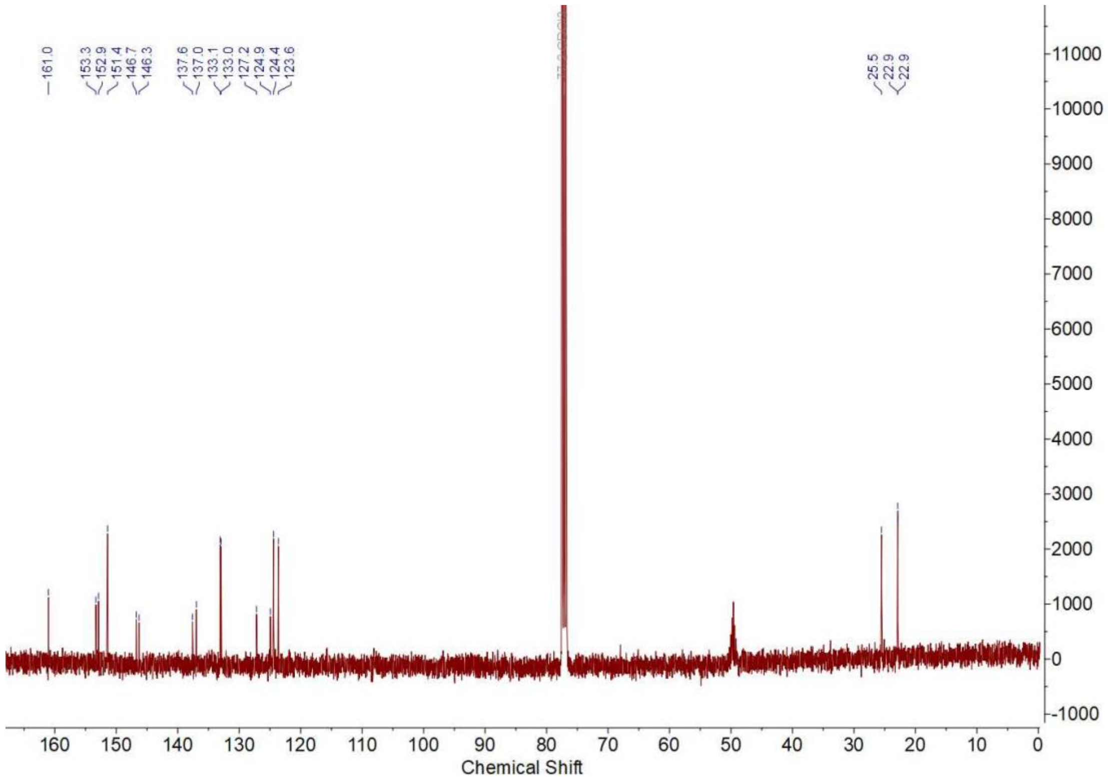

^{1}H NMR spectrum ($CDCl_3$, 400 MHz, 298 K) of 5,6 -dimethyl-dipyridoquinoxaline-2- Carbaldehyde

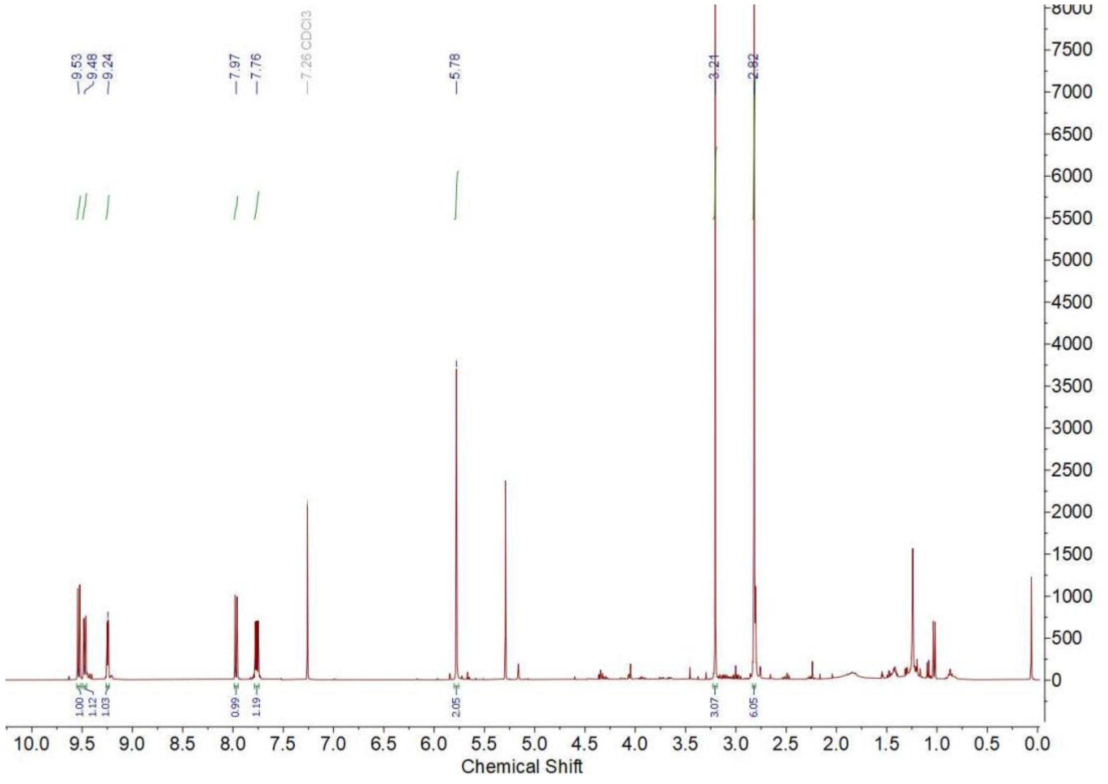

^{1}H NMR spectrum ($CDCl_3$, 400 MHz, 298 K) of tris(t-butyl) ester of L^1

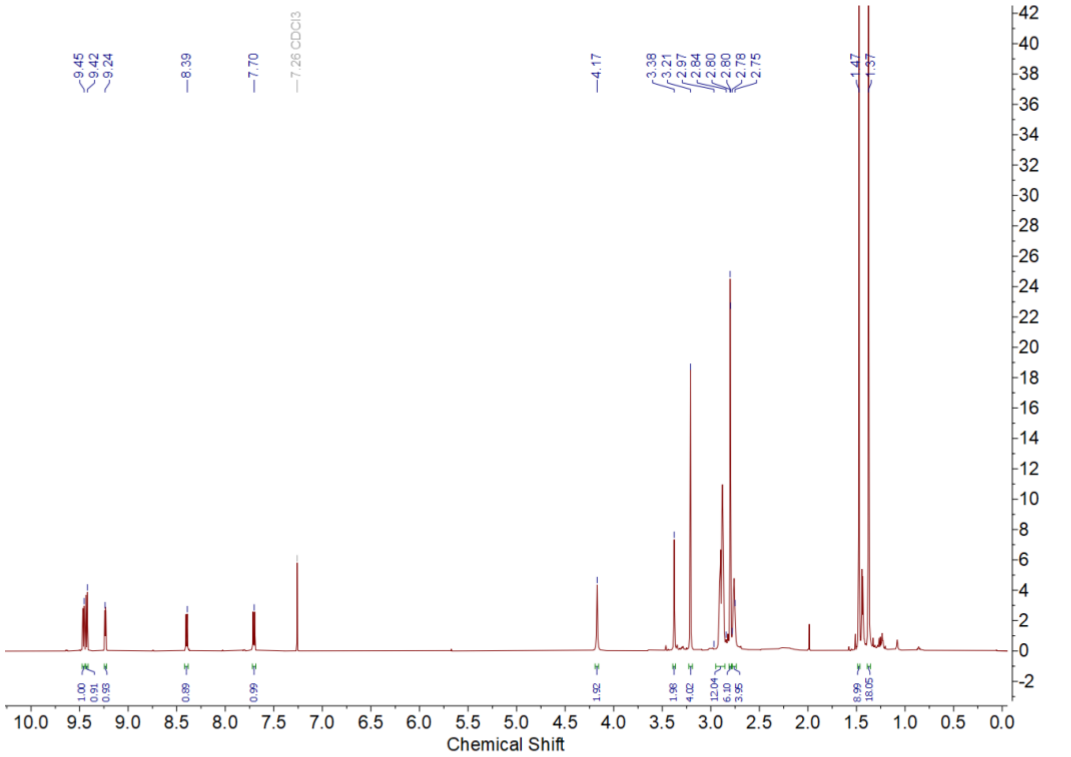

^{13}C NMR spectrum ($CDCl_3$, 101 MHz, 298 K) of tris(t-butyl) ester of L^1

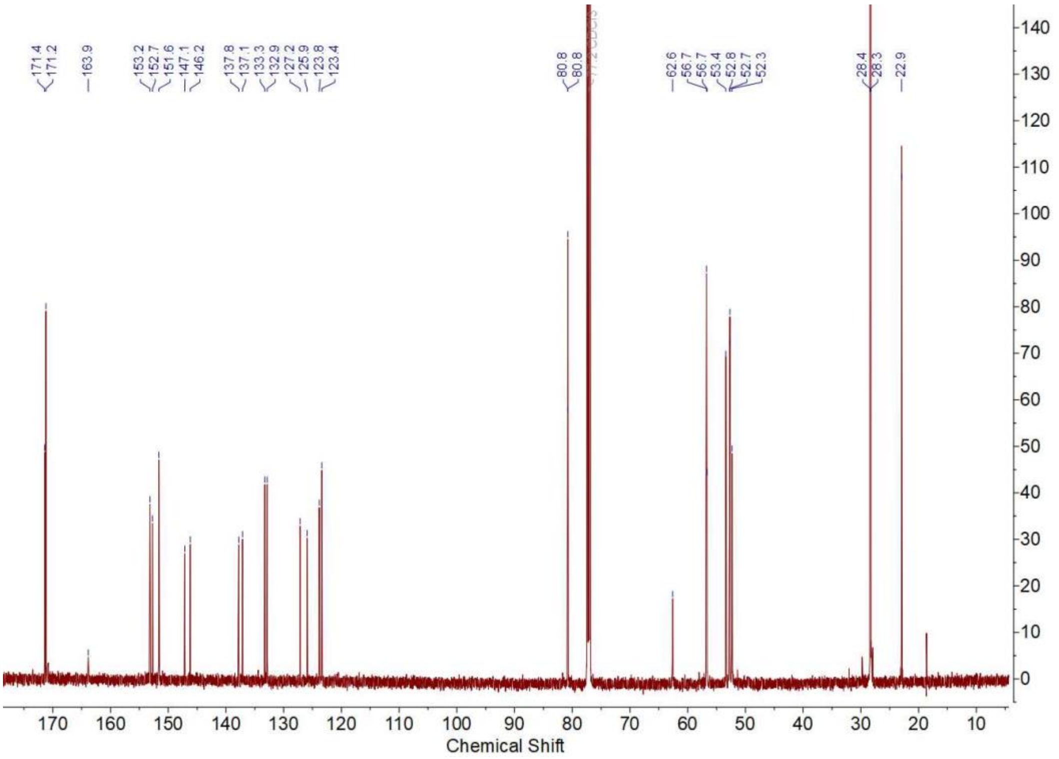

^{1}H NMR spectrum ($CDCl_3$, 400 MHz, 298 K) of Ligand $[H_6L^1]^{3+}$

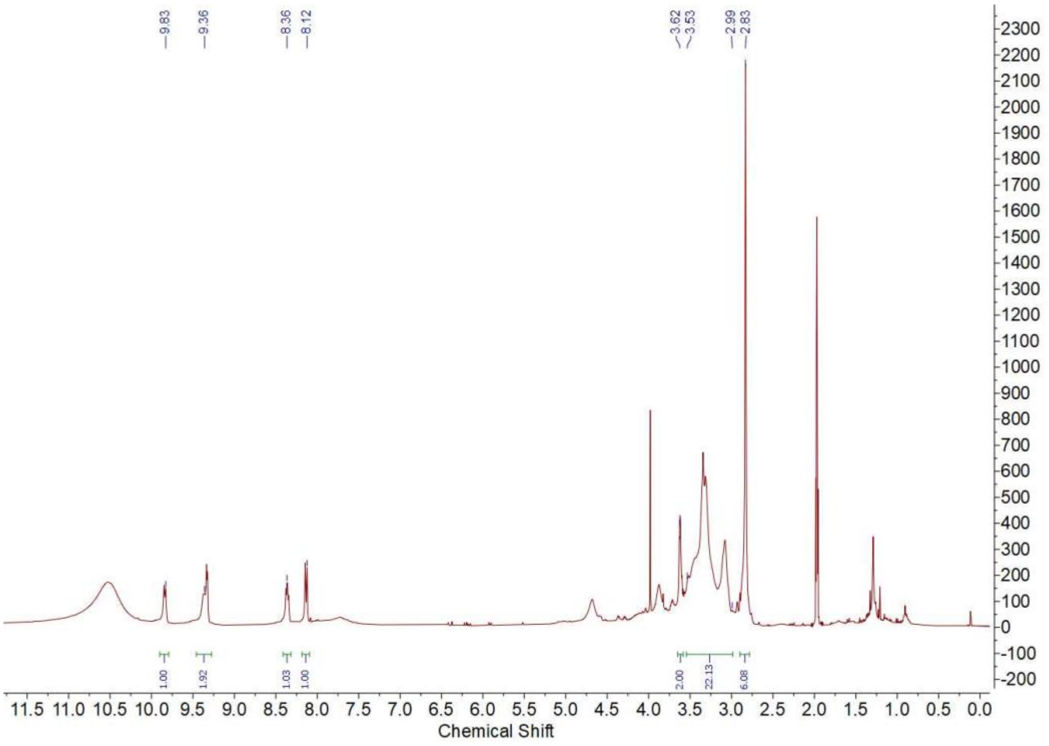

^{1}H NMR spectrum ($CDCl_3$, 400 MHz, 298 K) of 2-methyl-5,6-diphenyl-dipyrridoquinoxaline

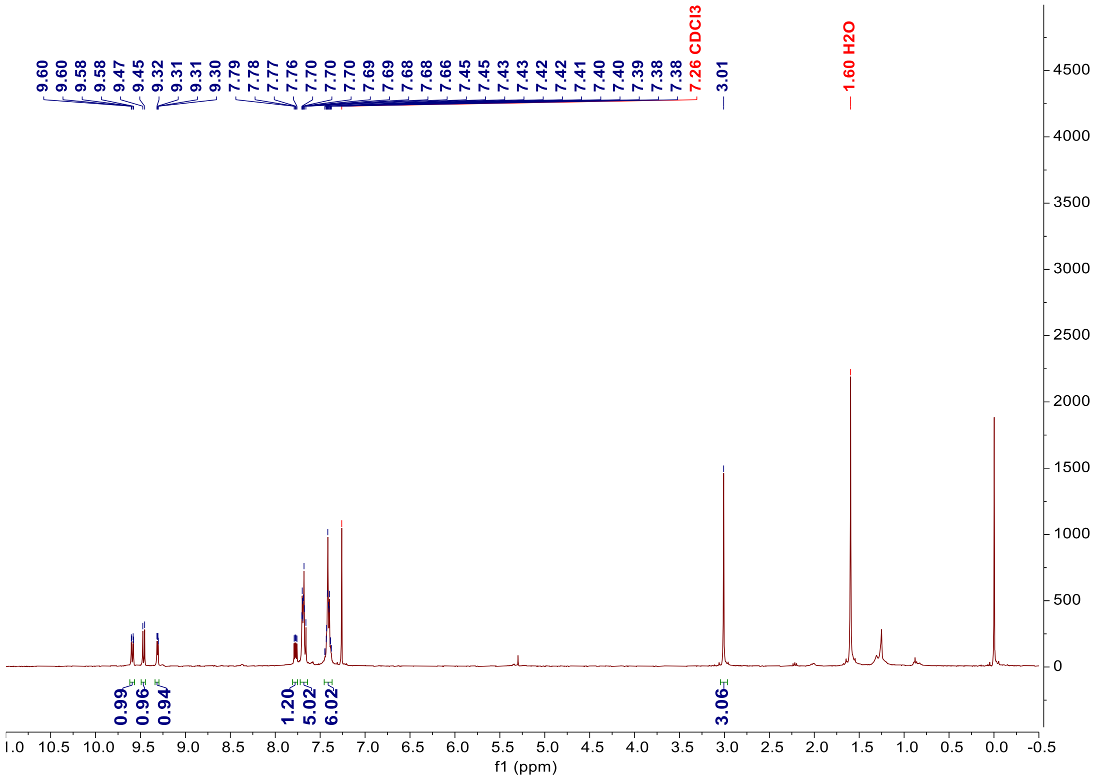

^{13}C NMR spectrum ($CDCl_3$, 101 MHz, 298 K) of 2-methyl-5,6-diphenyl-dipyrridoquinoxaline

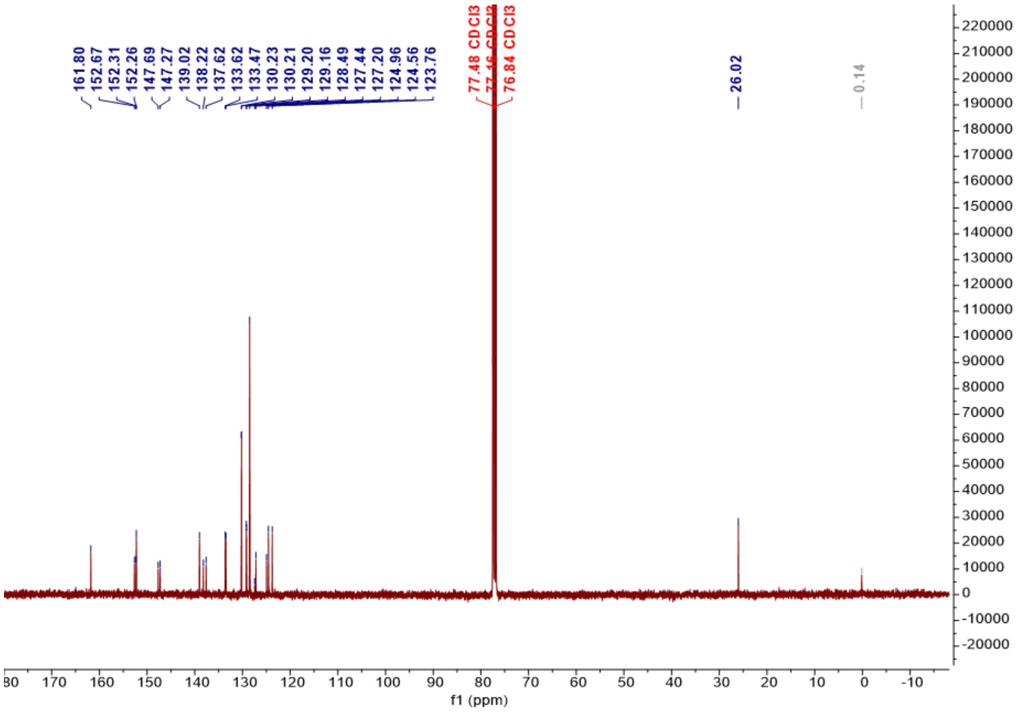

^{1}H NMR spectrum ($CDCl_3$, 400 MHz, 298 K) of 2-methanoyl-5,6-diphenyl-dipyridoquinoxaline

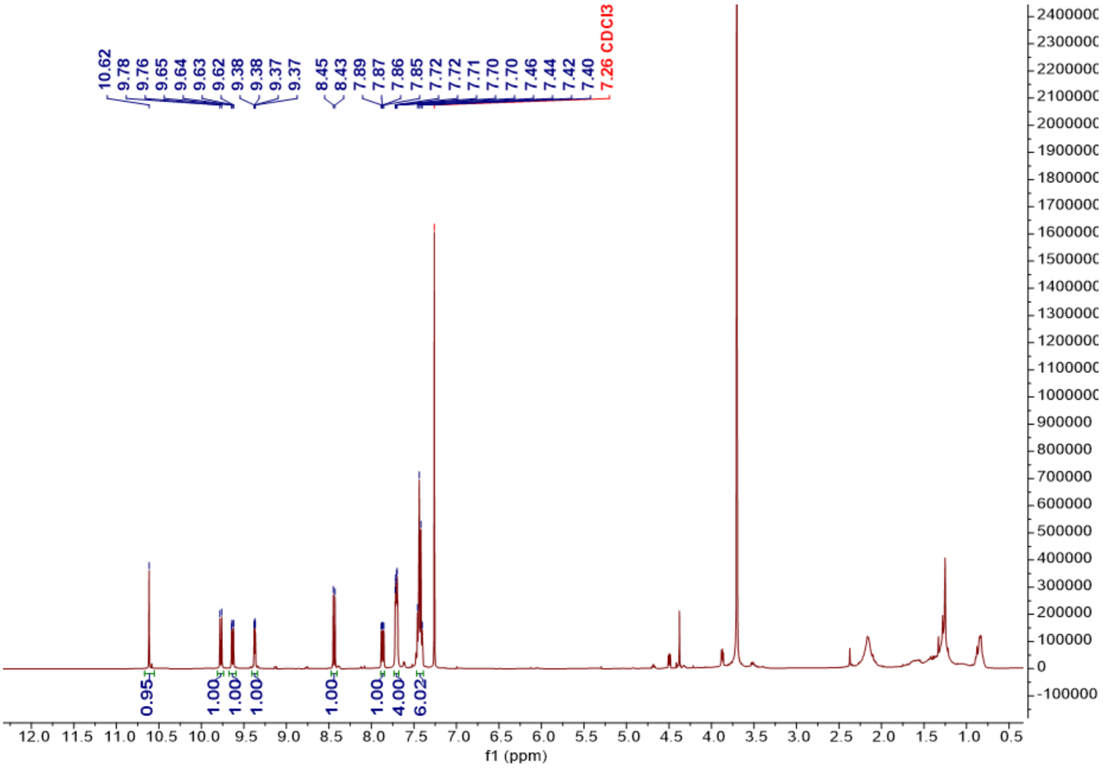

^{13}C NMR spectrum ($CDCl_3$, 101 MHz, 298 K) of 2-methanoyl-5,6-diphenyl-dipyridoquinoxaline

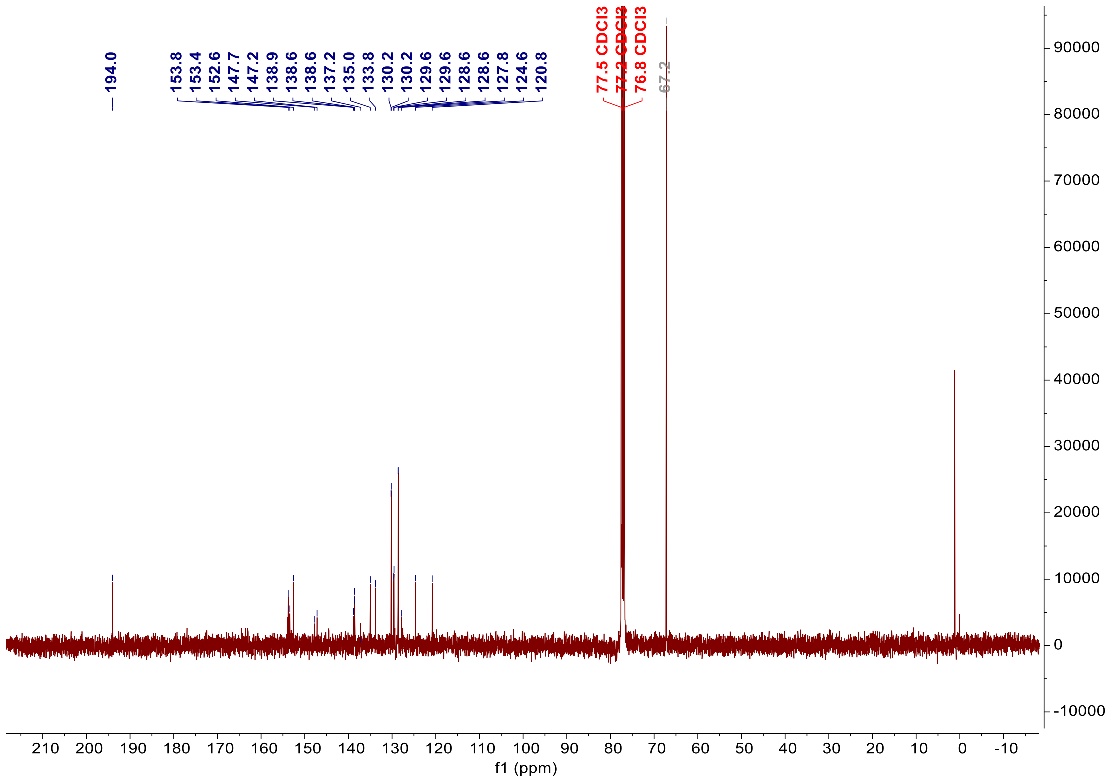

^{1}H NMR spectrum ($CDCl_3$, 400 MHz, 298 K) of 2-hydroxymethyl -7,8 -diphenyl-dipyridoquinoxaline

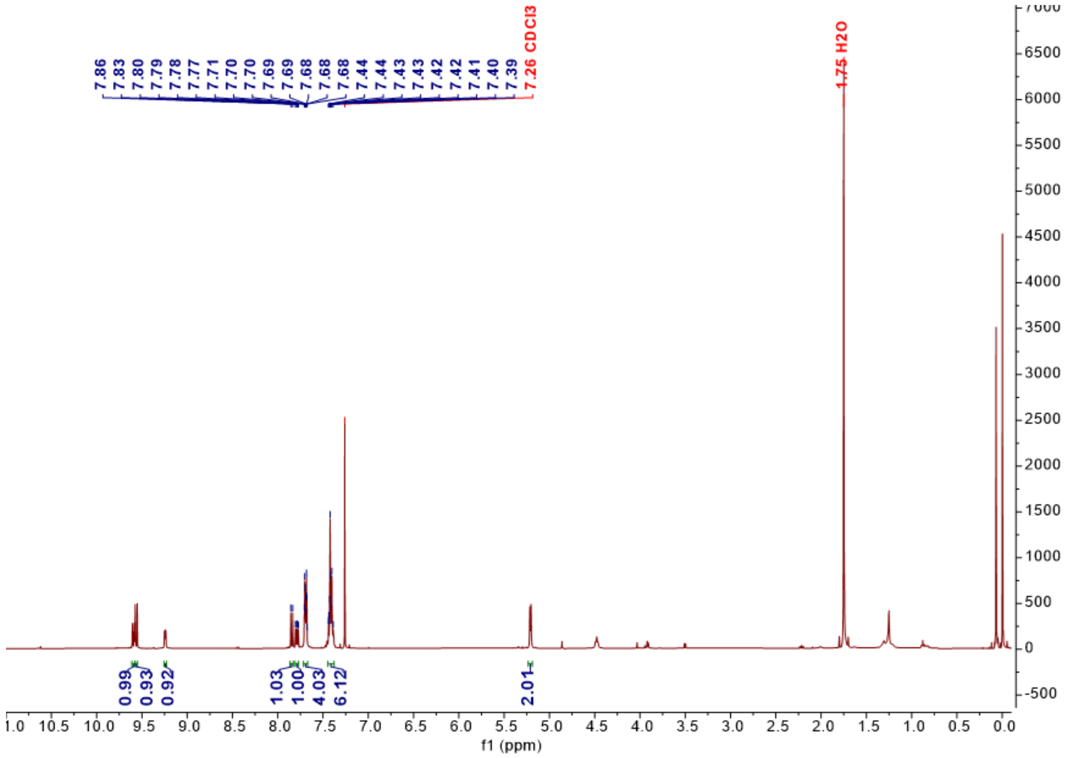

^{13}C NMR spectrum ($CDCl_3$, 101 MHz, 298 K) of 2-hydroxymethyl -7,8 -diphenyl-dipyridoquinoxaline

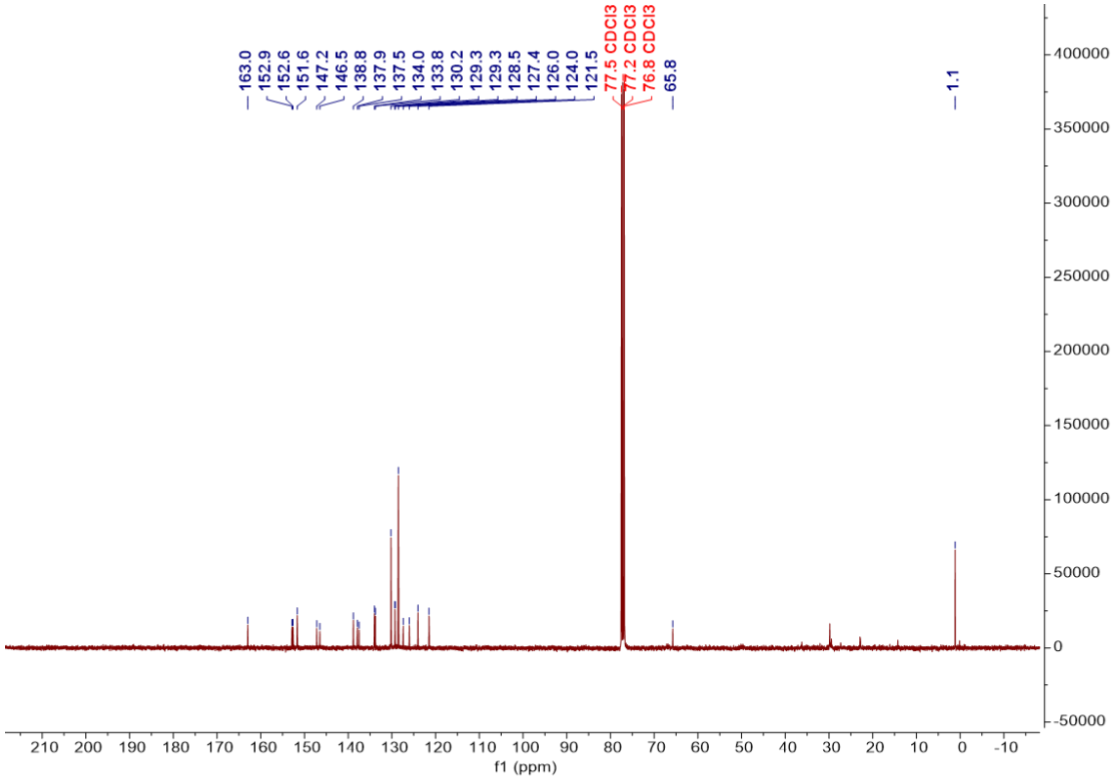

^{1}H NMR spectrum ($CDCl_3$, 400 MHz, 298 K) of 2-methanesulfanatomethyl -5,6 -diphenyl-dipyridoquinoxaline

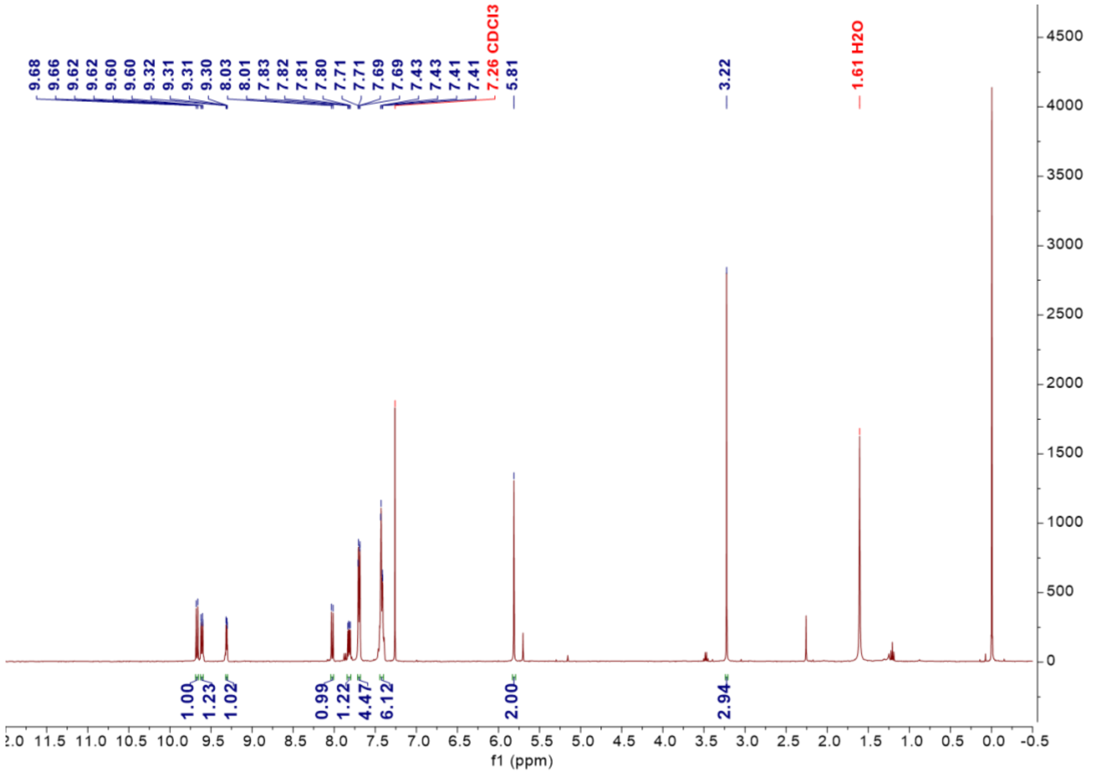

^{1}H NMR spectrum ($CDCl_3$, 400 MHz, 298 K) of tris(t-butyl) ester of L^2

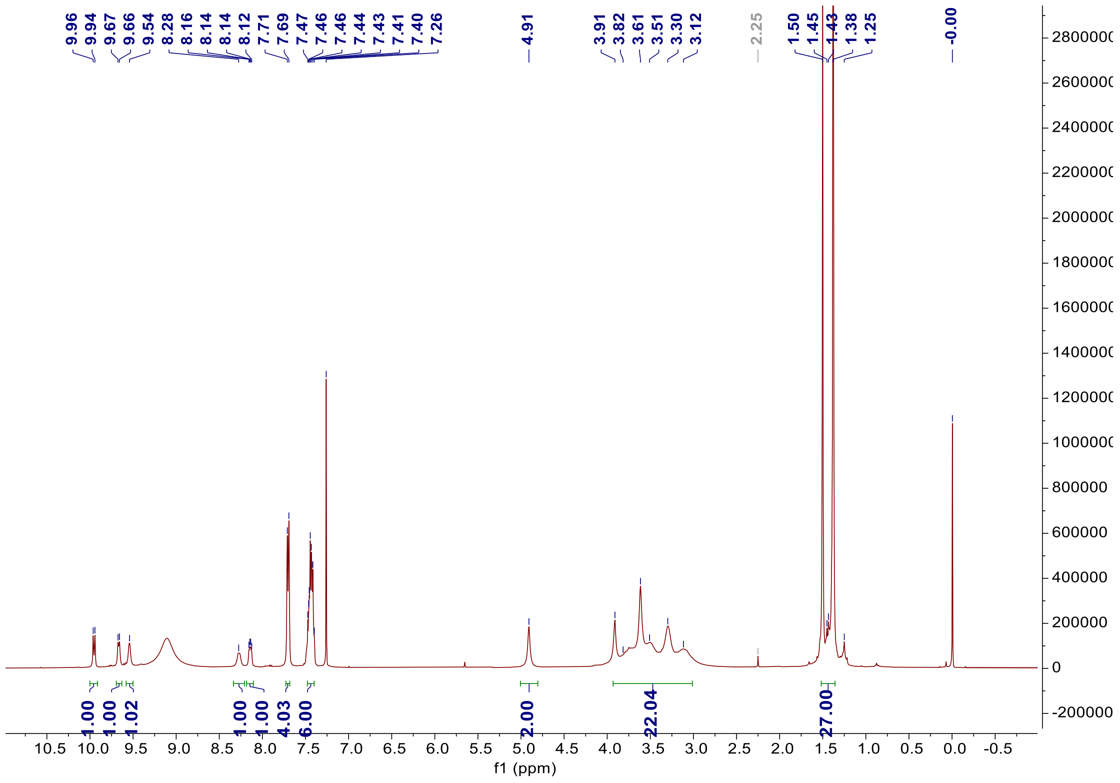

^{13}C NMR spectrum ($CDCl_3$, 101 MHz, 298 K) of tris(t-butyl) ester of L^2

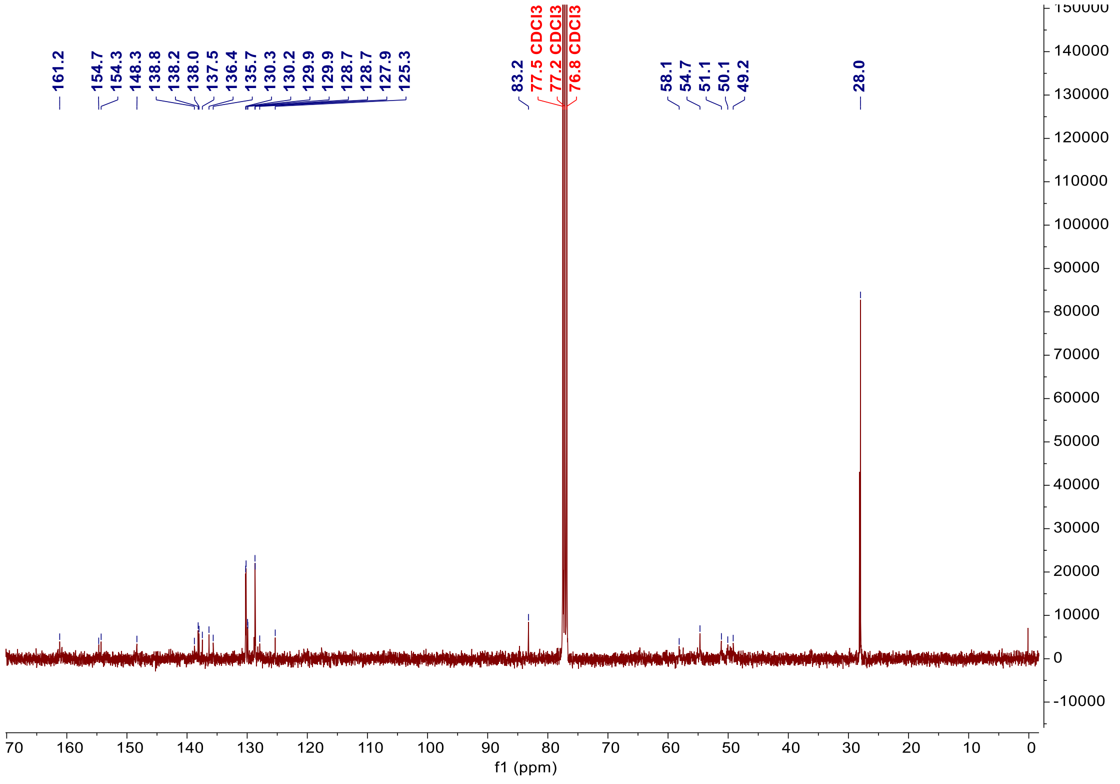

^{1}H NMR spectrum ($CDCl_3$, 400 MHz, 298 K) of dimethyl 6,6'-[(7-(tert-

butoxycarbonyl)-1,4,7-triazacyclononane-1,4-diyl) bis(methylene)]dipicolinate

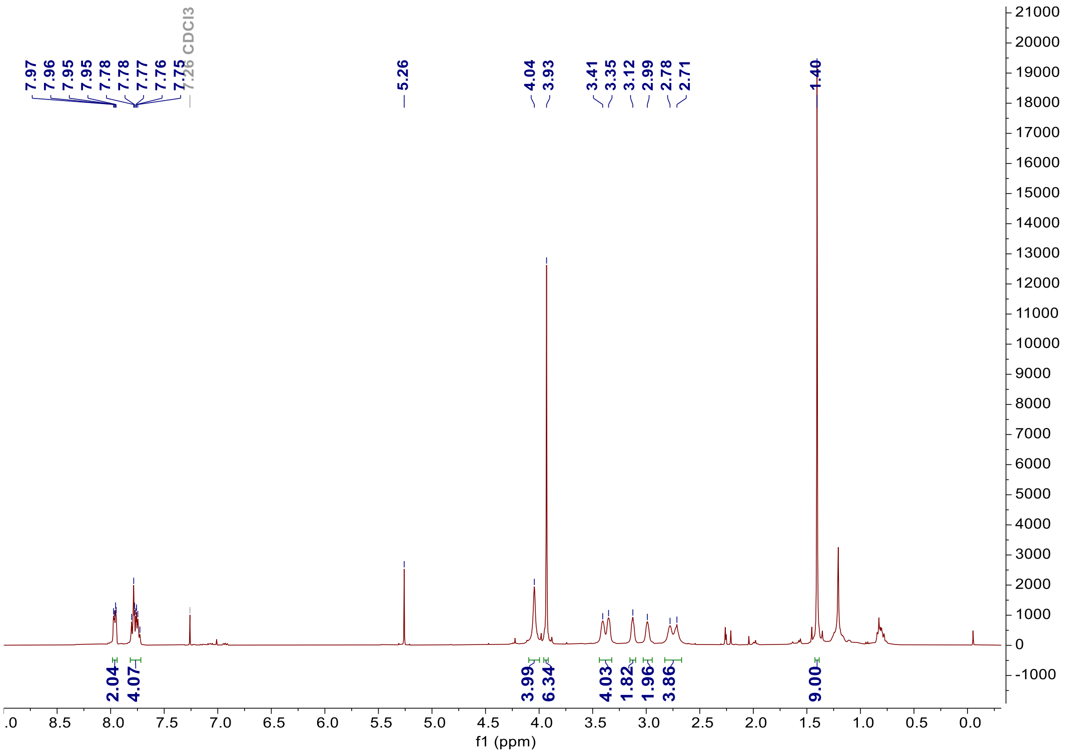

^{13}C NMR spectrum ($CDCl_3$, 101 MHz, 298 K) of dimethyl 6,6'-[(7-(tert-butoxycarbonyl)-1,4,7-triazacyclononane-1,4-diyl) bis(methylene)]dipicolinate

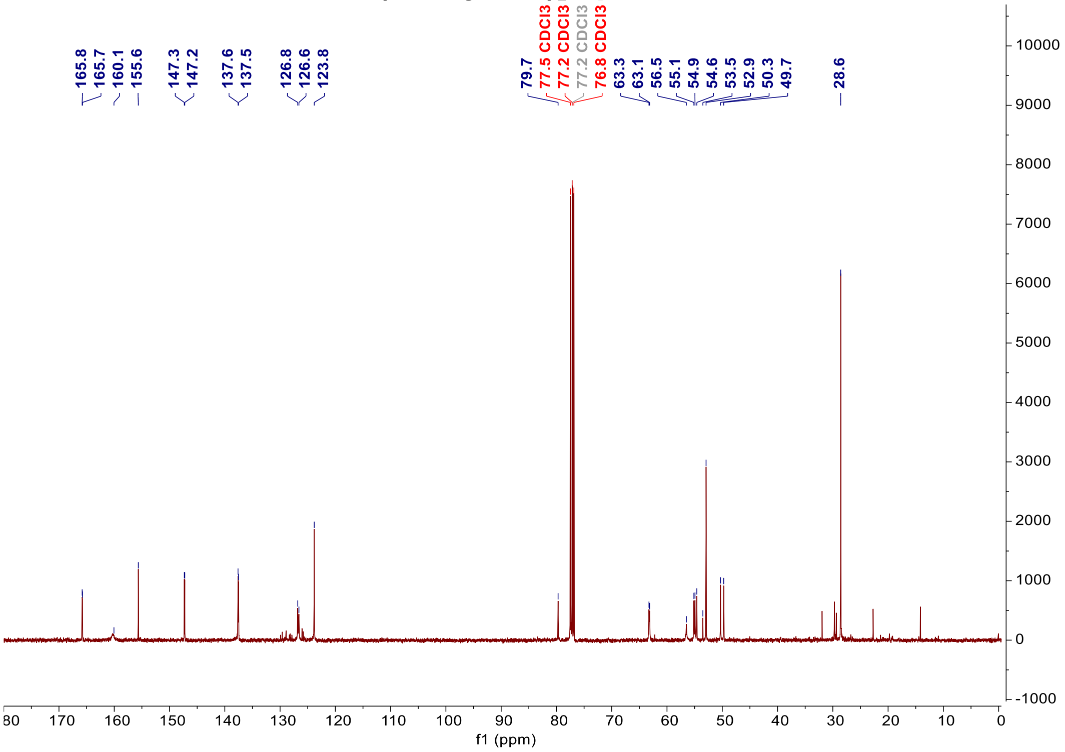

^{1}H NMR spectrum ($CDCl_3$, 400 MHz, 298 K) of dimethyl-6,6'-((1,4,7-triazacyclononane-1,4-diyl) bis(methylene))dipicolinate

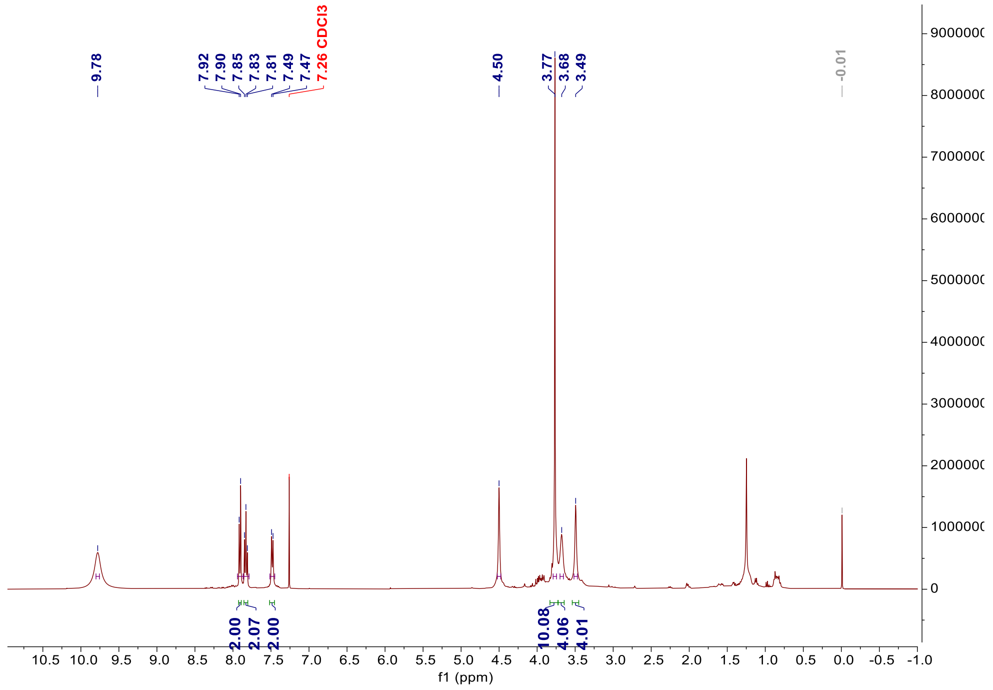

^{13}C NMR spectrum ($CDCl_3$, 101 MHz, 298 K) of dimethyl-6,6'-((1,4,7-triazacyclononane-1,4-diyl) bis(methylene))dipicolinate

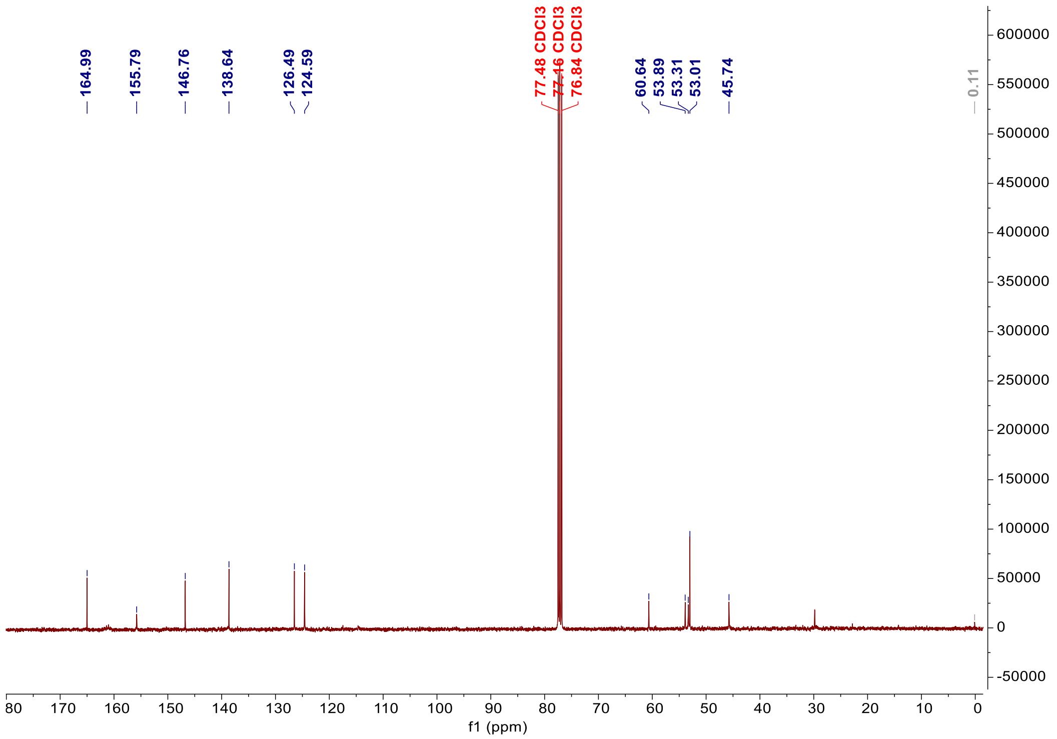

^{1}H NMR spectrum ($CDCl_3$, 400 MHz, 298 K) of L^3

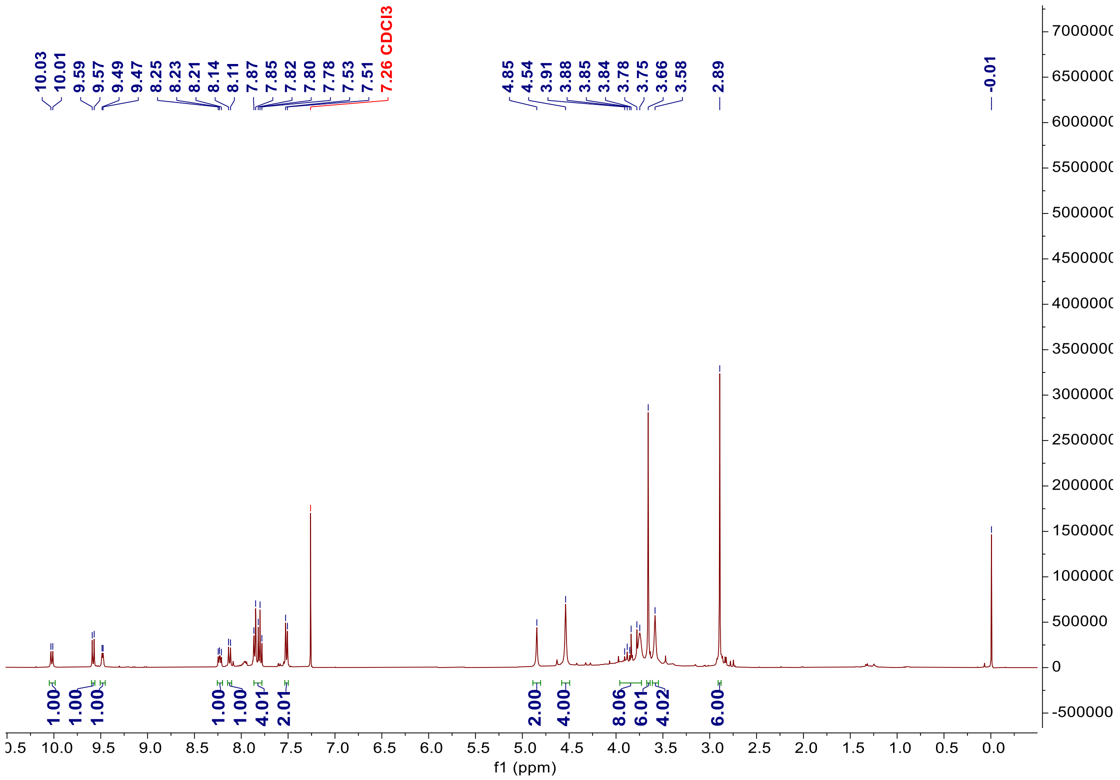

^{13}C NMR spectrum ($CDCl_3$, 101 MHz, 298 K) of L^3

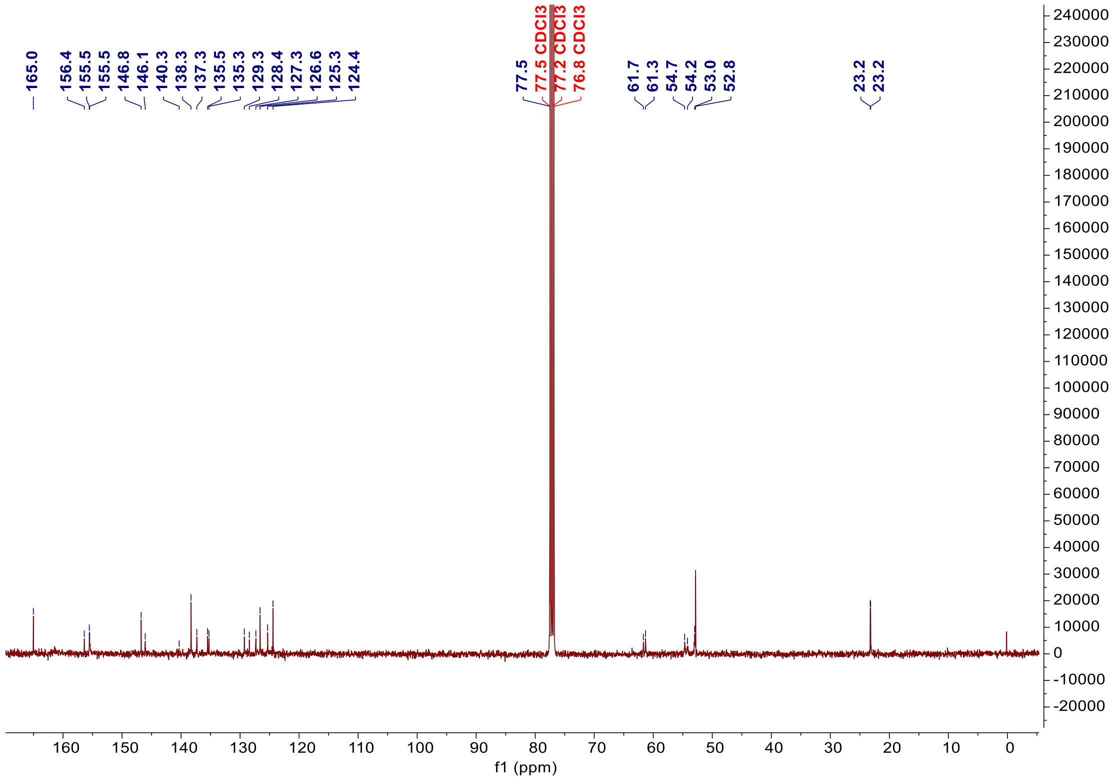

^1H NMR spectrum ($CDCl_3$, 400 MHz, 298 K) of L^4

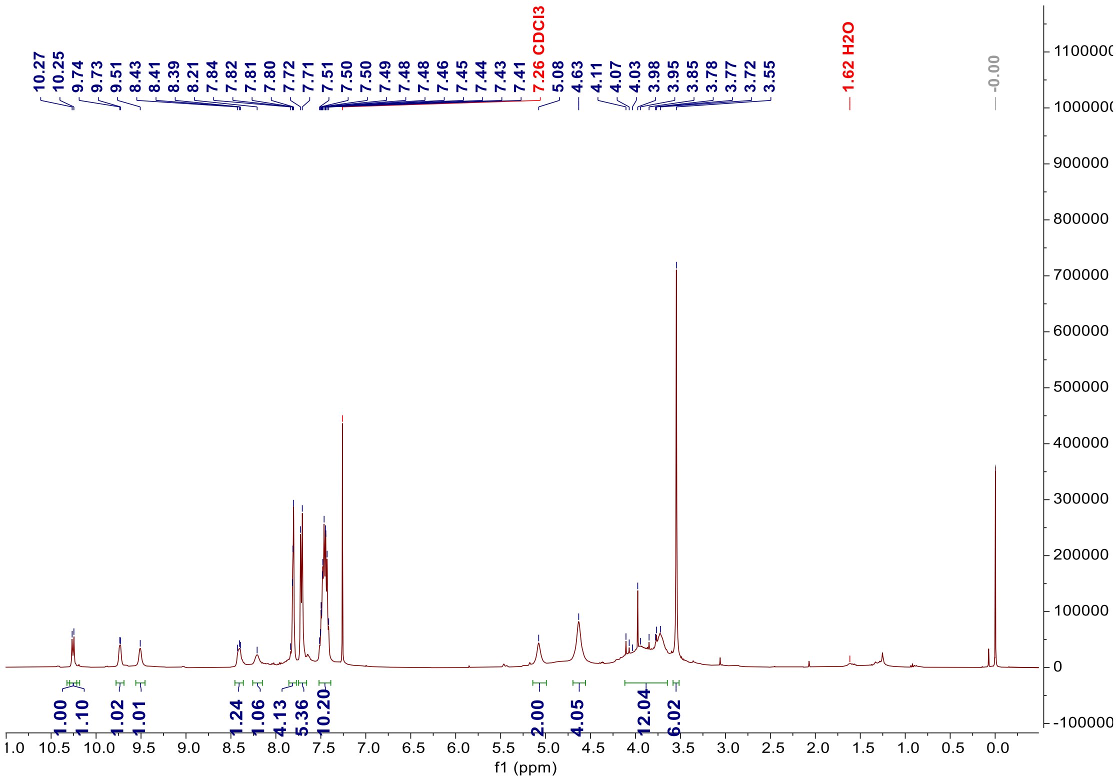

^{13}C NMR spectrum (CDCl$_3$, 101 MHz, 298 K) of L^4

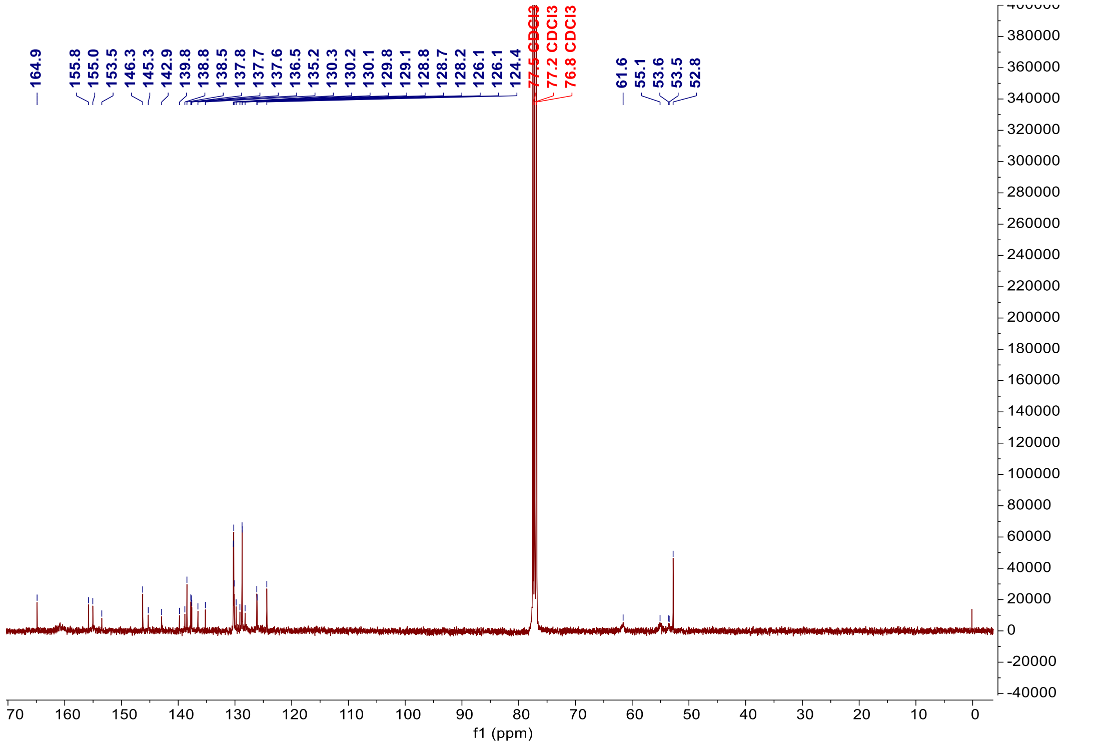

^{1}H NMR spectrum (CDCl$_3$, 400 MHz, 298 K) of L^5

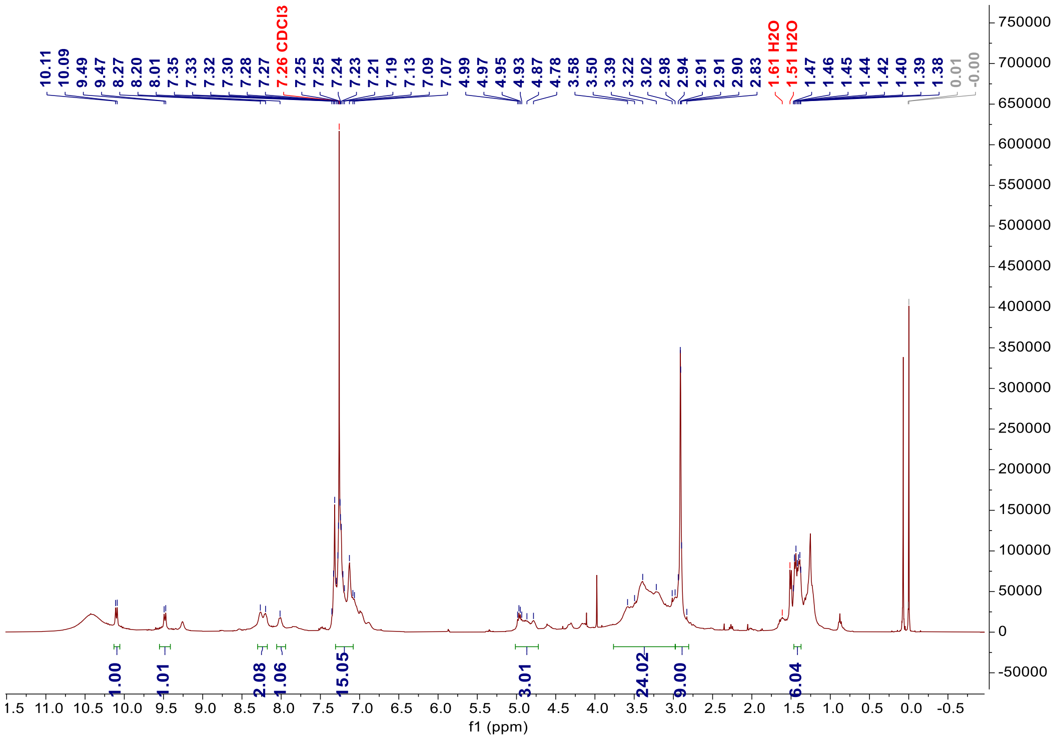

^{13}C NMR spectrum (CDCl₃, 151 MHz, 298 K) of L[5]

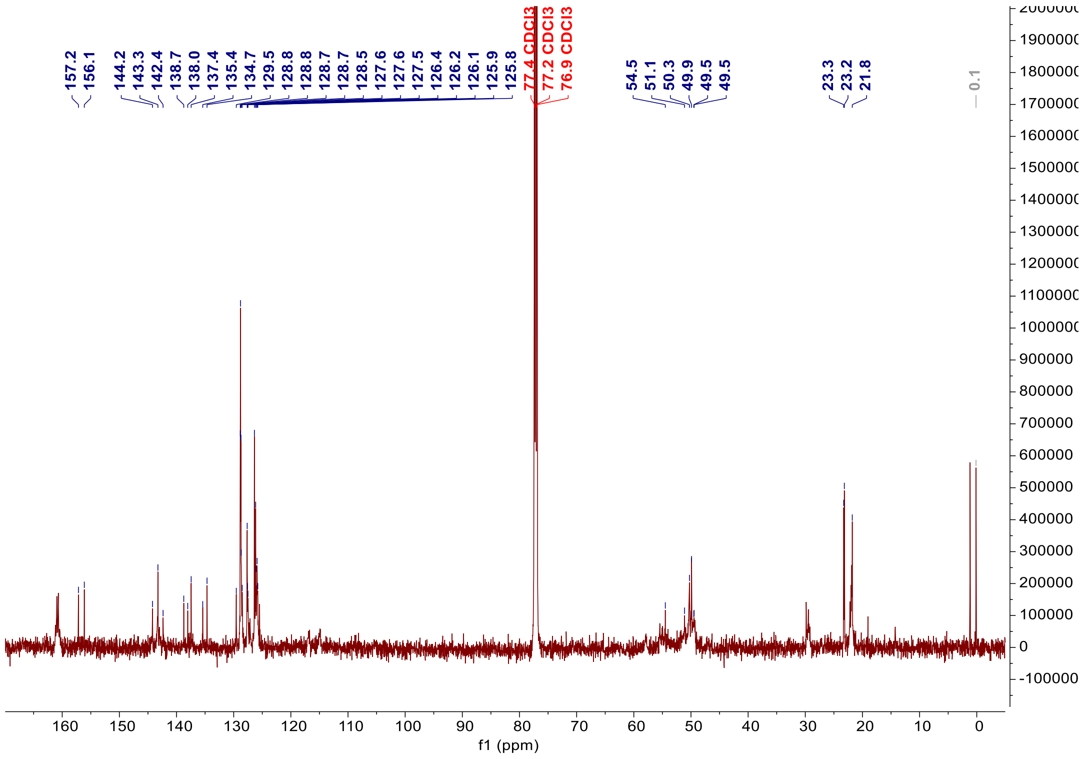

^{1}H NMR spectrum (CDCl₃, 400 MHz, 298 K) of L[6]

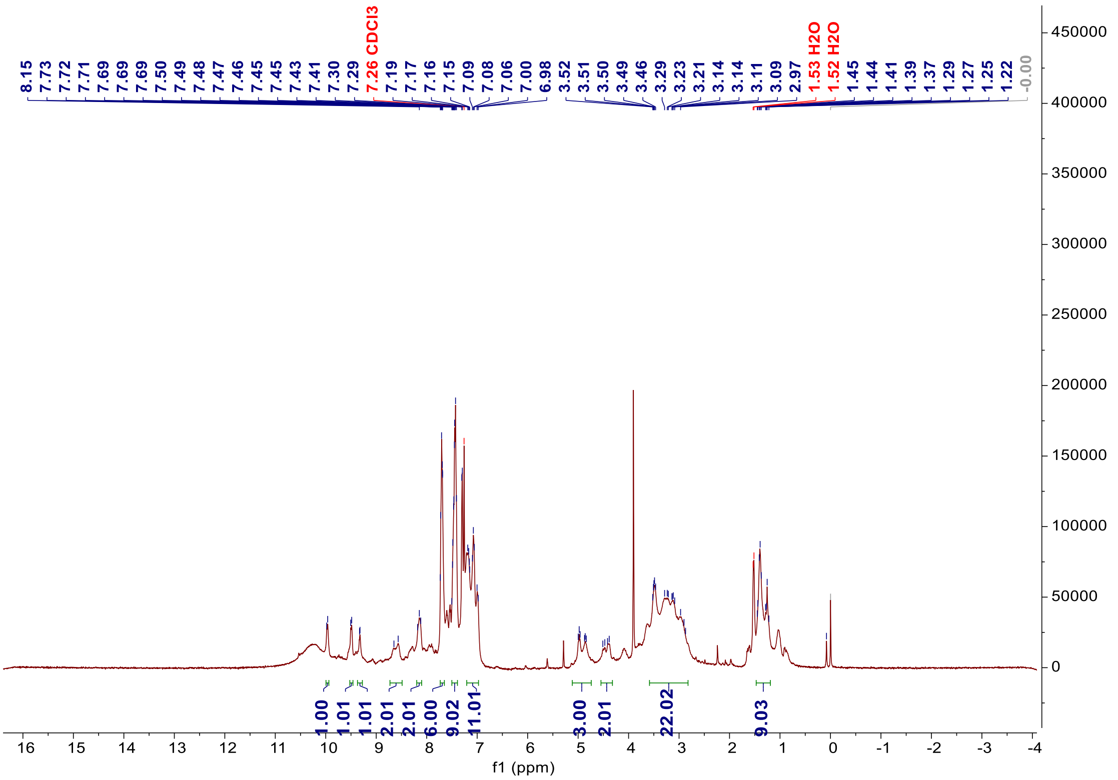

^{13}C NMR spectrum ($CDCl_3$, 151 MHz, 298 K) of L^6

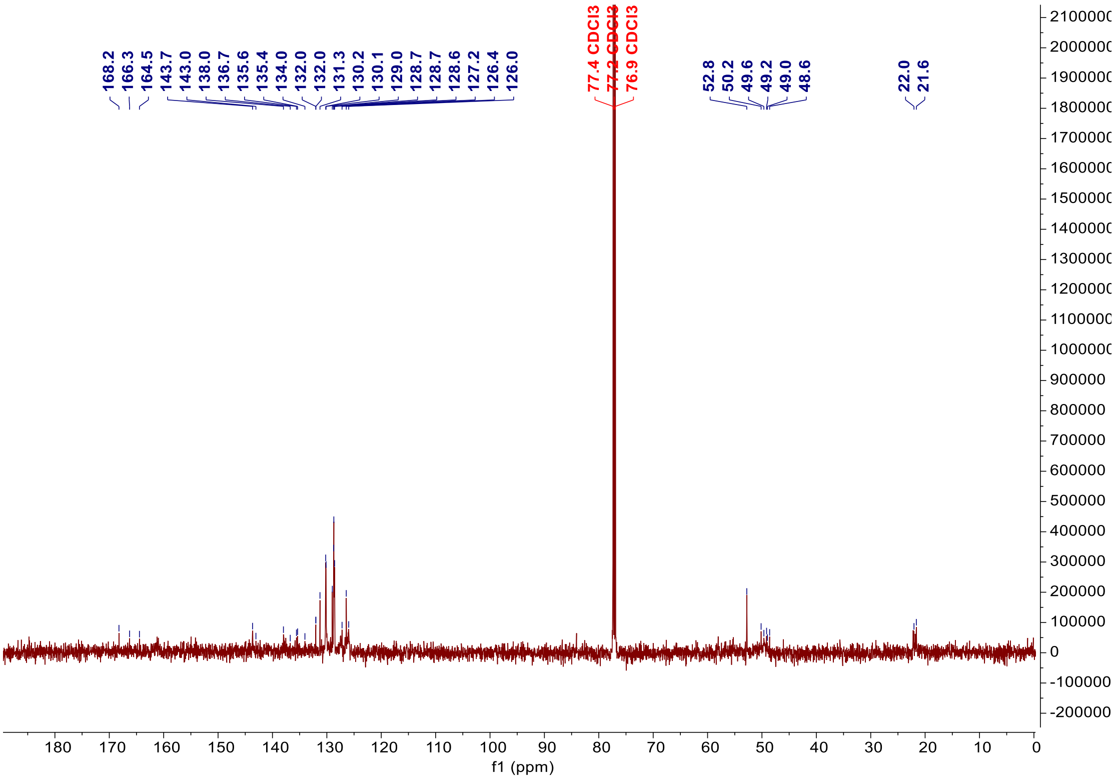

References:

1. Zheng, R. H.; Guo, H. C.; Jiang, H. J.; Xu, K. H.; Liu, B. B.; Sun, W. L.; Shen, Z. Q., *Chinese Chemical Letters,* **2010**, 21 (11), 1270-1272.
2. Ciruelos, S.; Doppiu, A.; Englert, U.; Salzer, A., New optically active 'constrained-geometry' cyclopentadienyl-phosphine ligands and their metal complexes. *J. Organomet. Chem*. 2002, 663 (1), 183-191.
3. Chooi, S. Y. M.; Leung, P. H.; Ng, S. C.; Quek, G. H.; Sim, K. Y., A simple route to optically pure 2,3-diaminobutane. *Tetrahedron. Asymmetry*. **1991**, 2 (10), 981-982.
4. Jirkovská, A.; Karabanovich, G.; Kubeš, J.; Skalická, V.; Melnikova, L.; Korábečný, J.; Kučera, T.; Jirkovský, E.; Nováková, L.; Bavlovič Piskáčková, H.; Škoda, J.; Štěrba, M.; Austin, C. A.; Šimůnek, T.; Roh, J. Structure–Activity Relationship Study of Dexrazoxane Analogues Reveals ICRF-193 as the Most Potent Bisdioxopiperazine against Anthracycline Toxicity to Cardiomyocytes Due to Its Strong Topoisomerase IIβ Interactions. *J. Med. Chem.* **2021**, 64 (7), 3997–4019.
5. Kovacs, Z.; Sherry, A. D. A general synthesis of mono- and disubstituted 1,4,7-triazacyclononanes. *Tetrahedron. Lett*. **1995**, 36(51): 9269-9272.
6. Bourdolle, A.; Allali, M.; Mulatier, J.C.; Le Guennic, B.; Zwier, J.M.; Baldeck, P.L.; Bünzli, J. C. G.; Andraud, C.; Lamarque, L.; Maury, O. Modulating the Photophysical Properties of Azamacrocyclic Europium Complexes with Charge-Transfer Antenna Chromophores. *Inorg. Chem.* **2011**, 50, 4987–4999.
7. Vallade, M.; Reddy, P. S.; Fischer, L.; Huc, I.; Enhancing Aromatic Foldamer Helix Dynamics to Probe Interactions with Protein Surfaces. *Eur. J. Org. Chem*., **2018**, 40, 5489-5498.
8. Cho, S. D.; Song, S. Y.; Park, Y. D.; Kim, J. J.; Joo, W. H.; Shiro, M.; Falck, J. R.; Shin, D. S. Novel synthesis of pyridazino[4,5-*b*][1,4]oxazin-3,8-diones. *Tetrahedron. Lett*. **2003**, 44(50): 8995-8998.
9. Mosandl, A. Über synthetische Aromastoffe, 4. Mitt. Diastereomere Amide der 3-Methyl-3-phenylglycidsäure. *Arch. Pharm. Pharm. Med. Chem*., **1984**, 317, 345-352.
10. Burdette, S. C.; Lippard, S. J. The Rhodafluor Family. An Initial Study of Potential Ratiometric Fluorescent Sensors for Zn^{2+}. *Inorg. Chem*. **2002**, 41, 25, 6816–6823.
11. Finney, K. -L. N. A.; Harnden, A. C.; Rogers, N. J.; Senanayake, P. K.; Blamire, A. M.; O'Hogain, D.; Parker, D. Simultaneous Triple Imaging with Two PARASHIFT Probes: Encoding Anatomical, pH and Temperature Information using Magnetic Resonance Shift Imaging. *Chem. Eur. J.* **2017**, *23*, 7976.
12. Murray, B.S.; Parker, D.; dos Santos, C.M.G; Peacock, R.D. Synthesis, Chirality and Complexation Phenomena of Two Diastereoisomeric Dinuclear Lanthanide(III) Complexes. *Eur. J. Inorg. Chem*., **2010**, 2663-

2672.
13. Zheng, R. H.; Guo, H. C.; Jiang, H. J.; Xu, K. H.; Liu, B. B.; Sun, W. L.; Shen, Z. Q., *Chinese Chemical Letters,* **2010**, 21 (11), 1270-1272.
14. Ciruelos, S.; Doppiu, A.; Englert, U.; Salzer, A., New optically active 'constrained-geometry' cyclopentadienyl-phosphine ligands and their metal complexes. *J. Organomet. Chem*. 2002, 663 (1), 183-191.
15. Chooi, S. Y. M.; Leung, P. H.; Ng, S. C.; Quek, G. H.; Sim, K. Y., A simple route to optically pure 2,3-diaminobutane. *Tetrahedron. Asymmetry*. **1991**, 2 (10), 981-982.
16. Jirkovská, A.; Karabanovich, G.; Kubeš, J.; Skalická, V.; Melnikova, L.; Korábečný, J.; Kučera, T.; Jirkovský, E.; Nováková, L.; Bavlovič Piskáčková, H.; Škoda, J.; Štěrba, M.; Austin, C. A.; Šimůnek, T.; Roh, J. Structure–Activity Relationship Study of Dexrazoxane Analogues Reveals ICRF-193 as the Most Potent Bisdioxopiperazine against Anthracycline Toxicity to Cardiomyocytes Due to Its Strong Topoisomerase IIβ Interactions. *J. Med. Chem.* **2021**, 64 (7), 3997–4019.
17. Kovacs, Z.; Sherry, A. D. A general synthesis of mono- and disubstituted 1,4,7-triazacyclononanes. *Tetrahedron. Lett*. **1995**, 36(51): 9269-9272.
18. Bourdolle, A.; Allali, M.; Mulatier, J.C.; Le Guennic, B.; Zwier, J.M.; Baldeck, P.L.; Bünzli, J. C. G.; Andraud, C.; Lamarque, L.; Maury, O. Modulating the Photophysical Properties of Azamacrocyclic Europium Complexes with Charge-Transfer Antenna Chromophores. *Inorg. Chem.* **2011**, 50, 4987–4999.
19. Vallade, M.; Reddy, P. S.; Fischer, L.; Huc, I.; Enhancing Aromatic Foldamer Helix Dynamics to Probe Interactions with Protein Surfaces. *Eur. J. Org. Chem*., **2018**, 40, 5489-5498.
20. Cho, S. D.; Song, S. Y.; Park, Y. D.; Kim, J. J.; Joo, W. H.; Shiro, M.; Falck, J. R.; Shin, D. S. Novel synthesis of pyridazino[4,5-*b*][1,4]oxazin-3,8-diones. *Tetrahedron. Lett*. **2003**, 44(50): 8995-8998.
21. Mosandl, A. Über synthetische Aromastoffe, 4. Mitt. Diastereomere Amide der 3-Methyl-3-phenylglycidsäure. *Arch. Pharm. Pharm. Med. Chem*., **1984**, 317, 345-352.
22. Burdette, S. C.; Lippard, S. J. The Rhodafluor Family. An Initial Study of Potential Ratiometric Fluorescent Sensors for Zn^{2+}. *Inorg. Chem*. **2002**, 41, 25, 6816–6823.
23. Finney, K. -L. N. A.; Harnden, A. C.; Rogers, N. J.; Senanayake, P. K.; Blamire, A. M.; O'Hogain, D.; Parker, D. Simultaneous Triple Imaging with Two PARASHIFT Probes: Encoding Anatomical, pH and Temperature Information using Magnetic Resonance Shift Imaging. *Chem. Eur.*

J. **2017**, *23*, 7976.

24. Murray, B.S.; Parker, D.; dos Santos, C.M.G; Peacock, R.D. Synthesis, Chirality and Complexation Phenomena of Two Diastereoisomeric Dinuclear Lanthanide(III) Complexes. *Eur. J. Inorg. Chem*., **2010**, 2663-2672.
